# Pounding imparts internal strength to rubble-piles

**DOI:** 10.1038/s41598-026-39893-7

**Published:** 2026-02-20

**Authors:** J. Ormö, M. I. Herreros, R. Luther, K. Wünnemann, S. D. Raducan, M. Jutzi, L. M. Parro

**Affiliations:** 1 Centro de Astrobiología (CAB), CSIC-INTA, 28850 Torrejón de Ardoz, Spain; 2 Museum für Naturkunde, Leibniz Institute for Evolution and Biodiversity Science, Berlin, Germany; 3 Freie Universität Berlin, Berlin, Germany; 4 Space Research and Planetary Sciences, University of Bern, Bern, Switzerland; 5 IUFACyT, University of Alicante, Alicante, Spain

**Keywords:** Engineering, Natural hazards, Solid Earth sciences

## Abstract

It is known that nearly all 0.2–10 km, thus frequent, asteroids are "rubble-piles". However, their internal structure remains largely uncertain, but a strength-stratification with a weaker exterior compared with the interior has been suggested for e.g., Bennu. We use projectile impact experiments to explore a possible formation mechanism for this strength stratification. High-speed video recordings show that material from crushed target boulders and projectile rapidly penetrates radially beneath the floor of the expanding cavity in a porous target. Similar features are known from terrestrial impact structures where it penetrates fractures opening in the basement. On a rubble-pile asteroid, repeated impacts over time in a coarse, porous and easily crushable material could lead to accumulation of fine-grained, and increasingly compacted, thus stronger, material at depth. Meanwhile, impact-induced seismic activity on the asteroid, causing granular convection, along with rotational centrifugal forces, could promote the segregation of finer material beneath coarser layers, potentially making this effect more pronounced at the poles.

**Supplementary Information:**

The online version contains supplementary material available at 10.1038/s41598-026-39893-7.

## Introduction and aim

Recent visits by spacecrafts to the asteroids Itokawa, Bennu and Ryugu have led to their characterization as so-called “rubble-piles”. The importance of these objects constituting “an unorganized collection of macroscopic particles (rubble) held together by their self-gravity” have been known for several decades also from ground-based observations (See review by^1^). Indeed, rubble-piles may constitute nearly all the asteroids in the size range 0.2–10 km^1^ The reason for the high abundance has been suggested to be their high durability against disruption due to their shock-absorbent nature compared with monolithic bodies^2^. Still, there is great uncertainty about their internal configuration, and the possibility of a layered subsurface e.g. refs^3–5^.

In support of the DART and Hera missions we are performing laboratory impact experiment campaigns to study effects of boulders and other parameters such as target porosity and compaction on the cratering in “rubble-pile” target equivalents for the validation of numerical codes used in the analysis of the DART impact e.g. refs^6–9^. In the study presented here, we focus on clastic injections of crushed target material that we consider illustrates a plausible mechanism behind the apparent difference in material strength between the surface and the interior observed for some rubble-pile asteroids^4^, and likely also supported by the clastic injections observed in the true crater floor at terrestrial natural craters^10^. As the observed mechanism may correlate with the effects that the packing of clastic material has on the cratering, e.g. the formation of central mounds cf.^11^, we also included complementary experiments in homogeneous sand targets to study specifically the effect of packing.

**Fig. 1:**
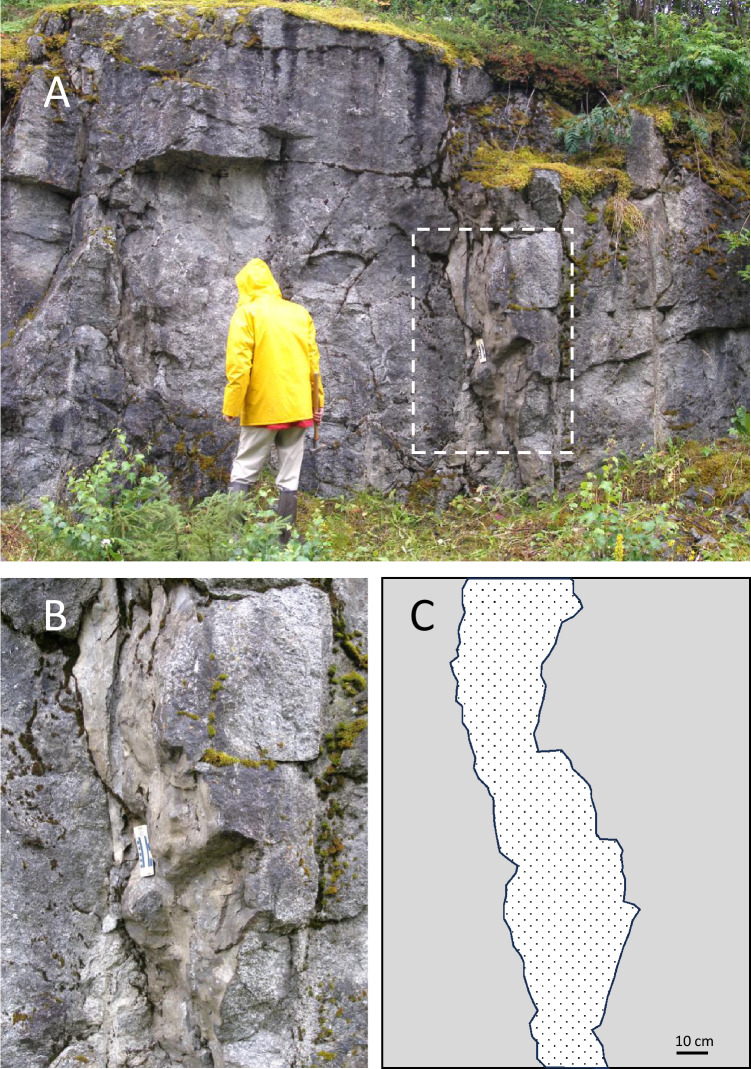
Clastic injection of mainly shocked upper target material penetrating into the fractured granitic true crater floor just inside the northern rim of the Lockne impact structure, Sweden. (**A**) Outcrop overview. The man in the image is 185 cm. (**B**) Magnification of stippled area in (**A)**. (**C**) Clarification of photo in (**B**). The injection is indicated with dotted fill inside granite (grey). Note standard USGS scale chart with 10 cm indication for scale. Photos: Jens Ormö.

During a cosmic impact fine-crushed and highly shocked material can be injected downwards into fractures that develop in the floor during the cavity expansion forming the transient crater, and even be part of the volume expansion causing the structural uplift of the crater rim that is so distinguishing for well-preserved terrestrial impact structures. Sturkell and Ormö^10^ reported centimetre- to decimetre-wide, and up to several meters long injections of shocked clastic material in the exposed fractured granitic true crater floor at the Lockne Crater, Sweden (Fig. 1). The source material was mainly from the upper target rocks, i.e. proximal to the impact point.

On the other hand, the effect of forcefully crushing and mobilizing material downwards in an already porous target, for instance, a rubble-pile asteroid, would be to fill pore space at depth with packed, fine-grained material. Daly et al.^4,12,13^ report how stronger material seems to exist at depth in the rubble-pile Bennu asteroid. This is indicated by the presence of a handful of so-called “central mound craters” out of a total of approximately 80 identified craters on Bennu. A central mound crater (hereafter CMC) is a known consequence of target strength layering, where a weak layer of a certain thickness relative to the crater diameter overlies a stronger layer^14^. This has been suggested as a cause for the CMCs on Bennu, in which a 1–4 m thick, finer-grained, more mobile layer would then need to cap a more competent interior of larger boulders e.g.,^15^.However, the mechanism requires that the fine-grained material constitutes the upper, weaker layer. However, Cline and Cintala^11^ saw in impact experiments into homogeneous (non-bedded) sand targets how factors such as internal friction and porosity (i.e., compaction) affect the generation of CMCs. Thus, CMCs may indicate not only strength layering as in the Quaide and Oberbeck model, but could also be the consequence of greater shear strength in a homogeneous granular material with high coefficient of static friction. A similar CMC was also formed by the Small Carry-on Impact (SCI) experiment carried out by the Hayabusa2 mission at rubble-pile asteroid Ryugu^3^.

The reasons for the apparent stratification of Bennu, Ryugu, and possibly other rubble-pile asteroids are not yet known. Based on the Quaide and Oberbeck model for CMC formation in a situation with underlying large boulders as suggested by Bierhaus et al.^15^, Daly et al.^4^ suggest that “the conditions for formation of craters with central mounds exist, if an appropriately sized and oriented boulder is located at the necessary depth”. Here we investigate if clastic injections can be a reason for the apparent strength-layering other than underlying larger boulders. The result would be much the same, e.g. CMCs, but the cause quite the opposite; i.e., a situation where the CMC does not form in an overlying, relatively lower-strength bed of boulders, but rather in a more compacted, finer-grained substrate. In this study we present a model based on clastic injections and seismic separation for how this substrate could form. The CMCs are here merely considered a consequence and, thus, indication for this mechanism. For a more detailed study of the formation of CMCs in homogeneous clastic materials, we refer to^11^.

## Results

**Fig. 2:**
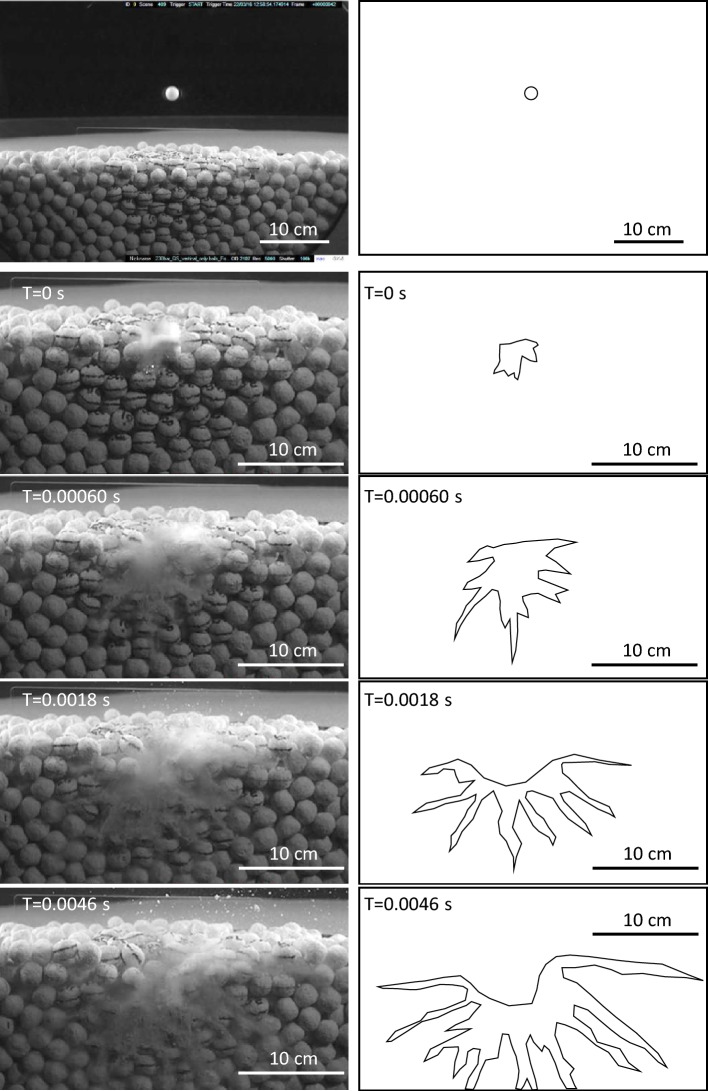
Clastic (dust) injections from impact into a “boulder” target made up of porous ceramic balls (Exp7), here simulating a relatively young, porous rubble-pile asteroid. Quarter-space target configuration. Impact velocity ~ 0.4 km/s (Delrin projectile). Left column shows snapshots from high-speed video. Crushed material penetrates radially downwards with a velocity of ~ 60 m/s. Right column clarifies the expansion of the clastic injections seen in the video frames.

**Fig. 3:**
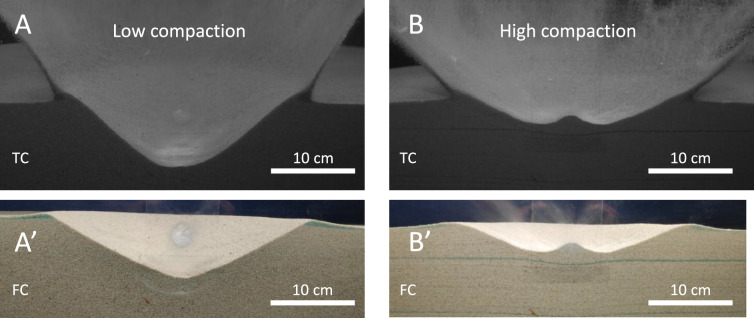
Effect of target sand compaction (i.e., porosity and internal friction) on the cratering. Centred at the point of impact is a screen protector glass**,** which obtains damage seen as a circular patch. Transient crater (TC) and final crater (FC). The low compaction target (Exp4) shown in plates A and A’ generates a relatively deeper crater and steeper ejection angle than the impact (Exp3) in the higher compaction target in plates B and B’. The crater in the higher compaction target also develops a “central mound” (or central hump as we suggest to call it). Photos in A’ and B’: Jens Ormö.

The result from the experiment with the boulder-filled bowl (Exp7) is shown in Fig. 2 and Supplementary Material. The cratering, i.e. the material displacement expressed by the observed topographic depression and the ejecta, is reduced compared to the reference sand target impact experiments shown in Fig. 3 and Supplementary Material (Exp3 and Exp4). However, of special interest to this study is how crushed target and projectile material, instead of being ejected or homogeneously displaced along the floor of the expanding cavity, penetrates with high velocity (here 60 ms^-1^) radially, and to great depth (here about 5 times the projectile diameter), into the porous target below the transient crater floor (Fig. 2, and Supplementary Material). Indeed, the floor of the expanding cavity is trailing behind the injections. During their penetration deeper into the target the injections are branching (Fig. 2). Altogether, this leads to the whole mechanism of injection-formation to migrate outwards until the increased surface friction halts further penetration.

The transient and final craters for our reference experiments (Exp3 and Exp4) with homogeneous target sand of different compaction are shown in Fig. 3A, B and Supplementary Material. Similarly to the observations by Cline and Cintala^11^, the crater in more compacted sand (Exp3, Fig. 3B) is smaller, especially shallower, than in the less compacted sand (Exp4, Fig. 3A). Notable is also the lower ejection angle and the CMC that develops in the high-compaction case. The comparison between Exp3 (high compaction, Fig. 3B) and Exp4 (low compaction, Fig. 3A) offers an alternative to the prevalent idea that central mounds form primarily in the weak layer overlying a stronger, i.e., the classic Quaide and Oberbeck concept of mounds developing in lunar regolith^14^. The craters in Fig. 3 are meant to illustrate the part of the crater formed in a deeper fine-grained bed after it has already been formed by injections from repeated impacts, likely coupled with the effect of compaction from the forceful material transport.

**Fig. 4:**
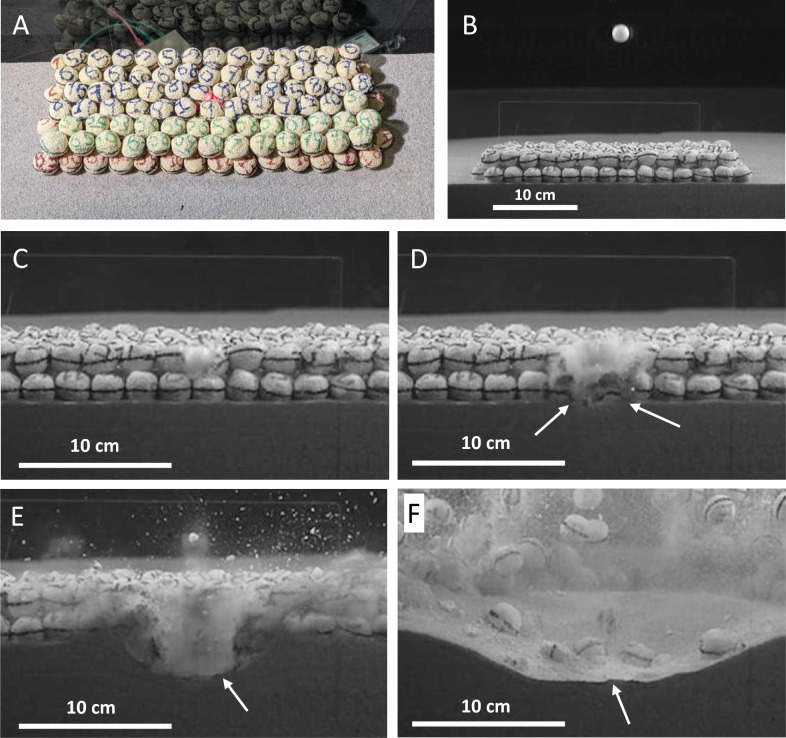
Impact into a stratified target (Exp6), here simulating a relatively mature rubble-pile asteroid in which a lower bed of fine-grained material has developed. Quarter-space target configuration. Impact velocity ~ 0.4 km/s (Delrin projectile). (**A**) Target set-up in oblique view. Photo: Jens Ormö. (**B**) Target set-up seen through the window of the camera tank. (**C**) Contact between projectile and the target. Crushing of projectile and struck boulder is initiated. (**D**) Continued crushing of boulders lead to dust injections (arrows). (**E**) Crushed projectile and boulder material is added to the floor of the expanding crater in the sand substrate (arrow). (**F**) A flat-floored crater with, possibly, a slight central mound (or rather hump) is formed in the sand and the added material from the crushed projectile and boulders (arrow).

In Exp6 we can see a combination of the previous experiments in that a boulder bed is covering a sand substrate (Fig. 4A and B, and Supplementary Material). The thickness of the boulder bed, and the level of compaction of the sand (i.e., “fast pour”, low-compaction) was selected to guarantee that cratering would occur in both the boulder bed (i.e., crushing and mobilization of boulder material) and the substrate. A thinner boulder bed would lead to less material from crushed boulders and a relatively large crater in the sand. A thicker boulder bed would generate a result closer to that shown in Fig. 2 (Exp7). In the continued sequence illustrated by frames C, D, and E of Fig. 4 we see how crushed projectile and boulder material is mobilized downwards and added to the substrate, initially as clastic injections, but then along the floor of the expanding cavity. We see that the crater in the sand (low-compaction) obtains a relatively flat-floored shape and is shallower than the reference crater in low compaction sand shown in Fig. 3A (Exp4), but there is only weak indication of a mound (Fig. 4F and Supplementary Material). A more compacted sand substrate (i.e., “slow pour”) would lead to less cratering of the sand, but likely more of a central mound as in Fig. 3B (Exp3). All in all, we find the set-up of the experiment shown in Fig. 4 (Exp6) a good approximation to achieve the objective to study material size segregation due to clastic injections.

## Discussion

**Fig. 5:**
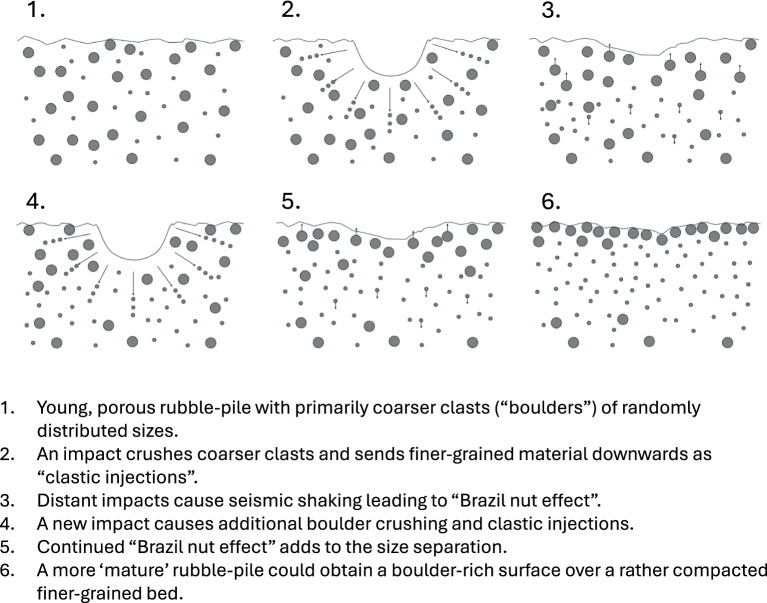
Effect of repeated impacts on the production of finer-grained material and development of rubble-pile target stratification. An impact into the target in plate 6 would possibly develop a “central mound crater” (or central hump crater as we here suggest could be a better term to avoid confusion with other formation mechanisms) in the more compacted, finer-grained bed (See Exp6 in Fig. 4 and Supplementary Material).

Figure 5 summarizes our experiments and illustrates the transformation from a relatively young, unstratified, porous rubble-pile (e.g., Dimorphos) where impact generated clastic injections can mobilize crushed material to deeper locations between randomly distributed boulders as seen in Fig. 2 (Exp7), as well as how the process may continue on more mature rubble-piles (e.g., Bennu) causing continued addition of crushed surface material to an existing, deeper bed of fine-grained material as illustrated in Fig. 4 (Exp6). It is a long-term evolutionary process resulting not from a single impact, but from the cumulative effect of maybe hundreds of impacts over time, combining the injection of fines with seismic shaking. Studies of the Itokawa asteroid by Miyamoto et al.^16^ indicate that seismic shaking of the rubble-pile by impacts could cause granular convection (“Brazil nut effect”) and redistribution of the coarser material relative to finer as even centimetre-sized impactors can globally induce seismic acceleration as large as the surface gravity on such a small body. Continued clastic injections could eventually lead to clogging of the pathways between subsurface boulders. However, the granular convection process of size segregation would continue to expand the surface boulder bed and generate new pathways for new impact injections adding to the previously injected material at depth as shown in Fig. 4 (Exp6).

The increased strength in the deeper positioned bed of fine-grained material causes the development of CMCs as observed in Fig. 3B (Exp3) and likely the SCI crater, as well as natural craters on asteroids such as Benny and Ryugu. In laboratory experiments, only partial stages of this evolution can be reproduced. In Fig. 5, Step 1 and 2 are illustrated by Exp7 (Fig. 2), but in mono-size-distribution of boulders. Step 3 and 5 are not part of the experimental series, but show the effect of seismic shaking by distant impacts. In step 4 a new impact as the one illustrated in Exp7 occurs, but now a lower fine-grained bed begins to evolve. Clastic injections from new impacts will reach down through the porous upper bed, and continue to add material to the fine-grained bed. Step 6 shows the target situation for Exp6 in Fig. 2. As illustrated in Fig. 2, boulders will again be crushed and material mobilized downwards to the fine-grained bed below, but clastic injections will now no longer be able to penetrate below the true crater floor of the crater in the finer grained bed (Fig. 4).

Bennu is known to be highly porous at a variety of scales^4,17,18^ with an extremely weak, almost cohesionless near-surface regolith^19–21^, but with an internal stiffness allowing mass wasting and surface cracks reaching global extent^18^. Daly et al.^4^ suggest that a significant difference in material properties may occur at a depth of a few meters. Zhang et al.^22^ describes how fine-grained material may percolate downwards driven by seismic shaking. If we consider a flow of impacts over time into such a porous material as a boulder-rich surface regolith, our suggested mechanism of forceful and deeply penetrating clastic injections as seen in Figs. 2, 4 and 5 should, with time, cause the accumulation of not only fine-grained, but compacted material at depth.

Depending on the balance between the asteroid’s gravity and the centrifugal forces generated by its rotation, an inverse segregation process could occur, in which finer grains migrate in the direction of the resulting force^23^. This would promote a stratification of finer material beneath coarser material to be more accentuated at the poles, with this tendency gradually decreasing towards the equator. Nevertheless, fluidization of fine particles due to prolonged vibrations on a small body like Itokawa can cause a global segregation forming rough topographically higher terrains, whereas finer particles migrate beneath and are exposed at potential low areas^16^. The forces caused by the rotation may also act to erase surface features such as impact craters^22^.

As a consequence of this material mobilization, it would be natural to think that the combination of a compacted, relatively fine-grained interior and a looser, bouldery exterior is responsible for the formation of CMCs on rubble-pile asteroids. For asteroids in the size range of Ryugu and Bennu, impact cratering occurs within the strength-dominated regime at the body scale, because their bulk gravitational acceleration is too low for gravity scaling to prevail. However, within the upper layers of granular, cohesionless material, the local cratering process remains gravity-dominated, as demonstrated by the Small Carry-on Impactor (SCI) experiment on Ryugu^3^. In other words, while the overall impact energy scales with target strength, the granular surface still behaves according to gravity-controlled excavation dynamics. However, if a deeper, compacted layer of fine-grained material on an asteroid indeed is stronger than the upper layer of loose boulders, a central mound could also form in that deeper layer as shown in Fig. 3B (Exp3) and Fig. 4 (Exp6). Indeed, like the crater in our Exp6, the mounds in the CMCs on Ryugu and Bennu seem to occur in the substrate, not in the overlying boulder layer. Likewise, images of the SCI crater interior (e.g., Fig, 1a & 1b in^3^) show structures that seem consistent with Step 6 of our model (Fig. 5).

Our reference parameter experiments in sand at different degrees of compaction (i.e., “slow-pour” vs. “fast-pour”, Fig. 3A,B, Supplementary Material) successfully reproduced the morphologies observed also at higher velocities (i.e., ~ 1.55 km s^−1^) by Cline and Cintala (^11^, their Fig. 7)^3^. In particular, a well-defined central mound developed in the high-compaction target, consistent with their interpretation of mound formation resulting from greater shear strength in more compacted granular media. As the horizontal, coloured sand layer below the final crater in Fig. 3 indicates near absence of displacement at depth, and that the target was homogeneous, we can exclude a formation following the mechanism of “central peak” formation in complex impact craters by material uplift e.g. review by^24^, as well as a “central mound” formation due to strength layering e.g.^14^. As none of these mechanisms apply to the observed morphological feature, we suggest that *central hump* may be a better term.

**Fig. 6:**
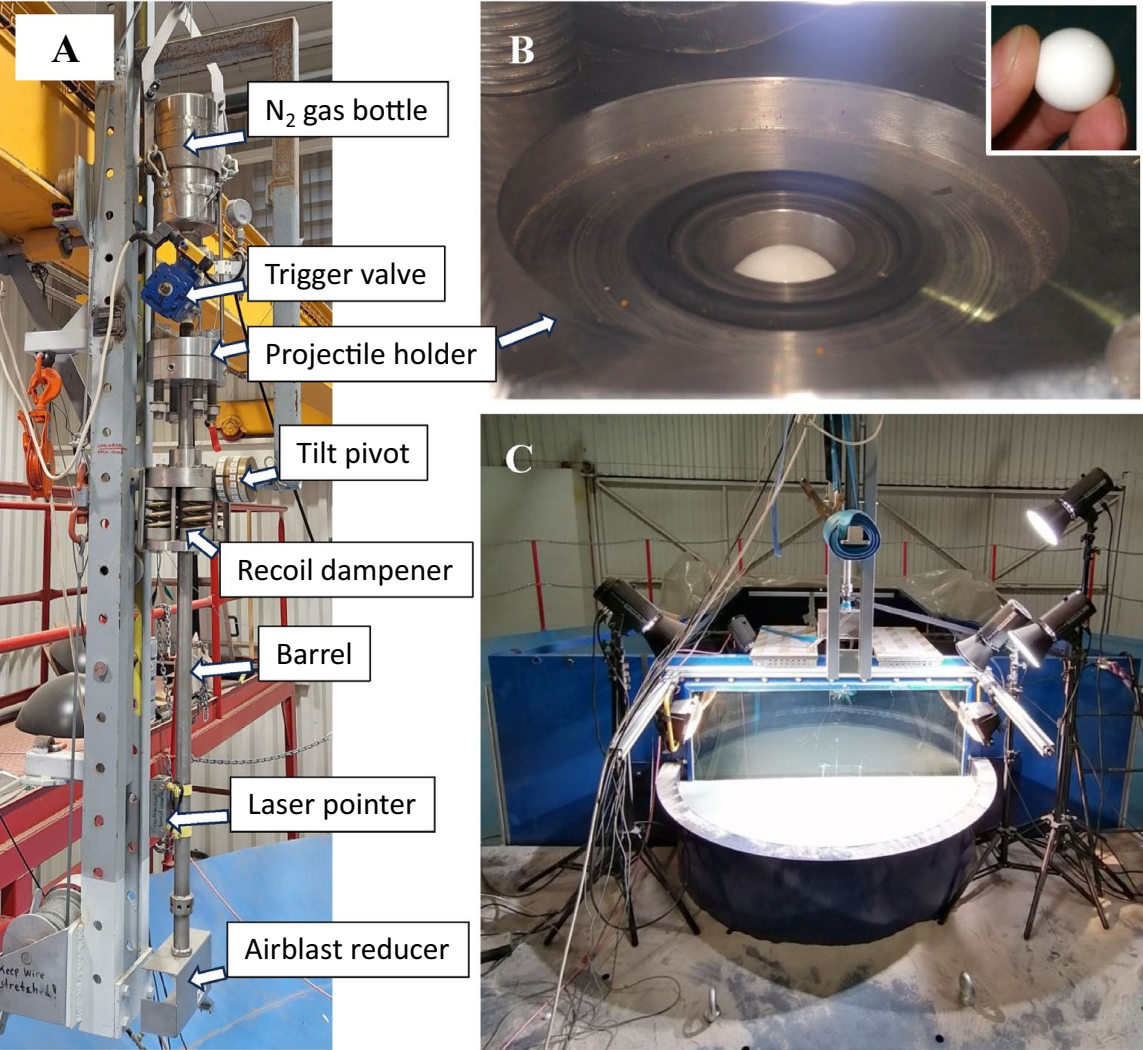
Experimental Projectile Impact Chamber (EPIC) at CAB CSIC-INTA. (**A**) The compressed N_2_ gun (It can be tilted for oblique impact experiment. For further details see Ormö et al., 2015). Photo: Jens Ormö. (**B**) Massive 20 mm Delrin ball loaded into the gun (Inset for scale). Photos: Jens Ormö. (**C**) The test-bed with filled sand target holder. Behind the sand target holder is the 5 cm thick glass window of the camera tank allowing quarter-space experiments. Photo: Isabel Herreros.

In any case, a way of obtaining the prerequisite for either “mound” or *hump* craters could be the mechanism of production of fine particles and their accumulation and compaction from forceful and deep-penetrating clastic injections by repeated impacts over time. It should be noted that for central mounds to be unequivocally recognized in both experimental and natural impact structures they should be sufficiently larger than other objects in the target that may generate similar features, e.g. boulders.

Altogether, the mechanism of clastic injections provides a viable cause for observed stratification of ‘mature’ rubble-pile asteroids such as Ryugu and Bennu, and offers a better explanation for the CMCs (or central “hump” craters) that seemingly have developed in the finer-grained substrate rather than in a looser, bouldery upper layer as expected if formed according to the typical Quaide and Oberbeck model^14^.

## Methods

In support of the DART and Hera missions to Dimorphos, we run campaigns that include series of experiments with various target configurations in order to validate the numerical codes used to simulate the DART impact e.g.^6^. The experiments are carried out in the Experimental Projectile Impact Chamber (EPIC) at Centro de Astrobiología (CAB), CSIC-INTA, in Spain cf.^25^ (Fig. 6). Detailed descriptions of EPIC, the projectiles, and the target materials are provided in^6,25^ and in the Supplementary Material. The set-up of four of the experiments are described here, i.e., Exp3, Exp4, Exp6, and Exp7. For further details see Supplementary Material. The numbering of experiments in this study is what was given to the experiments in the respective experiment campaign. Not all experiments in each campaign are relevant for this study, and even failed shots are numbered. Nevertheless, we prefer to maintain the original numbering, even of failed experiments, to avoid confusion with forthcoming analyses. This explains the seemingly inconsistent numbering in this study.

For the experiment Exp7 (Fig. 2) the target was prepared in a 60 ‐cm wide, half‐spherical metal bowl that had been cut in half and mounted to the window of the high-speed camera chamber for quarter-space setup. This window is 5 cm thick to avoid loss of energy due to vibrations. The bowl was filled with porous, ceramic balls of nearly equal diameter and density as the projectile. For detailed description of the balls see^6^ in which it is stated that balls are approximately 2.25 cm in diameter, have a tested compressive strength of ≈1 MPa, and a porosity of about 66%. Notwithstanding differences in chemical composition, Ormö et al.^6^ considered the ceramic material to be a good mechanical analogue for the boulders found on the rubble-pile asteroid Bennu, as the mechanical strength is within the 0.44–1.7 MPa estimated for the boulders on Bennu^26^. There was no finer matrix filling out the pore space between the boulders, thus creating a bulk porosity of ~ 80%. After seeing the results from Exp7 we felt there could be a connection to the aforementioned observations of craters seemingly affected by potential stratification (be it from strength, compaction, or a combination) in rubble-pile asteroids. As the effect of strength-layering on cratering is fairly well known from several studies e.g.^14,27^, but debated for the cosmic bodies of such low gravity, we decided to compare with tests similar to those described by^11^. Two reference shots (logged as Exp3 and Exp4, Fig. 3) were made to test the effect of sand with known difference in compaction cf.^25,28^: “slow pour” (applied by raining down the sand, bulk density: ~1.8 g/cm3, porosity: ~32%, friction angle: 34.6 ± 0.8°), and “fast pour” (bulk density: ~1.6 g/cm3, porosity: ~40%, friction angle: 30.4 ± 1.7°). Likewise, when combining the observations made from Exp7 with those of Exp3 and Exp4, we found that the Exp6 would provide further support to the investigated concept (Fig. 4). The target set-up of Exp6 allows the analysis of the effects of a porous, boulder bed (here ceramic balls) overlying a substrate of fine-clastic material (here “fast-pour sand”) (Fig. 4, Supplementary Material).

The projectiles in all shots were of Delrin (20 mm diameter, 5.7 g) and impacting at ~ 0.4 km/s. Studies by ourselves e.g.^6,25^ and others e.g.^29^ their Fig. 2, have shown that craters generated in the target materials used in this study scale well with those from impacts into equivalent targets at higher velocities. Here, the impact velocity is calculated following^25^ involving an experimentally established relation between projectile velocity, gas pressure and distance to target, as well as measurements in images of distance and time given by camera frames in the image analysis software jMicrovision (version 1.2.7). This software was also used to estimate the distance and velocity with which the injections propagate into the target (Fig. 2). The experiments were made in quarter-space setup and recorded with a NAC Gx8 high-speed camera here set to a recording with 5000 frames per second (fps).

## Supplementary Information

Supplementary Information.

## Data Availability

Data is provided within the manuscript or supplementary information files.

## References

[1] Walsh, K. Rubble pile asteroids. *Annu. Rev. Astron. Astrophys.*10.1146/annurev-astro-081817-052013 (2018).

[2] Jourdan, F. et al. Rubble pile asteroids are forever. *Proc. Natl. Acad. Sci. U.S.A.* **120**(5), e2214353120. 10.1073/pnas.2214353120 (2023). 36689662 10.1073/pnas.2214353120 PMC9945955

[3] Arakawa, M. et al. An artificial impact on the asteroid 162173 Ryugu formed a crater in the gravity-dominated regime. *Science* **368**(6486), 67–71. 10.1126/science.aaz1701 (2020). 32193363 10.1126/science.aaz1701

[4] Daly, R. T. et al. The morphometry of small impact craters on Bennu: Relationships to geologic units, boulders, and impact armoring. *Icarus*10.1016/j.icarus.2022.115058 (2022).

[5] DellaGiustina, D. N. et al. Seismology of rubble-pile asteroids in binary systems. *Mon. Not. R. Astron. Soc.* **528**(4), 6568–6580. 10.1093/mnras/stae325 (2024).

[6] Ormö, J. et al. Boulder exhumation and segregation by impacts on rubble-pile asteroids. *Earth Planet. Sci. Lett.*10.1016/j.epsl.2022.117713 (2022).

[7] DeCoster, M. E., Stickle, A. M., Rainey, E. S. G. & Graninger, D. M. Statistical analysis of near-surface structure and material properties on momentum transfer in rubble pile targets impacted by kinetic impactors. *Planet. Sci. J.*10.3847/PSJ/ad7cff (2024).

[8] Luther, R. et al. Momentum enhancement during kinetic impacts in the low-intermediate-strength regime: Benchmarking and validation of impact shock physics codes. *Planet. Sci. J.* **3**(10), 227. 10.3847/PSJ/ac8b89 (2022).

[9] Luther, R. et al. Impact experiments and model validation in the frame of the hera mission. Abstract presented at the europlanet science congress, #10.5194/epsc2024-194 10.5194/epsc2024-194 (Berlin, 2024).

[10] Sturkell, E. F. F. & Ormö, J. Impact-related clastic injections in the marine Ordovician Lockne impact structure, central Sweden. *Sedimentology* **44**, 793–804. 10.1046/j.1365-3091.1997.d01-54.x (1997).

[11] Cline, C. J. & Cintala, M. J. The effects of target density, porosity, and friction on impact crater morphometry: Exploratory experimentation using various granular materials. *Meteorit. Planet. Sci.* **57**(8), 1537–1552. 10.1111/maps.13886 (2022).

[12] Daly, M. G. et al. Hemispherical differences in the shape and topography of asteroid (101955) Bennu. *Sci Adv.*10.1126/sciadv.abd3649 (2020). 33033038 10.1126/sciadv.abd3649 PMC7544500

[13] Daly, R. T. et al. The morphometry of impact craters on Bennu. *Geophys. Res. Lett.*10.1029/2020GL089672 (2020).

[14] Quaide, W. L. & Oberbeck, V. R. Thickness determinations of the lunar surface layer from lunar impact craters. *J. Geophys. Res.* **73**(16), 5247–5270. 10.1029/jb073i016p05247 (1968).

[15] Bierhaus, E. B. et al. A subsurface layer on asteroid (101955) Bennu and implications for rubble pile asteroid evolution. *Icarus* **406**, 115736 (2023).

[16] Miyamoto, H. et al. Regolith migration and sorting on asteroid Itokawa. *Science* **316**, 1011–1014. 10.1126/science.1134390 (2007). 17446355 10.1126/science.1134390

[17] Barnouin, O. S., Daly, R. T., Cintala, M. J. & Crawford, D. A. Impacts into coarse-grained spheres at moderate impact velocities: Implications for cratering on asteroids and planets. *Icarus* **325**, 67–83. 10.1016/j.icarus.2019.02.004 (2019).

[18] Barnouin, O. S. et al. Shape of (101955) Bennu indicative of a rubble pile with internal stiffness. *Nat. Geosci.* **12**, 247–252. 10.1038/s41561-019-0330-x (2019). 31080497 10.1038/s41561-019-0330-x PMC6505705

[19] Barnouin, O. S. et al. The formation of terraces on asteroid (101955) Bennu. *J. Geophys. Res. Planets.*10.1029/2021JE006927 (2022).

[20] Perry, M. et al. Low surface strength of the asteroid Bennu inferred from impact ejecta deposit. *Nat. Geosci.* **15**, 1–6. 10.1038/s41561-022-00937-y (2022).

[21] Lauretta, D. S. et al. Spacecraft sample collection and subsurface excavation of asteroid (101955) Bennu. *Science* **377**(6603), 285–291. 10.1126/science.abm1018 (2022). 35857591 10.1126/science.abm1018

[22] Zhang, Y. et al. Inferring interiors and structural history of top-shaped asteroids from external properties of asteroid (101955) Bennu. *Nat. Commun.* **13**(1), 4589. 10.1038/s41467-022-32288-y (2022). 35933392 10.1038/s41467-022-32288-y PMC9357032

[23] Guibout, V. & Scheeres, D. J. Stability of Surface motion on a rotating ellipsoid. *Celest. Mech. Dyn. Astron.* **87**(3), 263–290. 10.1023/B:CELE.0000005720.09027.ee (2003).

[24] Hay, H. C. F. C., Collins, G. S., Davison, T., Rajšić, A. & Johnson, B. C. Complex crater collapse: A comparison of the block and Melosh acoustic fluidization models of transient target weakening. *J. Geophys. Res. Planets.*10.1029/2024JE008544 (2024). 39678356 10.1029/2024JE008544 PMC11645986

[25] Ormö, J. et al. Scaling and reproducibility of craters produced at the Experimental Projectile Impact Chamber (EPIC), Centro de Astrobiología. *Spain. Meteorit. Planet. Sci.* **50**(12), 2067–2086. 10.1111/maps.12560 (2015).

[26] Ballouz, R. L. et al. Bennu’s near earth lifetime of 1.75 million years inferred from craters on its boulders. *Nature*10.1038/s41586-020-2846-z (2020). 33106686 10.1038/s41586-020-2846-z

[27] Prieur, N. C., Rolf, T., Wünnemann, K. & Werner, S. C. Formation of simple impact craters in layered targets: Implications for lunar crater morphology and regolith thickness. *J. Geophys. Res. Planets* **123**(6), 1555–1578. 10.1029/2017JE005463 (2018).

[28] Ormö, J. et al. Effect of target layering in gravity-dominated cratering in nature, experiments, and numerical simulations. *J. Geophys. Res. Planets.*10.1029/2023JE008110 (2024).

[29] Miranda, C. S. & Dowling, D. R. Mach number scaling of impact craters in unconsolidated granular materials. *Icarus* **325**, 84–93. 10.1016/j.icarus.2019.02.006 (2019).

