# Supplementary material for: Pounding imparts internal strength to rubble-piles
Source: Sci Rep. 2026 Feb 20;16:10054. doi: 10.1038/s41598-026-39893-7 (PMC13022226; doi:10.1038/s41598-026-39893-7)

Supplementary material

Laboratory impact experiments

Projectile launcher:
Single stage 300bar compressed N_2

Projectile:
- Delrin
- 20 mm diameter
- 5.7g
- 1.36g/cm^3
- Velocity: max.~420 m/s
  In these experiments:~408 m/s

Recording device:
- NAC Gx8 high-speed camera here set to 5000 fps

Target sand:
- Density (fast pour): ~1.6 g/cm^3
- Porosity (fast pour): ~40%
- Friction angle (fast pour): 30.4 ± 1.7°
- Density (slow pour): ~1.8 g/cm^3
- Porosity (slow pour): ~32%
- Friction angle (slow pour): 34.6 ± 0.8°

Target balls:
- Mass: 5.7 ±0.5 g
- Diameter: 2.25 ±0.16 cm
- Density: ~0.96 g/cm^3
- Porosity: ~66%
- Compressive crush strength:~1 Mp

All photos are captured by the authors

“Exp 7”: Homogeneous target with projectile-size boulders and no fine matrix

Projectile:

- Delrin (20 mm diameter)
- 5.7g
- 1.36g/cm^3
- Velocity: ~408 m/s

Target balls:

- Mass: 5.7 ±0.5 g
- Diameter: 2.25 ±0.16 cm
- Density: ~0.96 g/cm^3
- Porosity: ~66%
- Compressive crush strength:~1 Mp

“Exp 7”.

Before

After

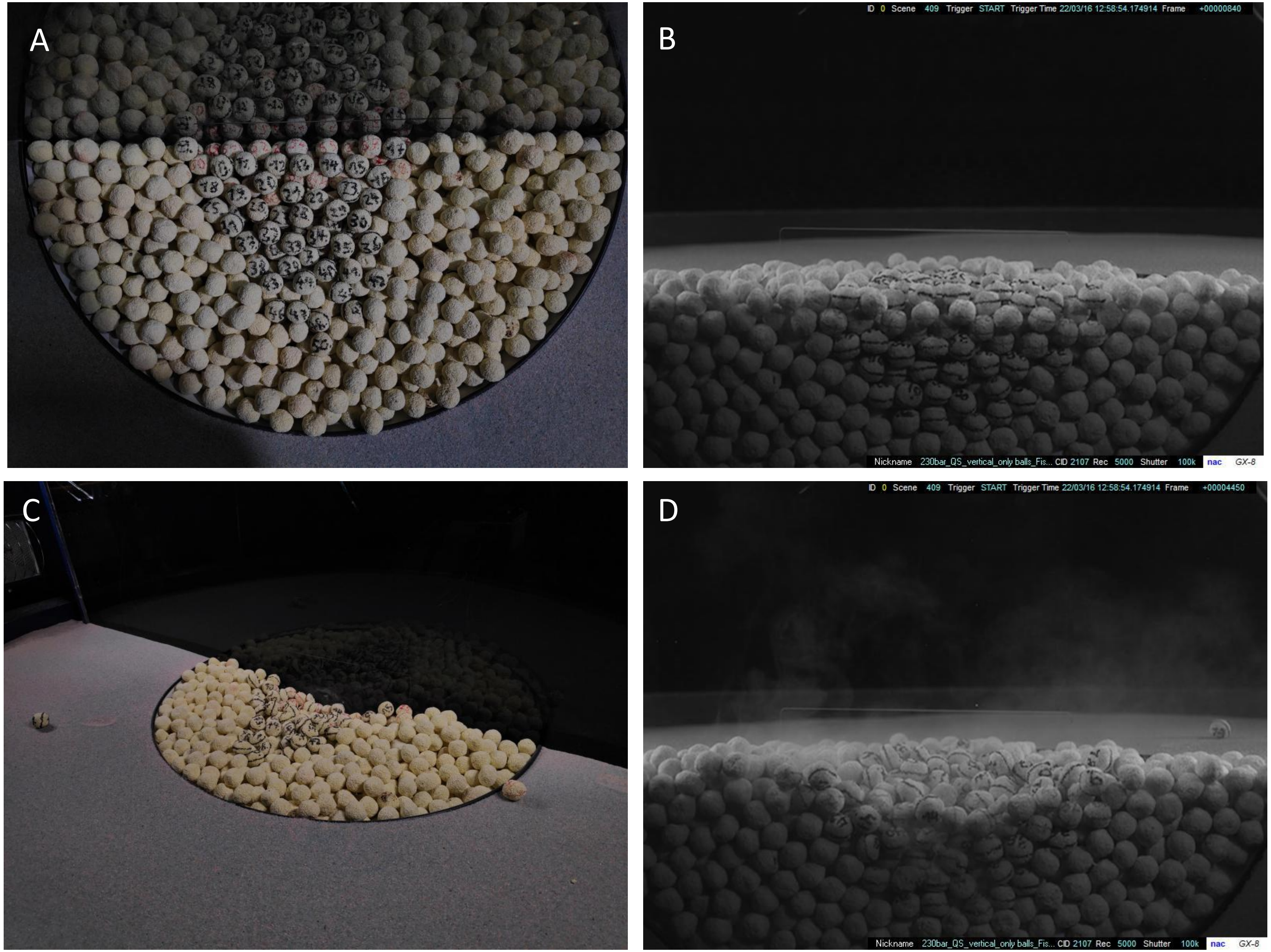

Relatively reduced excavation due to energy is consumed in crushing of boulders

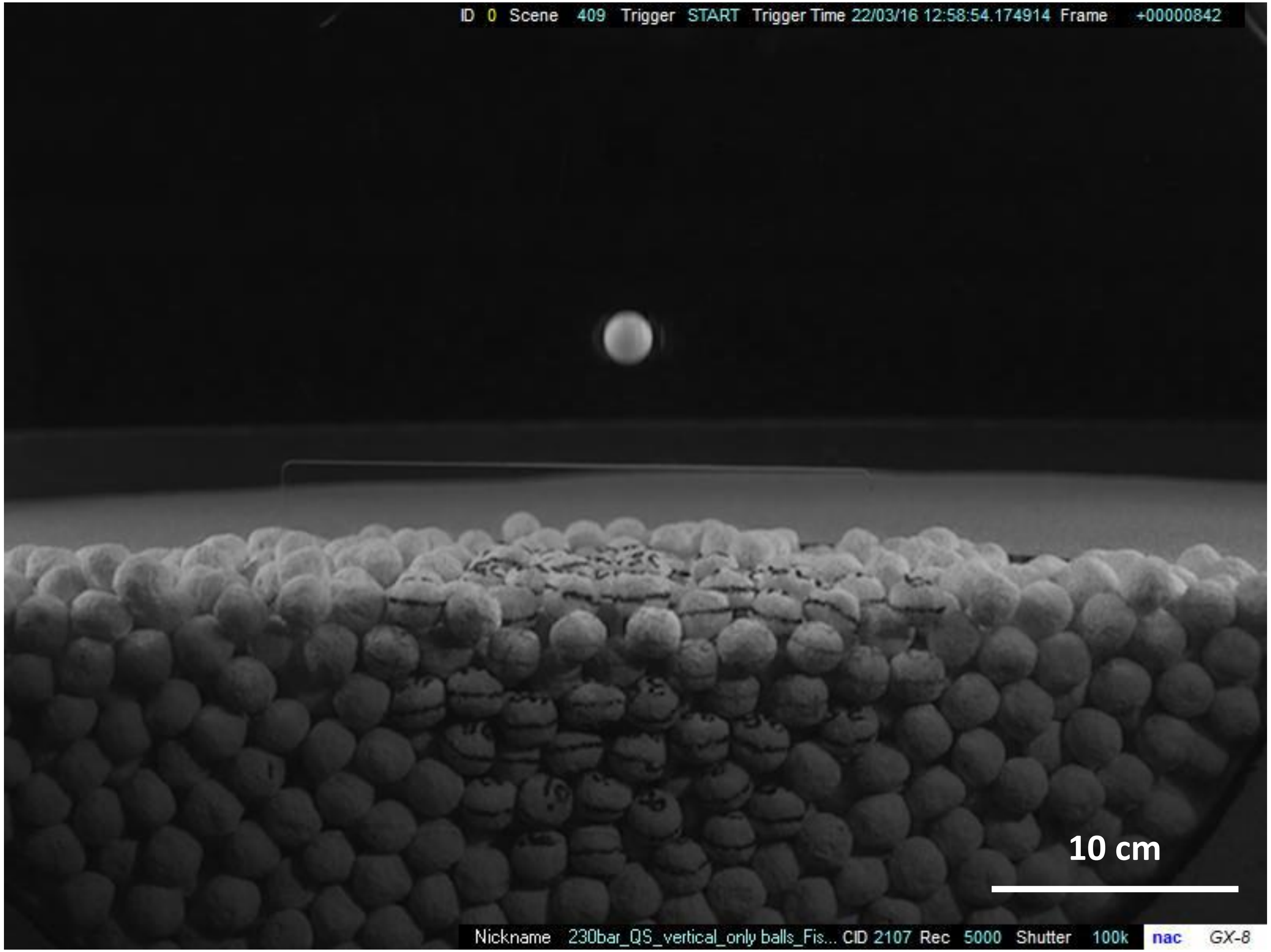
ID 0 Scene 409 Trigger START Trigger Time 22/03/16 12:58:54.174914 Frame +00000842
10 cm
Nickname 230bar_QS_vertical_only balls_Fis... CID 2107 Rec 5000 Shutter 100k nac GX-8

“Exp 7”.

“Exp 7”.

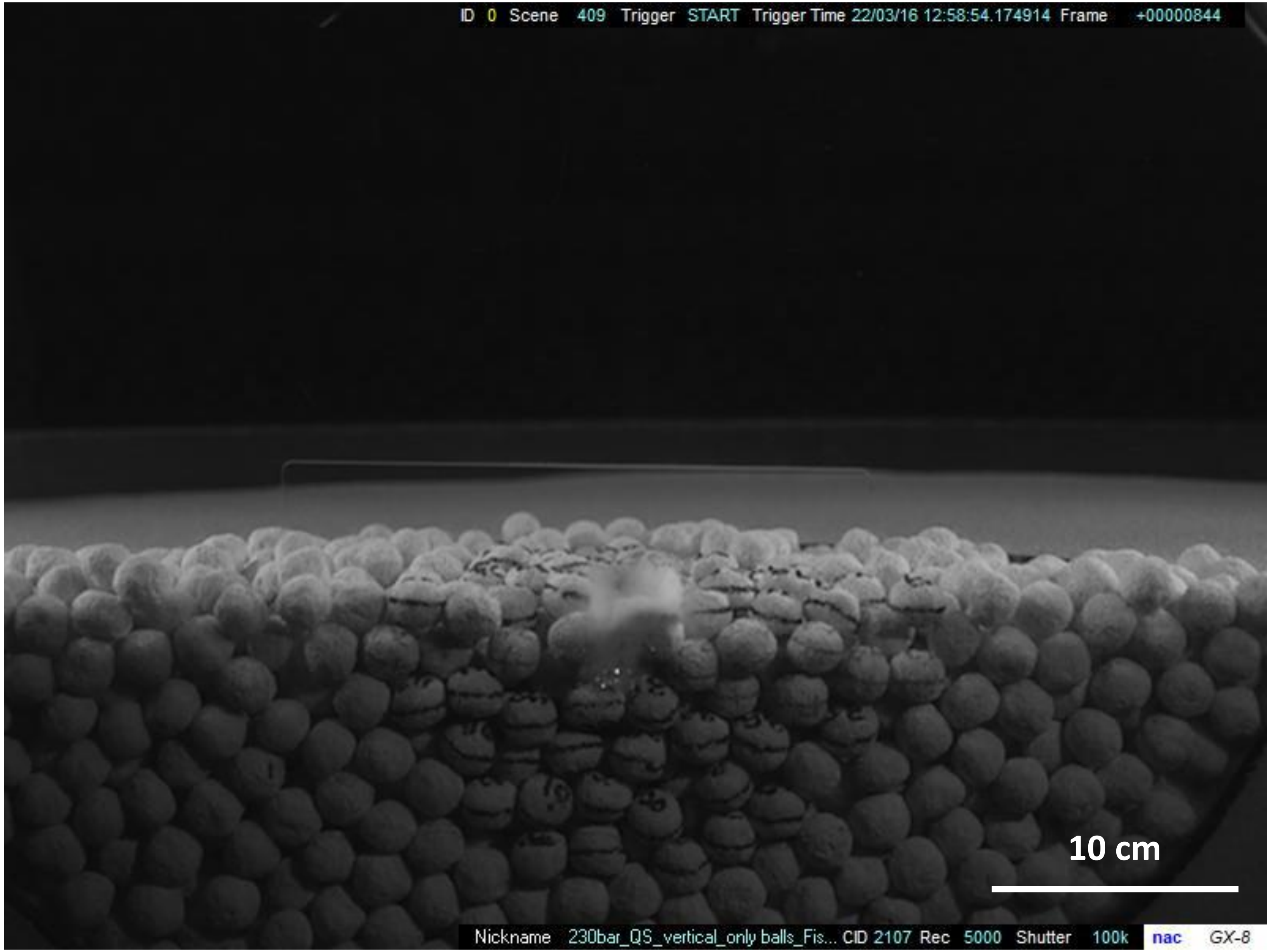
ID 0 Scene 409 Trigger START Trigger Time 22/03/16 12:58:54.174914 Frame +00000844
10 cm
Nickname 230bar_QS_vertical_only balls_Fis... CID 2107 Rec 5000 Shutter 100k nac GX-8

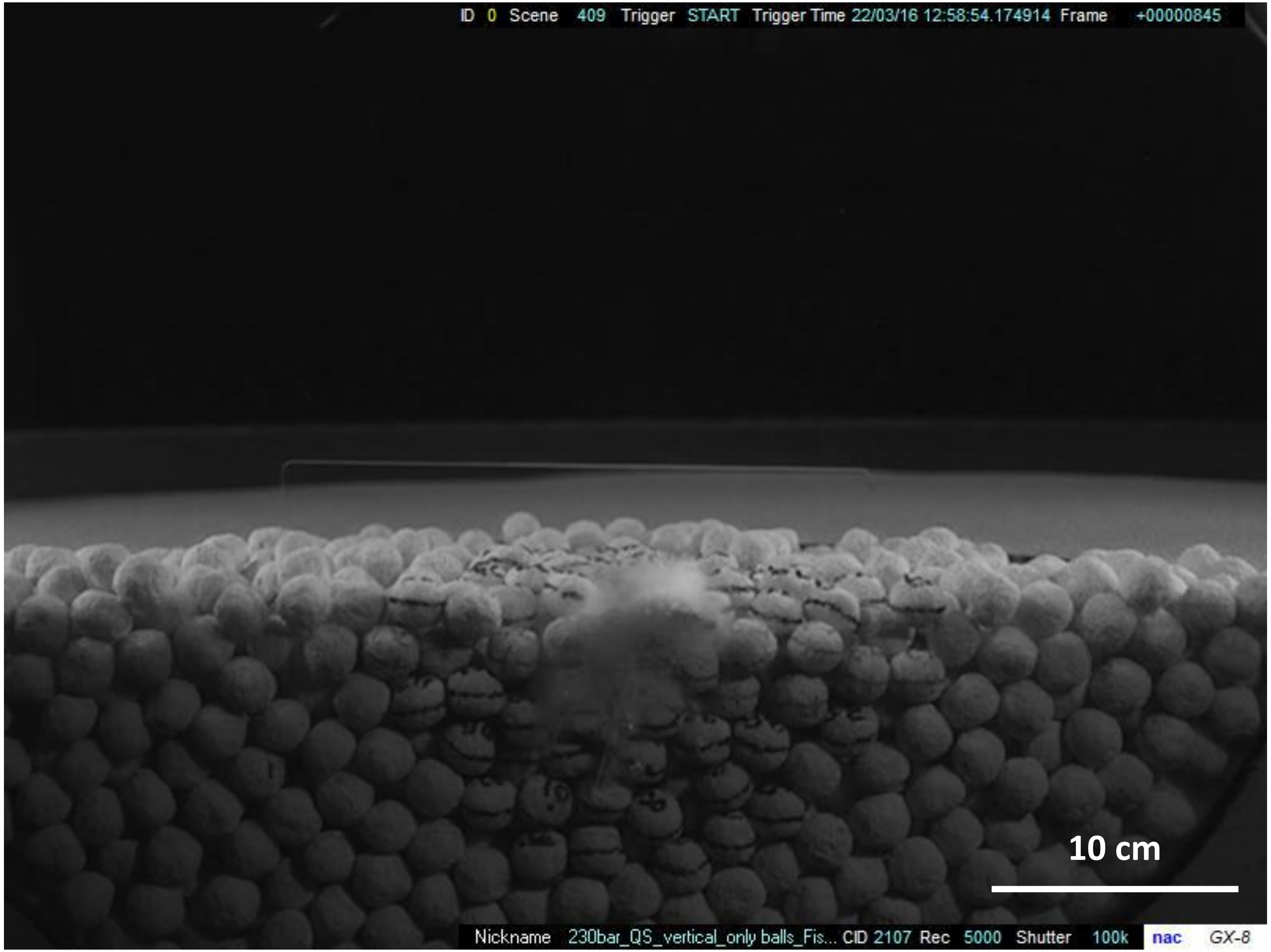
ID 0 Scene 409 Trigger START Trigger Time 22/03/16 12:58:54.174914 Frame +00000845
10 cm
Nickname 230bar_QS_vertical_only balls_Fis... CID 2107 Rec 5000 Shutter 100k nac GX-8

“Exp 7”.

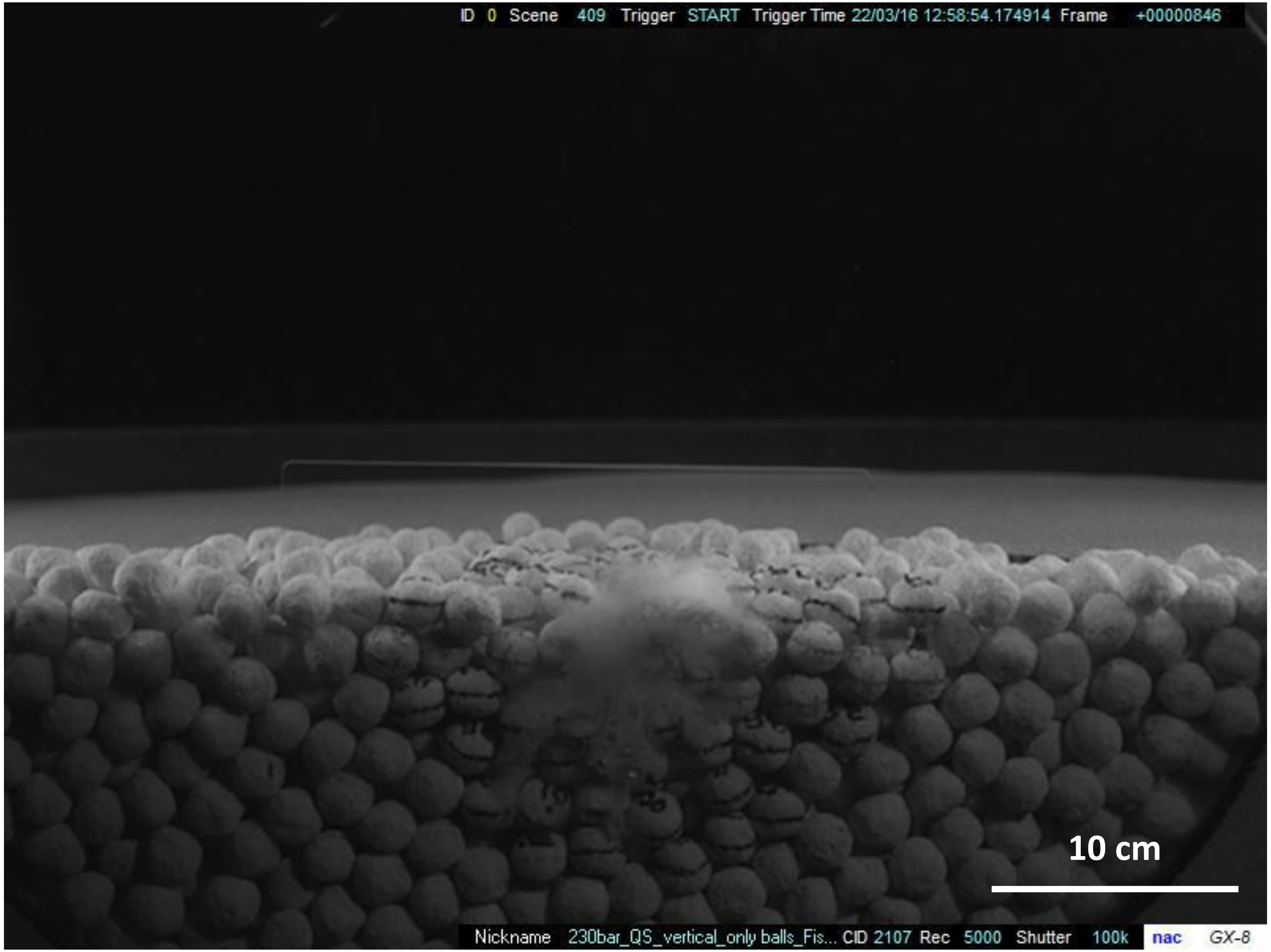
ID 0 Scene 409 Trigger START Trigger Time 22/03/16 12:58:54.174914 Frame +00000846
10 cm
Nickname 230bar_QS_vertical_only balls_Fis... CID 2107 Rec 5000 Shutter 100k nac GX-8

“Exp 7”.

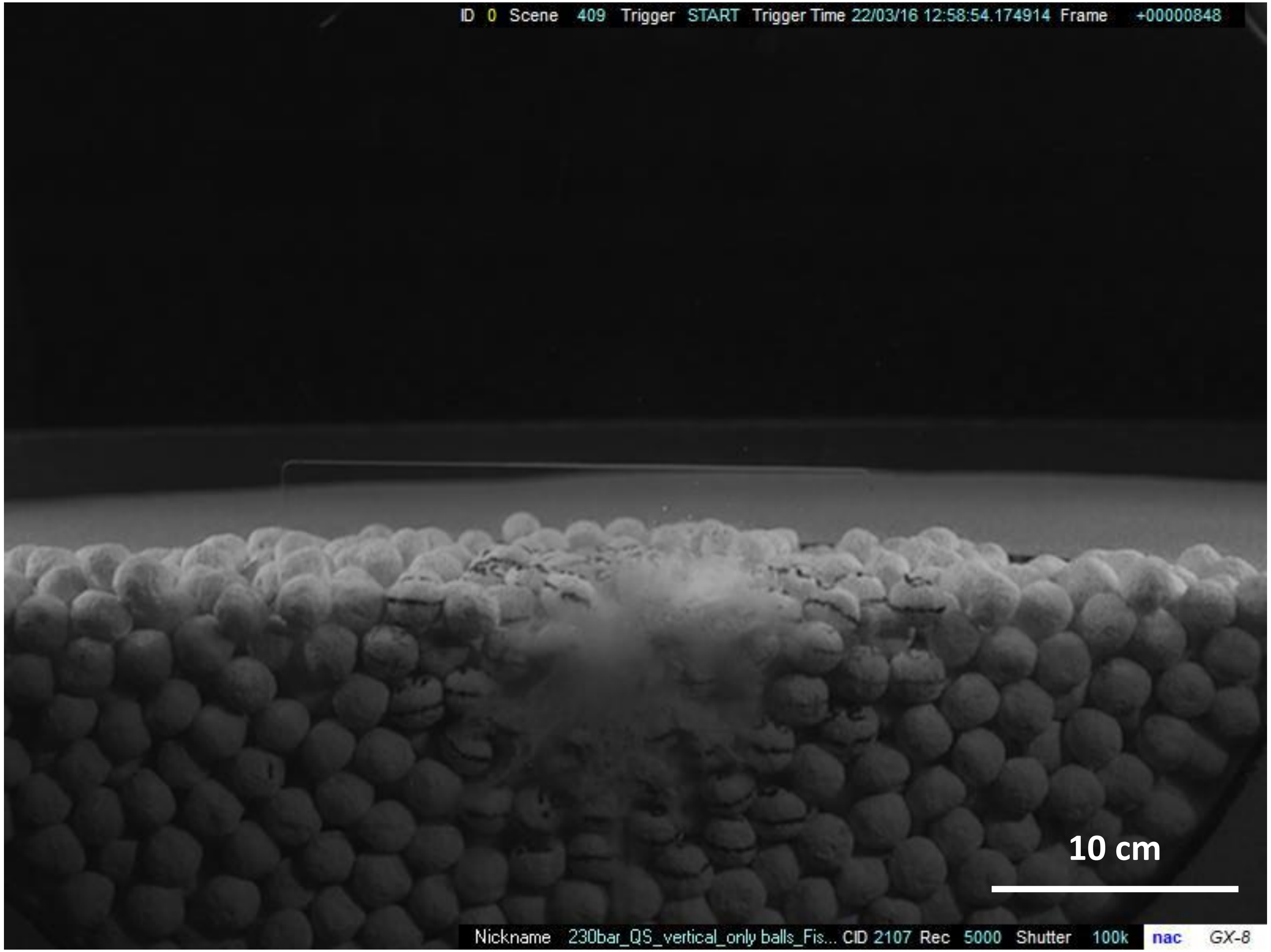
ID 0 Scene 409 Trigger START Trigger Time 22/03/16 12:58:54.174914 Frame +00000848
10 cm
Nickname 230bar_QS_vertical_only balls_Fis... CID 2107 Rec 5000 Shutter 100k nac GX-8

"Exp 7".

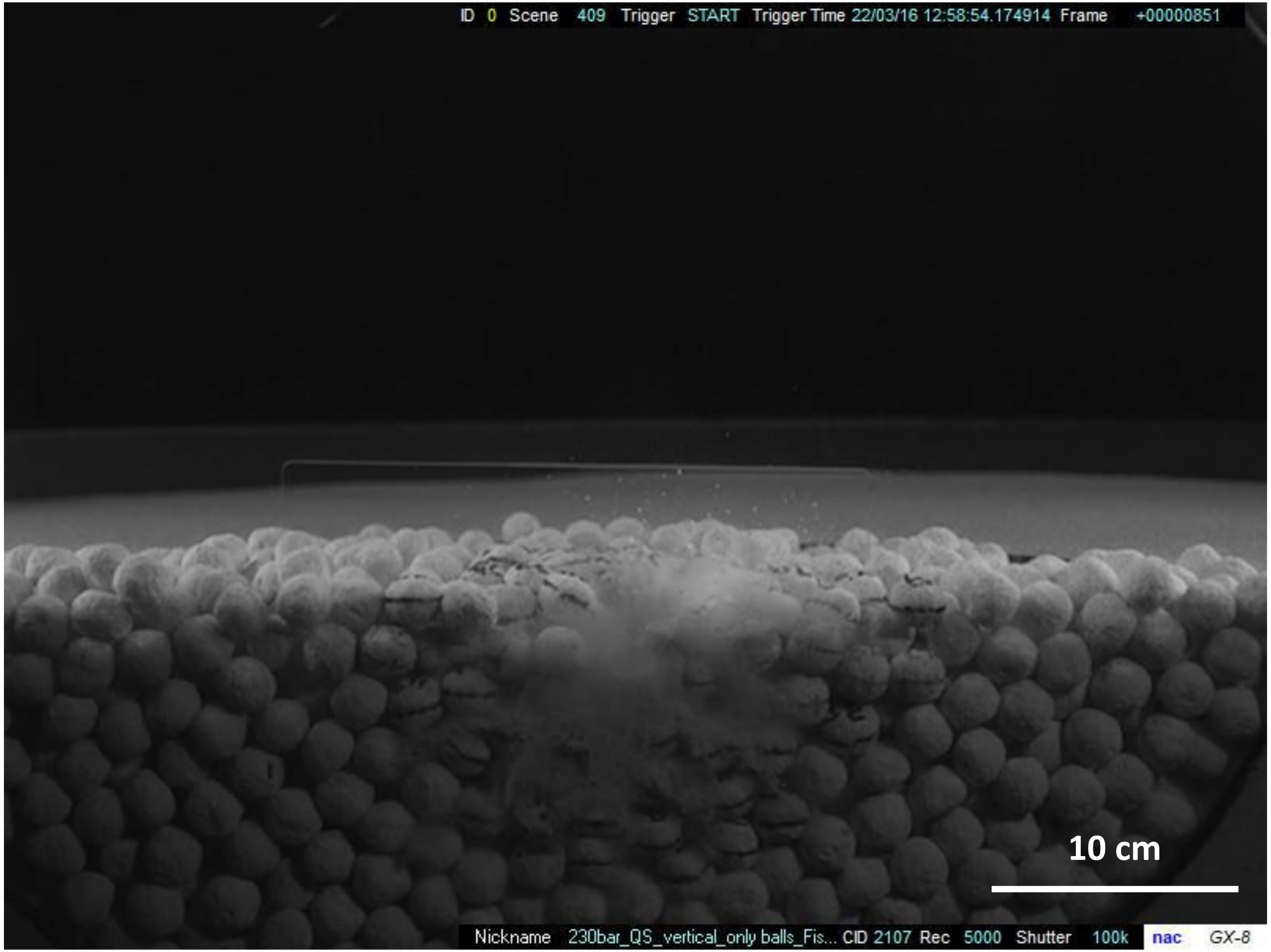
ID 0 Scene 409 Trigger START Trigger Time 22/03/16 12:58:54.174914 Frame +00000851
10 cm
Nickname 230bar_QS_vertical_only balls_Fis... CID 2107 Rec 5000 Shutter 100k nac GX-8

“Exp 7”.

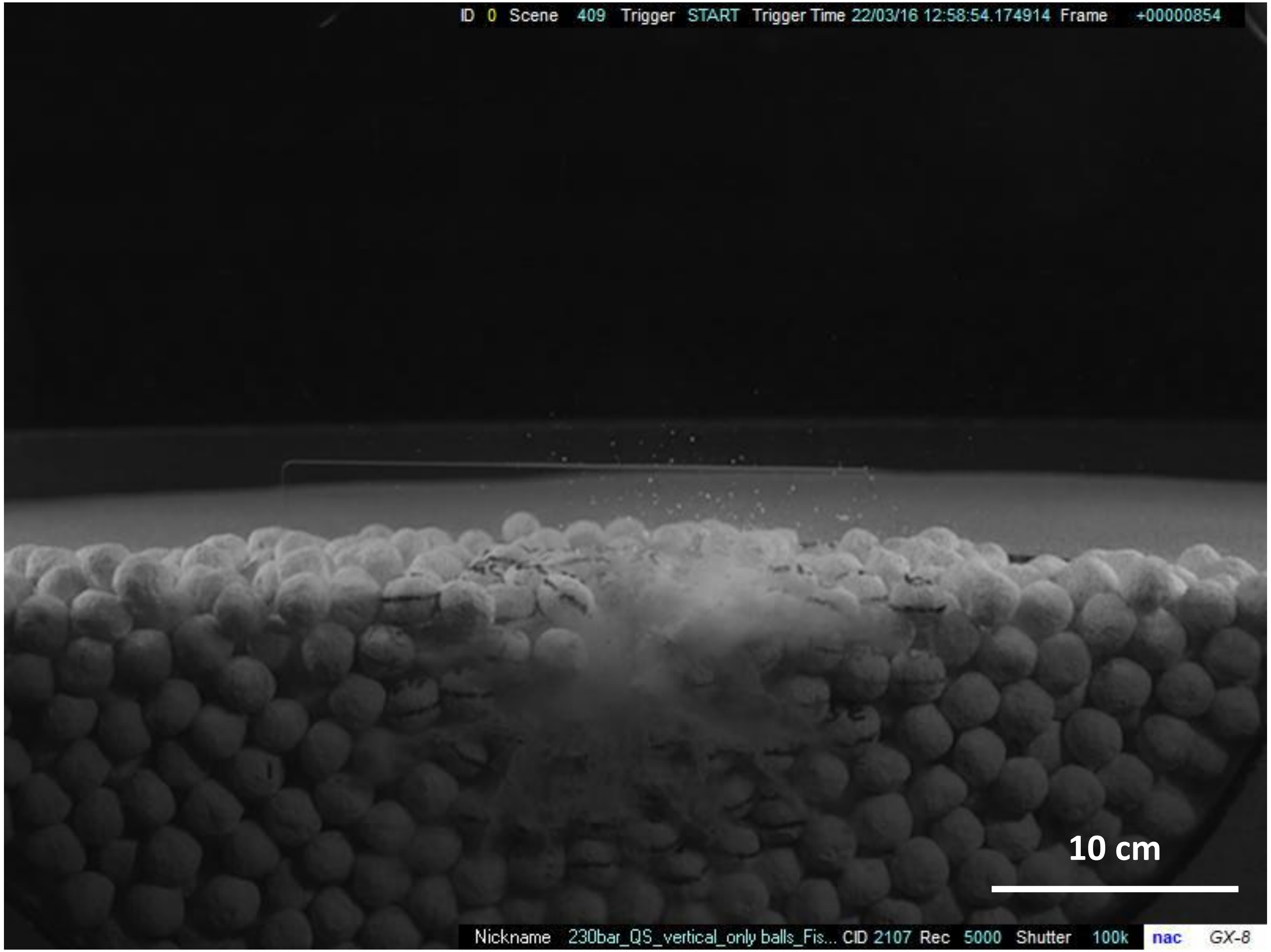
ID 0 Scene 409 Trigger START Trigger Time 22/03/16 12:58:54.174914 Frame +00000854
10 cm
Nickname 230bar_QS_vertical_only balls_Fis... CID 2107 Rec 5000 Shutter 100k nac GX-8

"Exp 7".

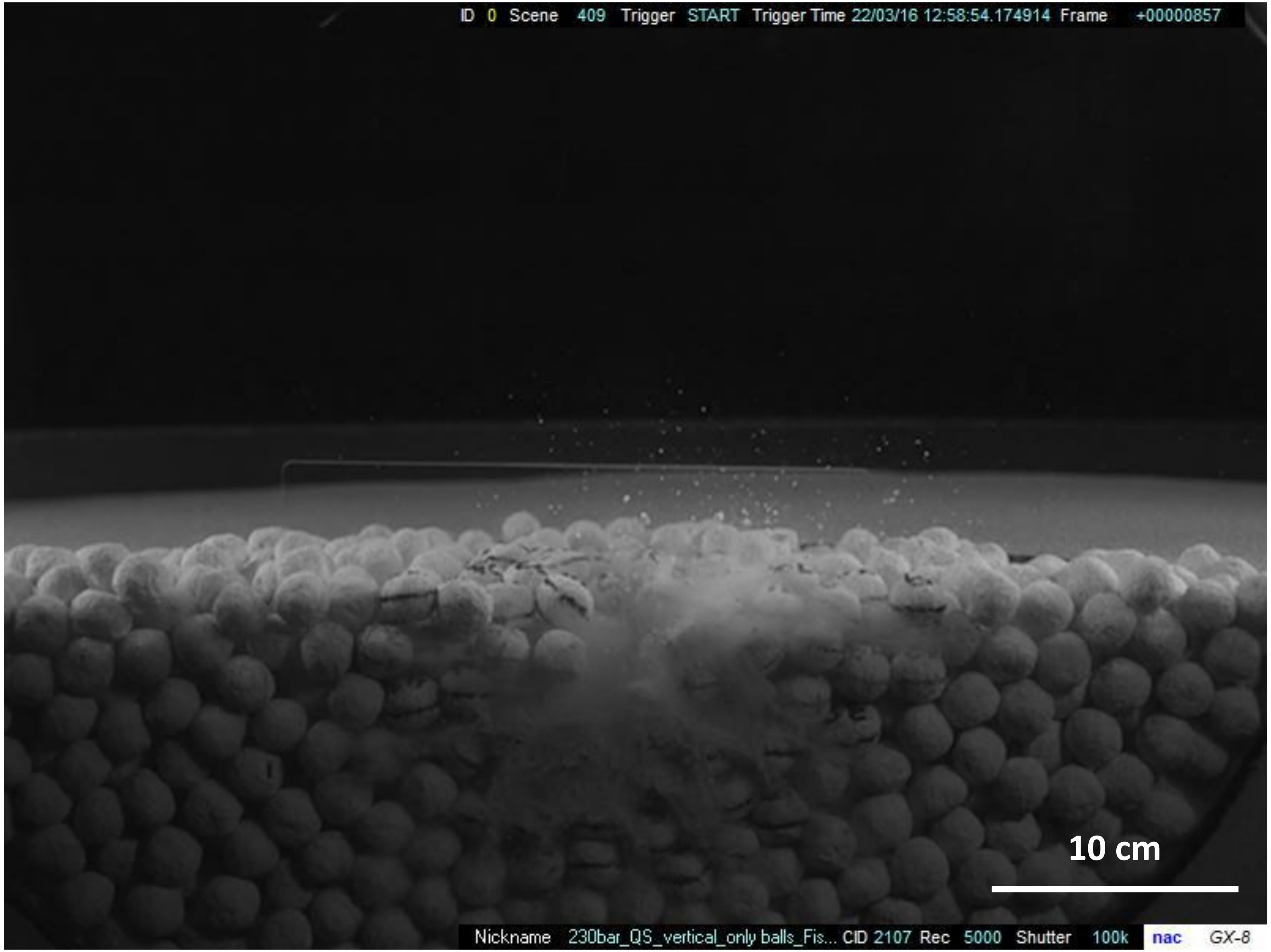
ID 0 Scene 409 Trigger START Trigger Time 22/03/16 12:58:54.174914 Frame +00000857
10 cm
Nickname 230bar_QS_vertical_only balls_Fis... CID 2107 Rec 5000 Shutter 100k nac GX-8

“Exp 7”.

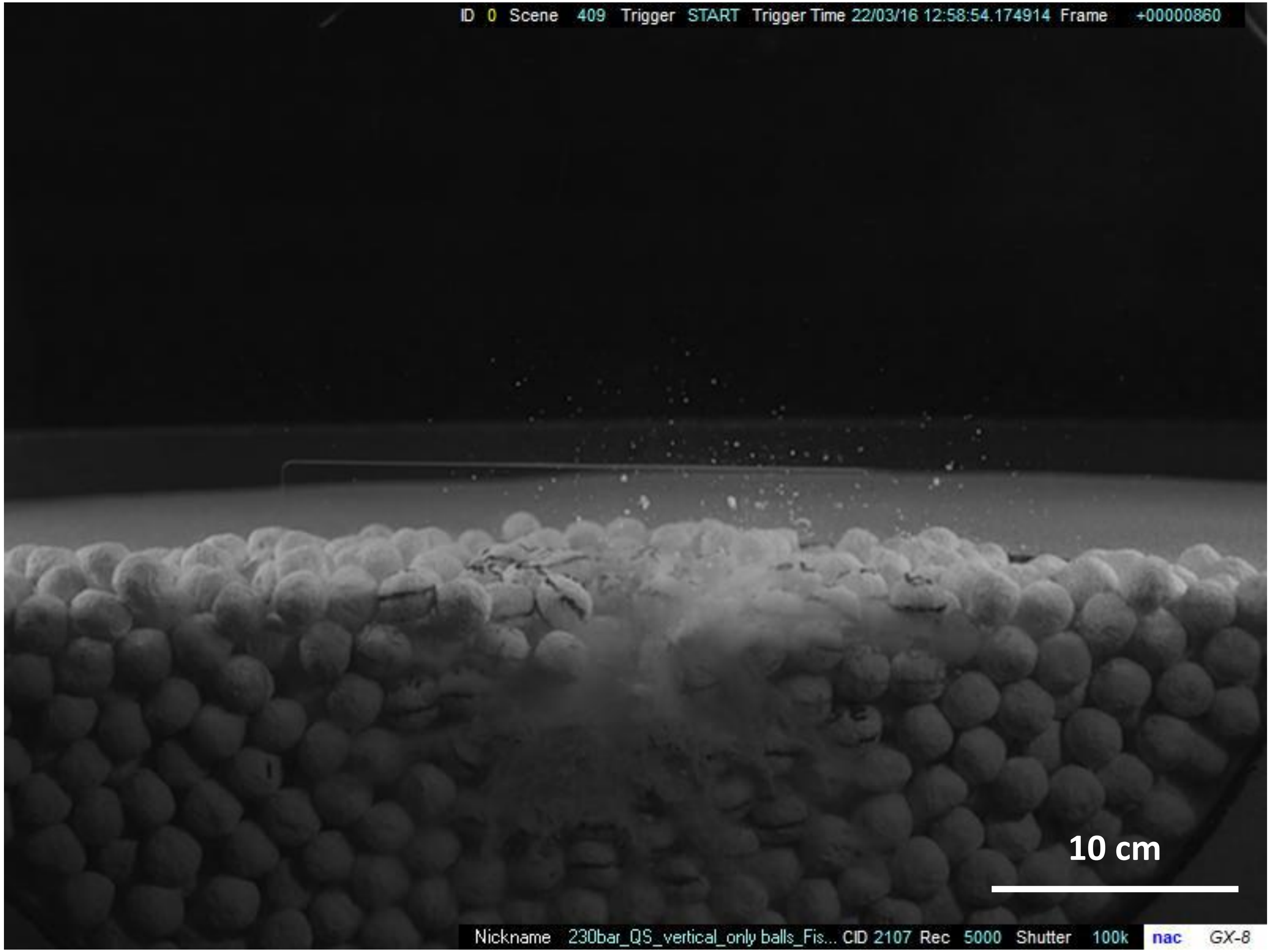
ID 0 Scene 409 Trigger START Trigger Time 22/03/16 12:58:54.174914 Frame +00000860
10 cm
Nickname 230bar_QS_vertical_only balls_Fis... CID 2107 Rec 5000 Shutter 100k nac GX-8

"Exp 7".

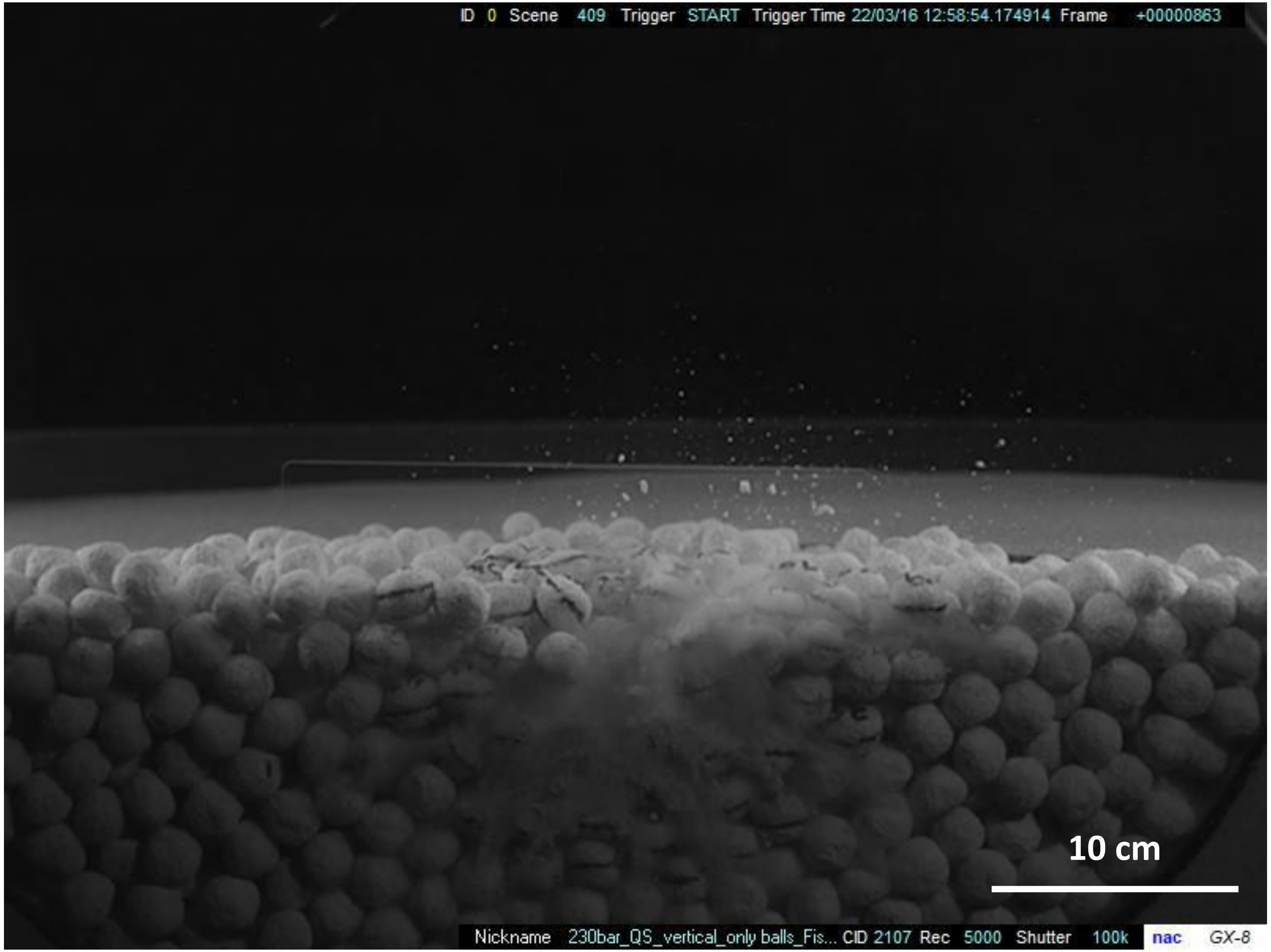
ID 0 Scene 409 Trigger START Trigger Time 22/03/16 12:58:54.174914 Frame +00000863
10 cm
Nickname 230bar_QS_vertical_only balls_Fis... CID 2107 Rec 5000 Shutter 100k nac GX-8

"Exp 7".

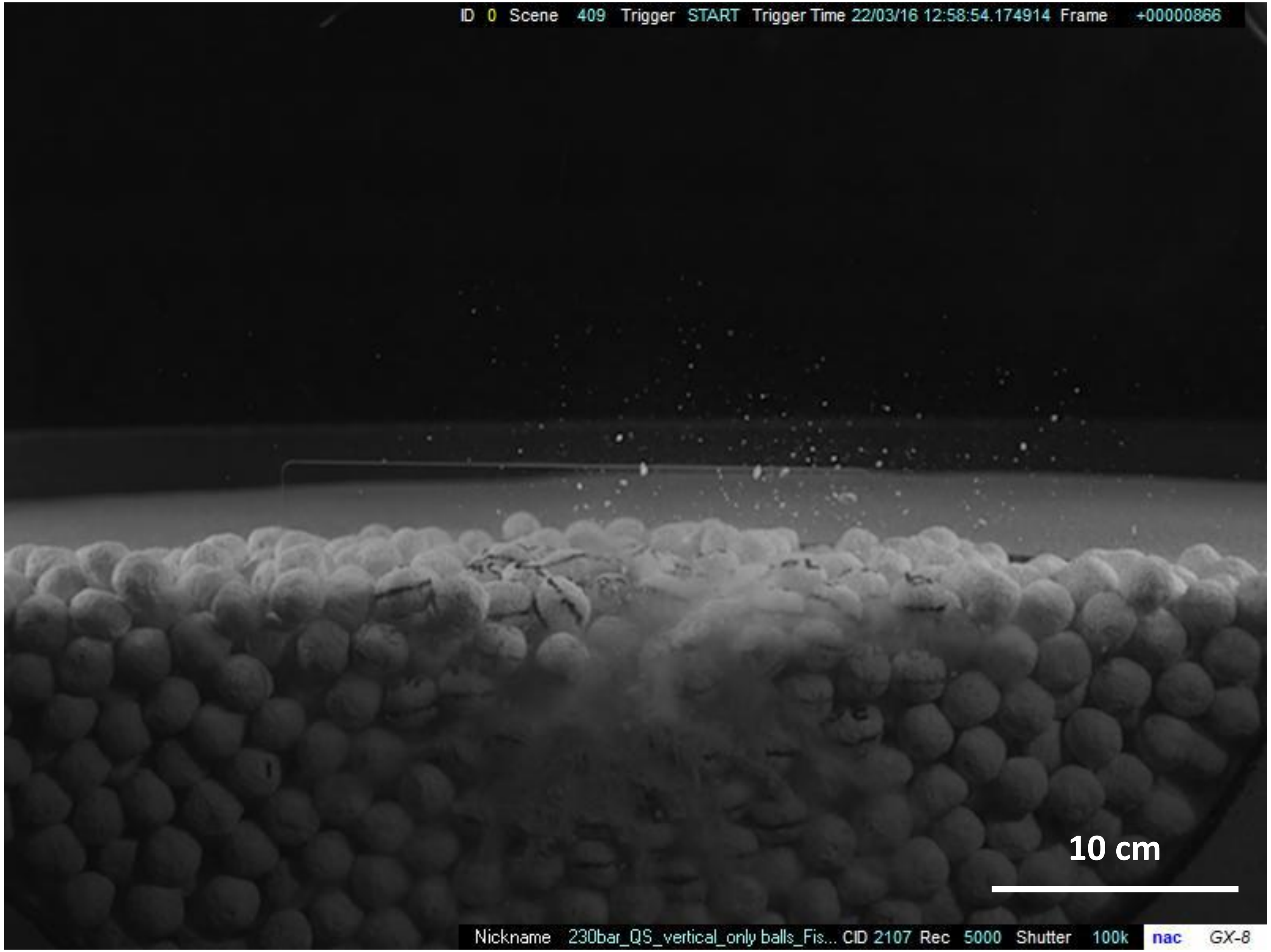
ID 0 Scene 409 Trigger START Trigger Time 22/03/16 12:58:54.174914 Frame +00000866
10 cm
Nickname 230bar_QS_vertical_only balls_Fis... CID 2107 Rec 5000 Shutter 100k nac GX-8

“Exp 7”.

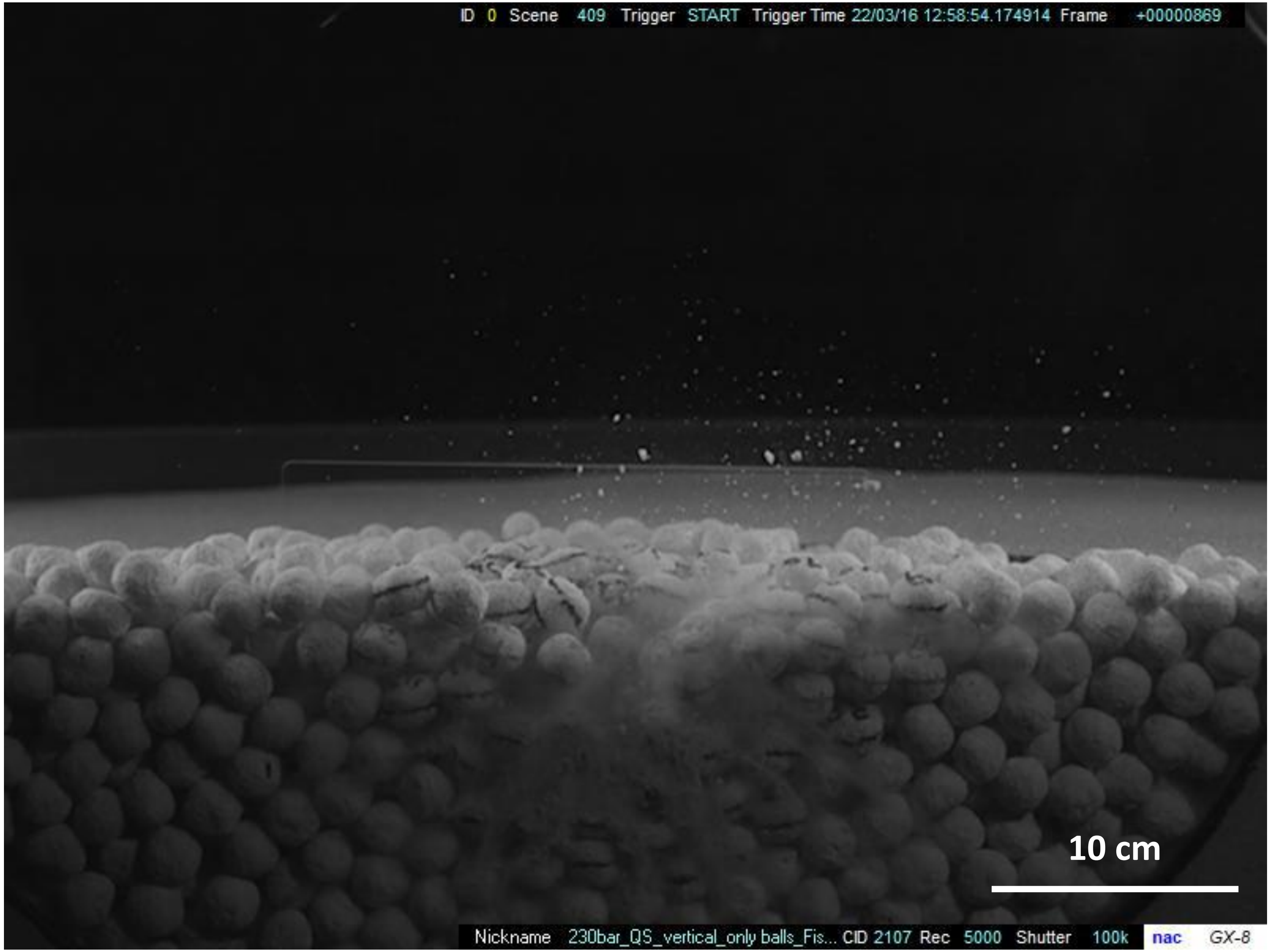
ID 0 Scene 409 Trigger START Trigger Time 22/03/16 12:58:54.174914 Frame +00000869
10 cm
Nickname 230bar_QS_vertical_only balls_Fis... CID 2107 Rec 5000 Shutter 100k nac GX-8

“Exp 7”.

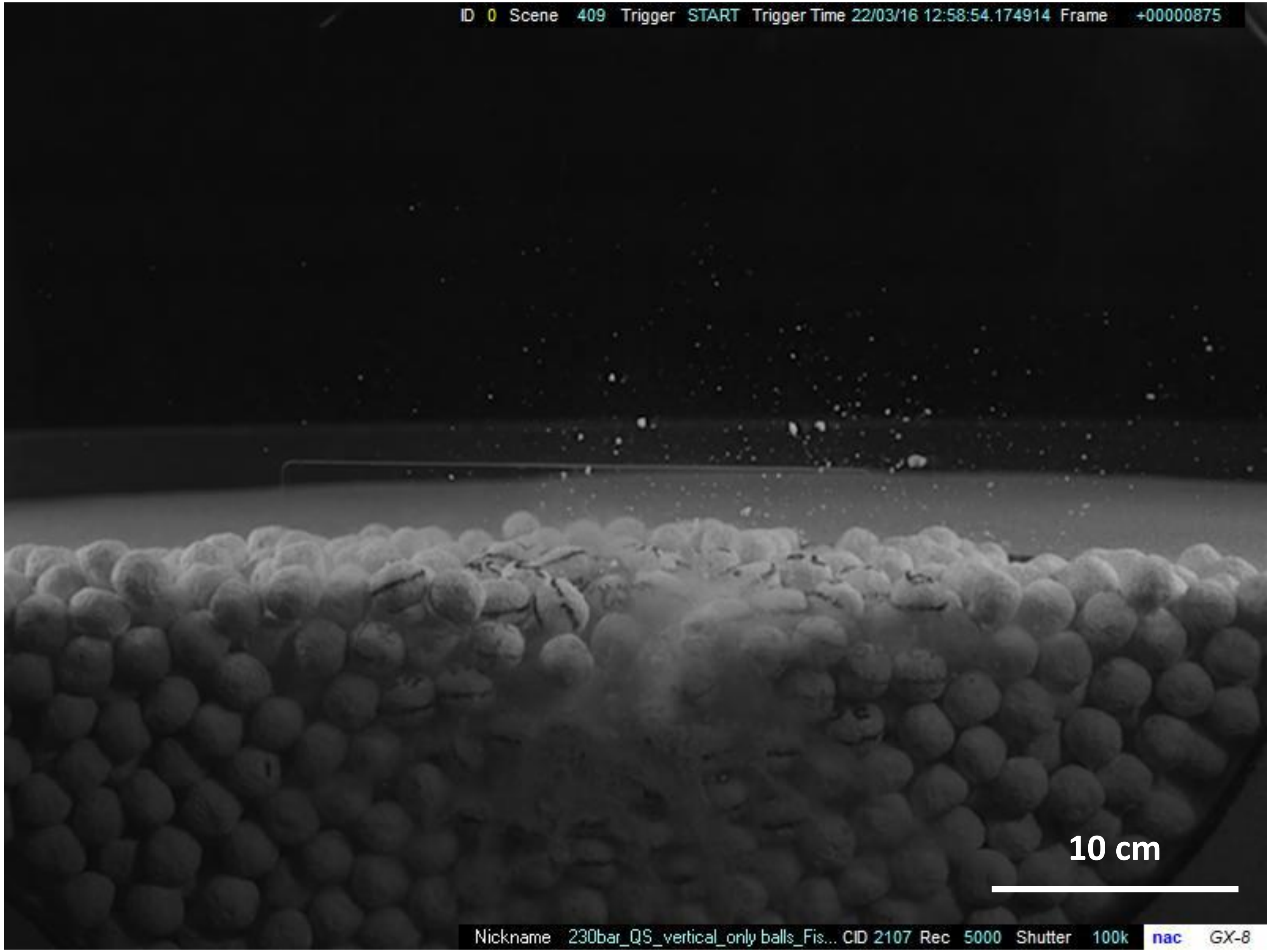
ID 0 Scene 409 Trigger START Trigger Time 22/03/16 12:58:54.174914 Frame +00000875
10 cm
Nickname 230bar_QS_vertical_only balls_Fis... CID 2107 Rec 5000 Shutter 100k nac GX-8

"Exp 7".

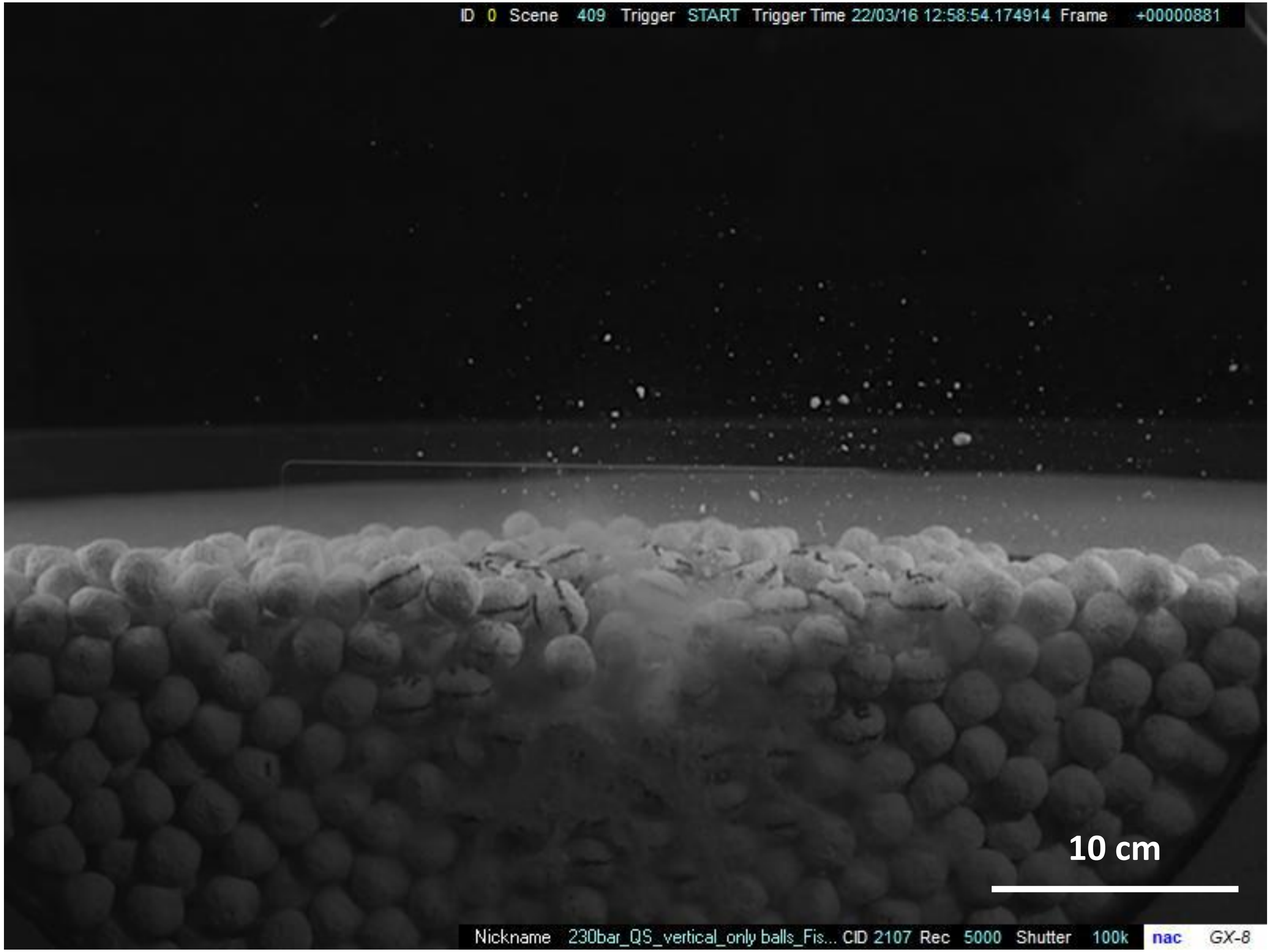
ID 0 Scene 409 Trigger START Trigger Time 22/03/16 12:58:54.174914 Frame +00000881
10 cm
Nickname 230bar_QS_vertical_only balls_Fis... CID 2107 Rec 5000 Shutter 100k nac GX-8

“Exp 7”.

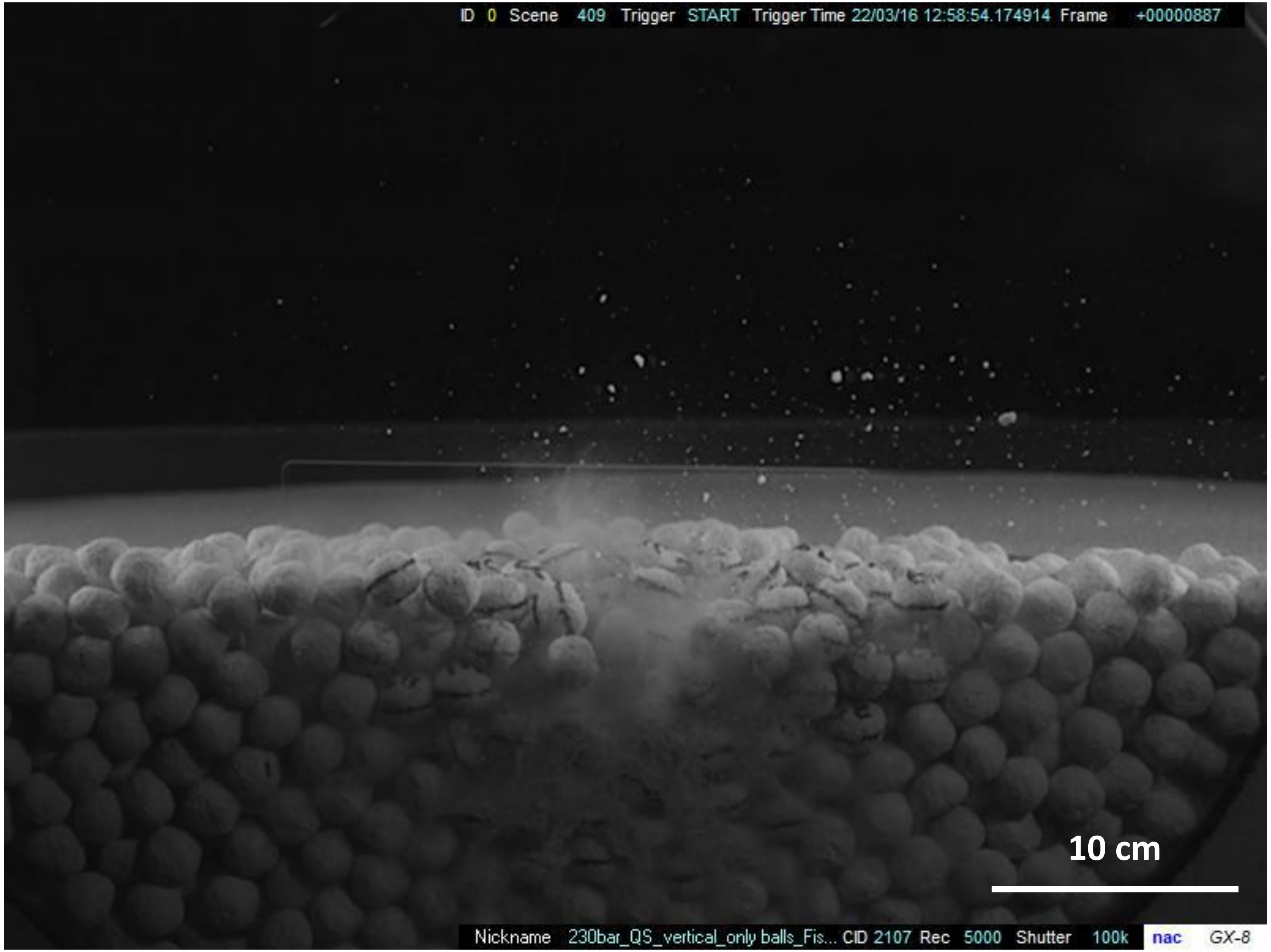
ID 0 Scene 409 Trigger START Trigger Time 22/03/16 12:58:54.174914 Frame +00000887
10 cm
Nickname 230bar_QS_vertical_only balls_Fis... CID 2107 Rec 5000 Shutter 100k nac GX-8

“Exp 7”.

“Exp 6”: Bed of two layers of projectile-size boulders covering “fast pour” sand

Projectile:

- Delrin (20 mm diameter)
- 5.7g
- 1.36g/cm^3
- Velocity: ~408 m/s

Target balls:

- Mass: 5.7 ±0.5 g
- Diameter: 2.25 ±0.16 cm
- Density: ~0.96 g/cm3
- Porosity: ~66%
- Compressive crush strength:~1 Mp

Target sand:

- Density (fast pour): ~1.6 g/cm3
- Porosity (fast pour): ~40%
- Friction angle (fast pour): 30.4 ± 1.7°

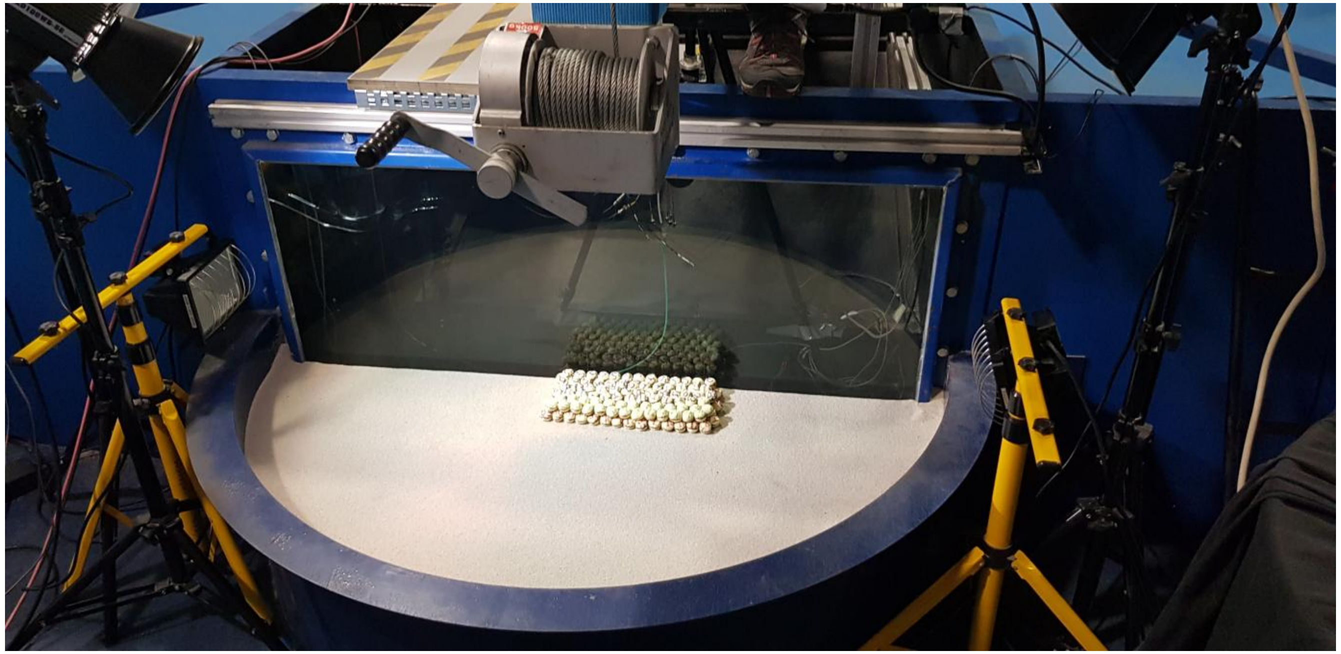

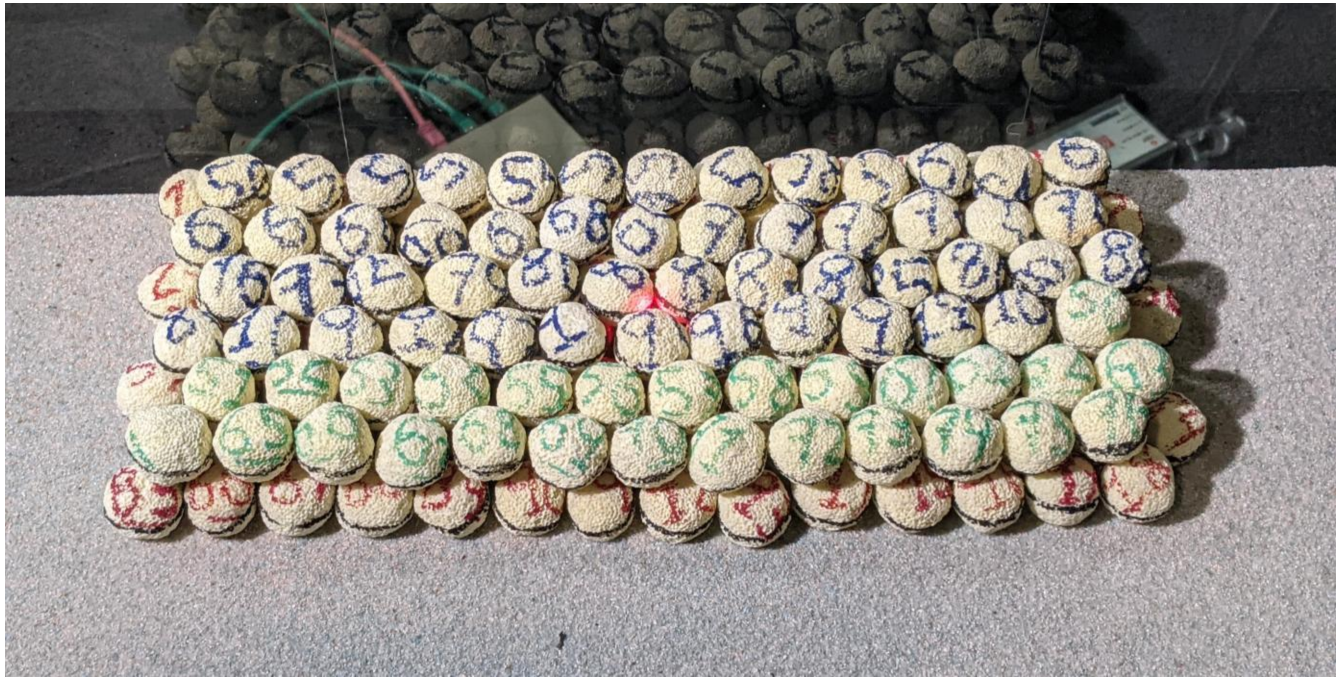

“Exp 6”.

“Exp 6”.

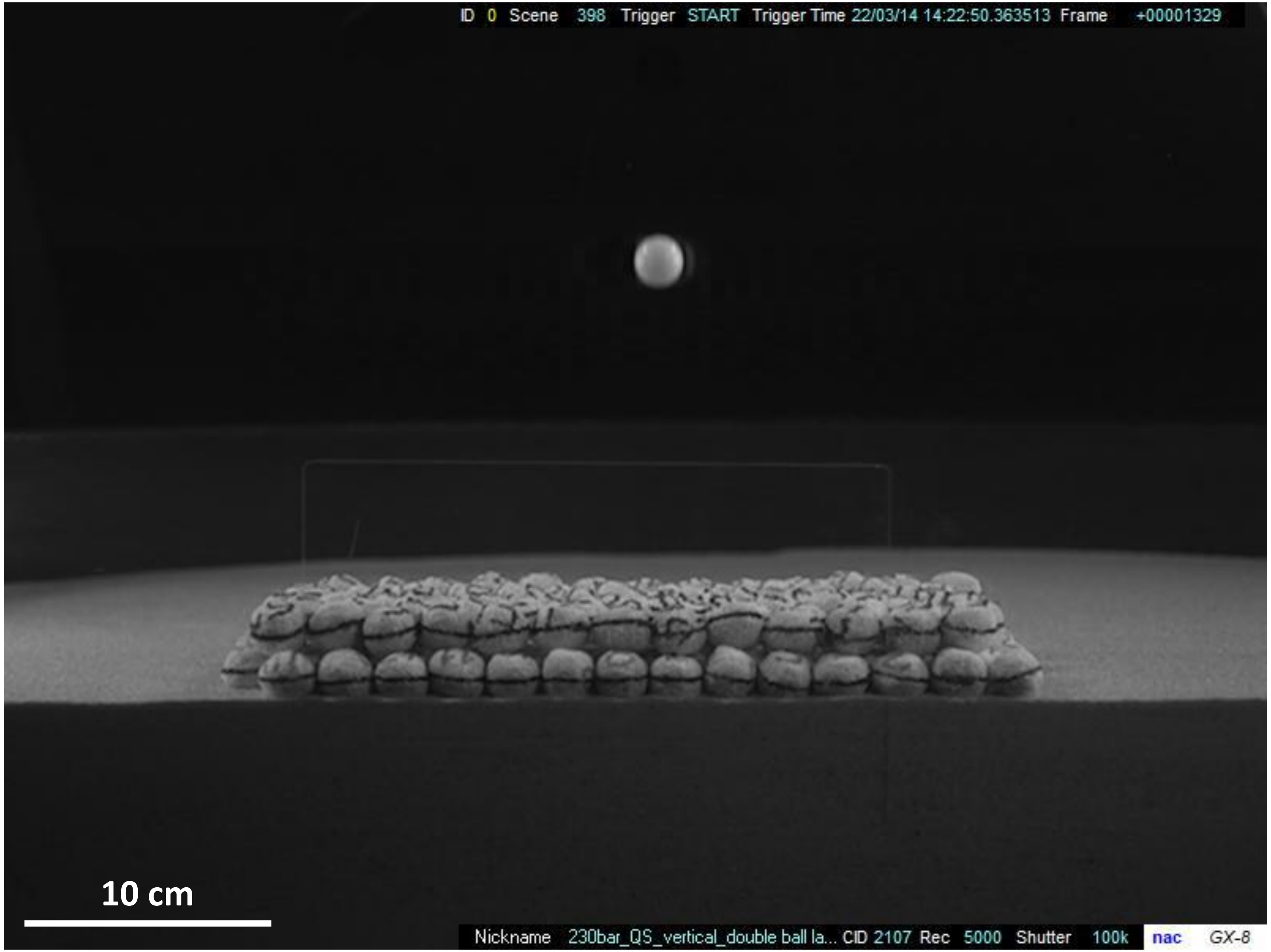
ID 0 Scene 398 Trigger START Trigger Time 22/03/14 14:22:50.363513 Frame +00001329
10 cm
Nickname 230bar_QS_vertical_double ball la... CID 2107 Rec 5000 Shutter 100k nac GX-8

“Exp 6”.

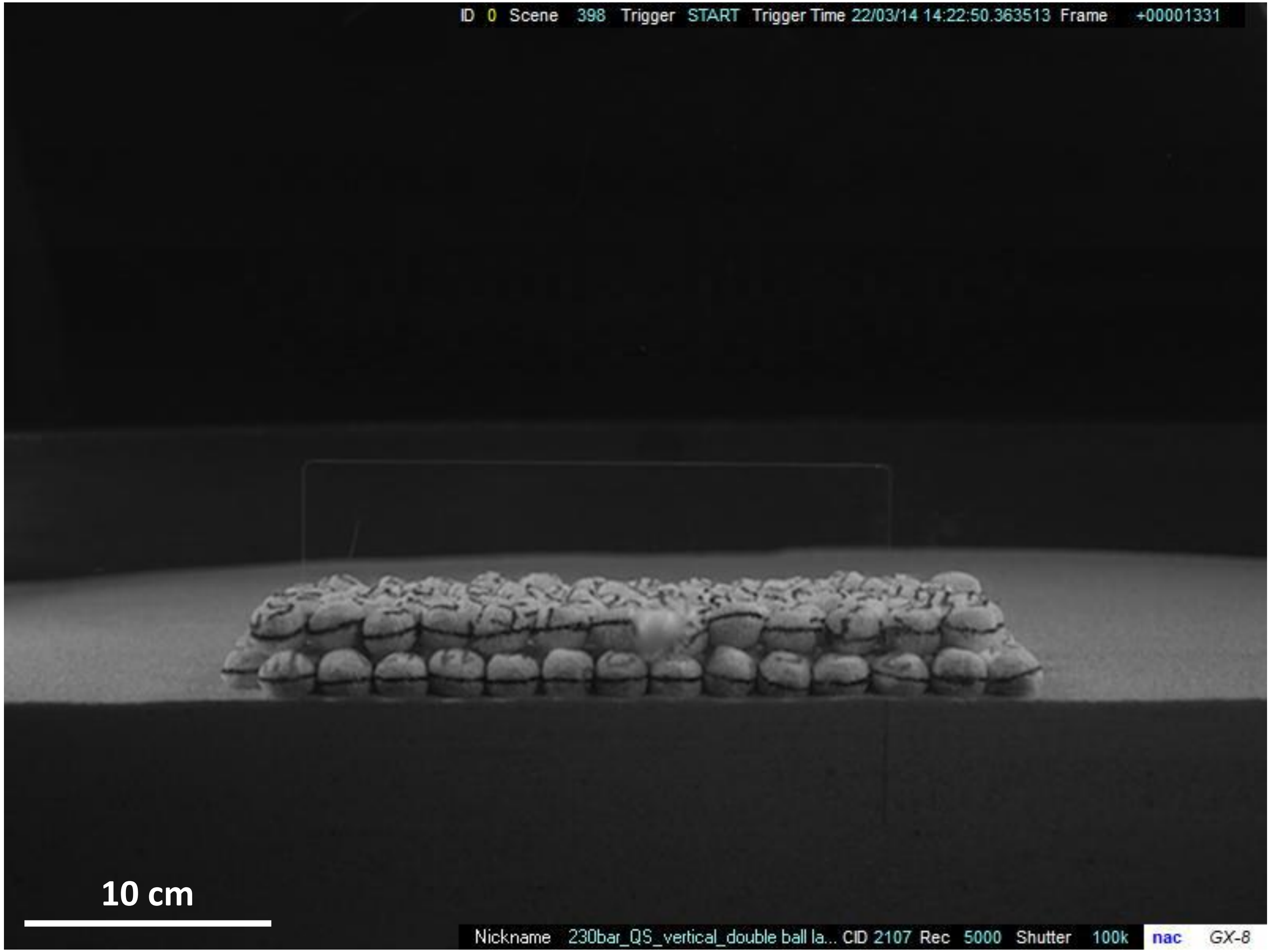
ID 0 Scene 398 Trigger START Trigger Time 22/03/14 14:22:50.363513 Frame +00001331
10 cm
Nickname 230bar_QS_vertical_double ball la... CID 2107 Rec 5000 Shutter 100k nac GX-8

"Exp 6".

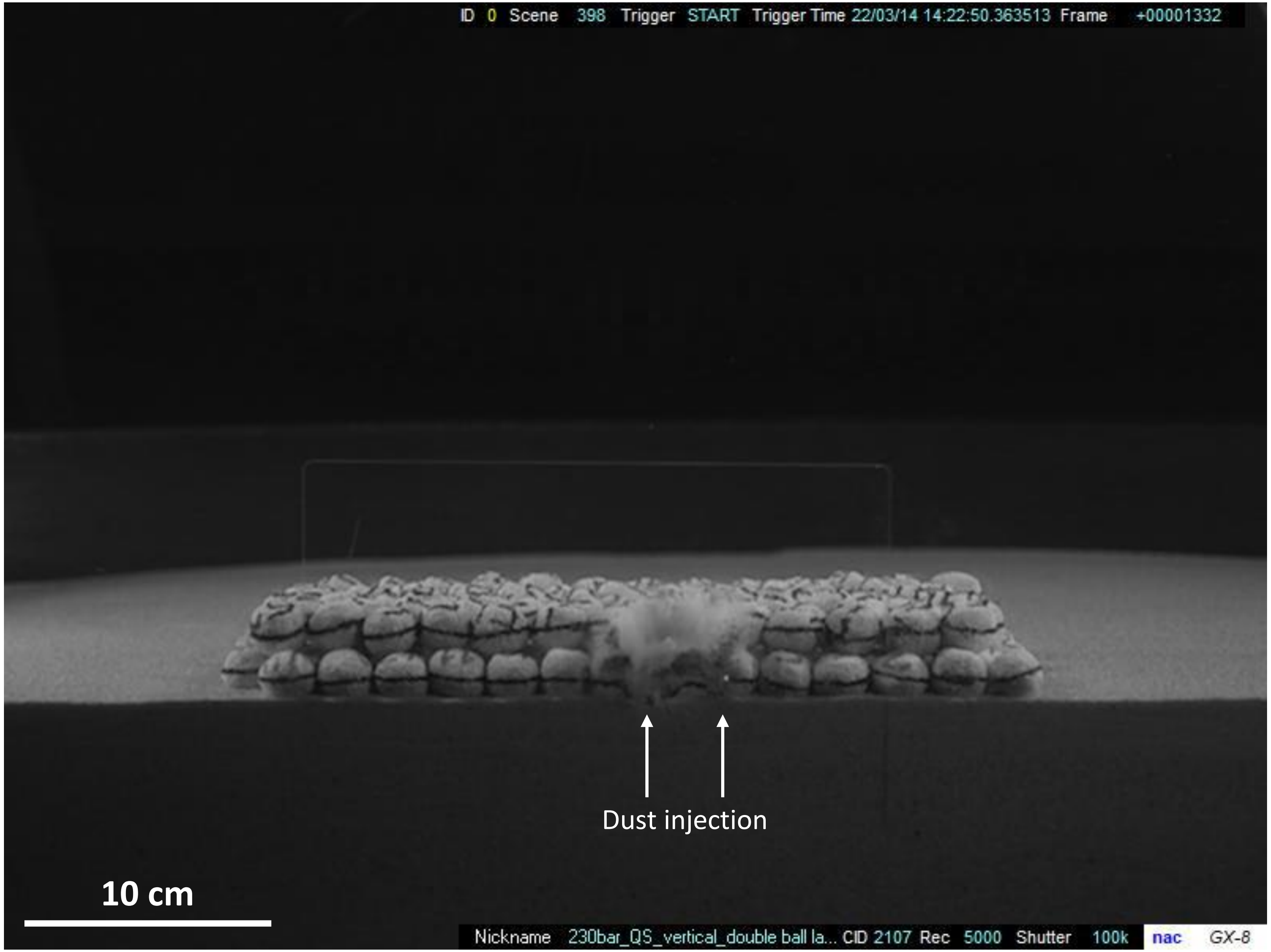
ID 0 Scene 398 Trigger START Trigger Time 22/03/14 14:22:50.363513 Frame +00001332
Dust injection
10 cm
Nickname 230bar_QS_vertical_double ball la... CID 2107 Rec 5000 Shutter 100k nac GX-8

"Exp 6".

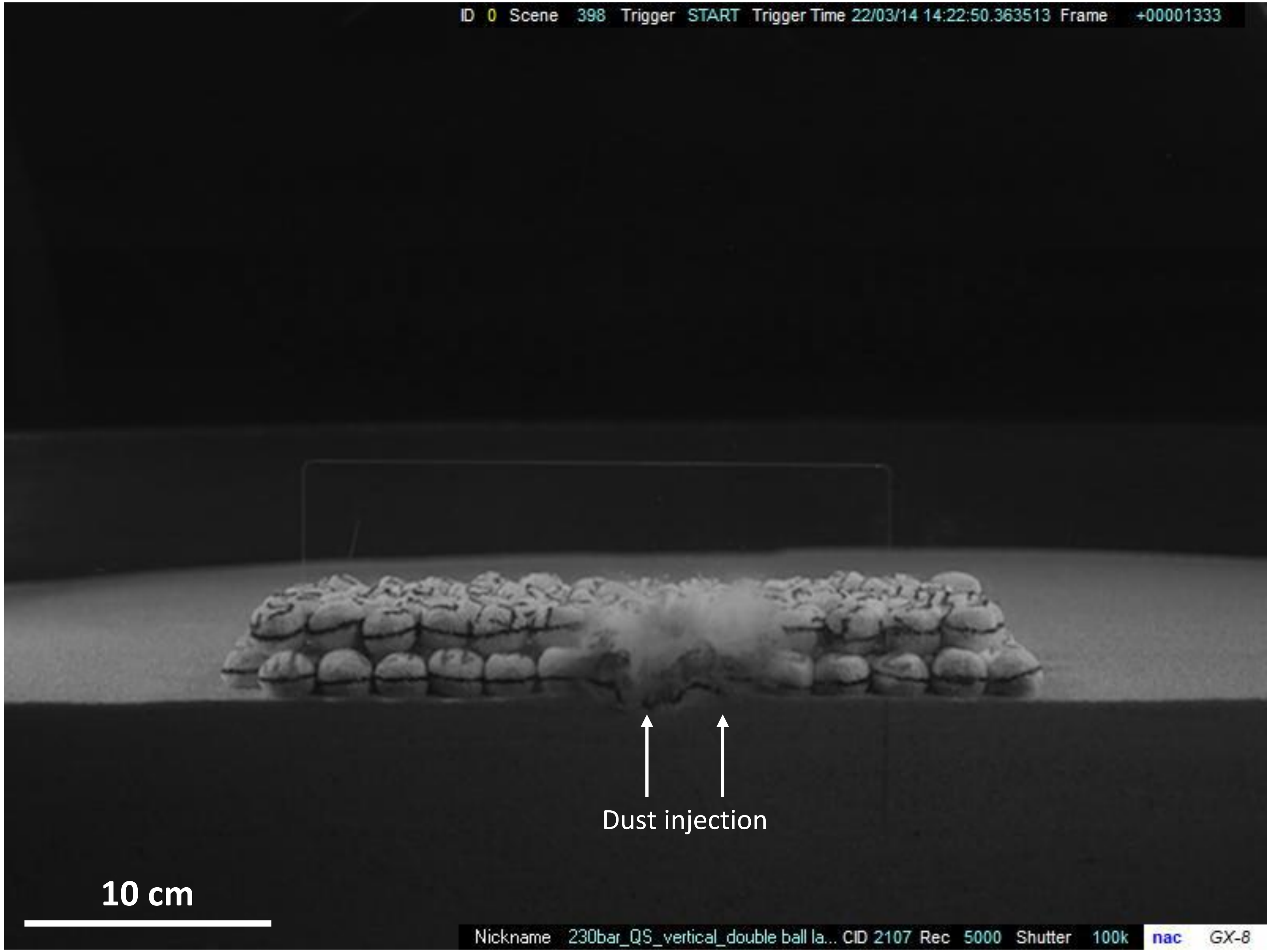
ID 0 Scene 398 Trigger START Trigger Time 22/03/14 14:22:50.363513 Frame +00001333
Dust injection
10 cm
Nickname 230bar_QS_vertical_double ball la... CID 2107 Rec 5000 Shutter 100k nac GX-8

"Exp 6".

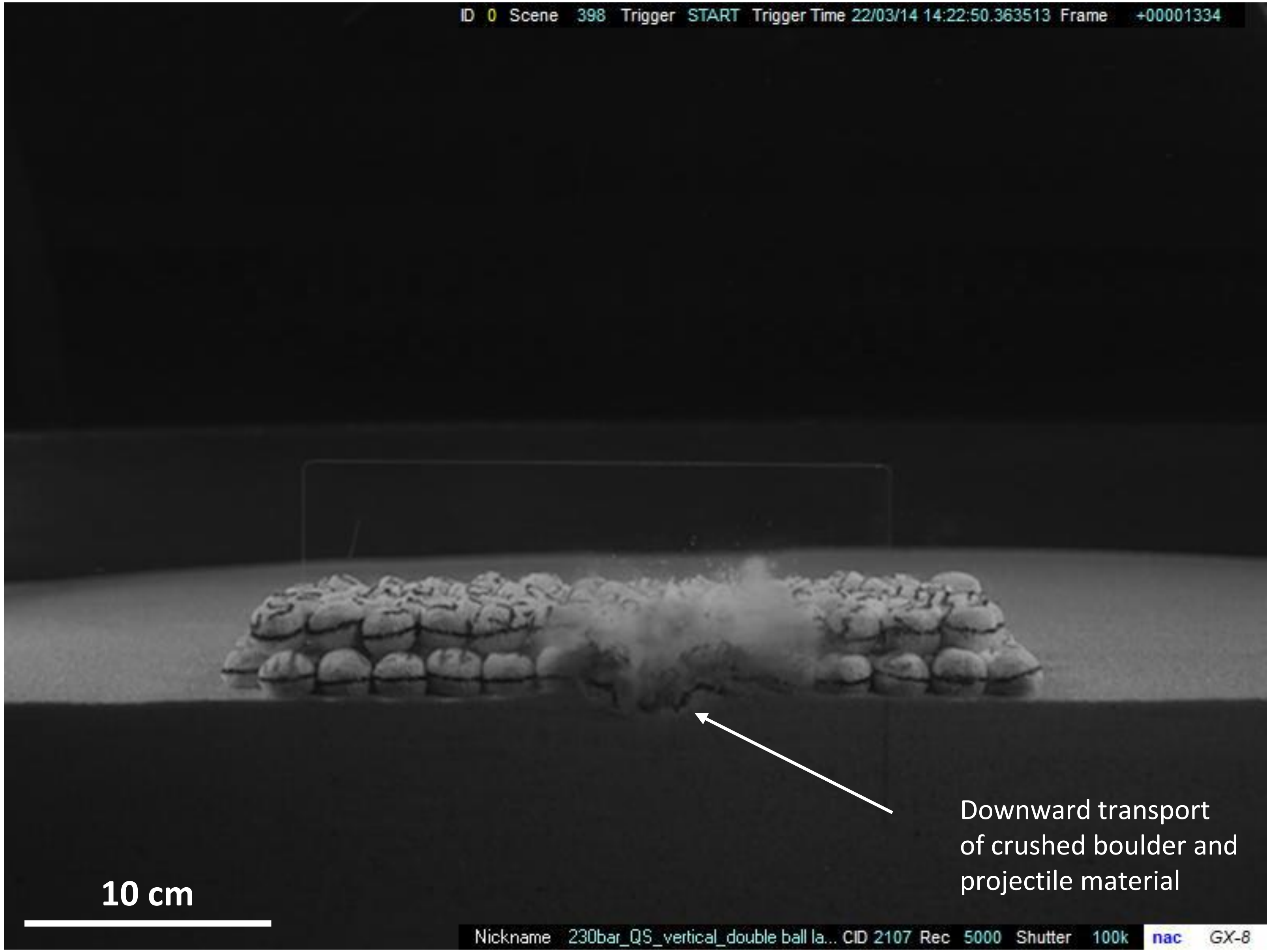
ID 0 Scene 398 Trigger START Trigger Time 22/03/14 14:22:50.363513 Frame +00001334
Downward transport
of crushed boulder and
projectile material
10 cm
Nickname 230bar_QS_vertical_double ball la... CID 2107 Rec 5000 Shutter 100k nac GX-8

“Exp 6”.

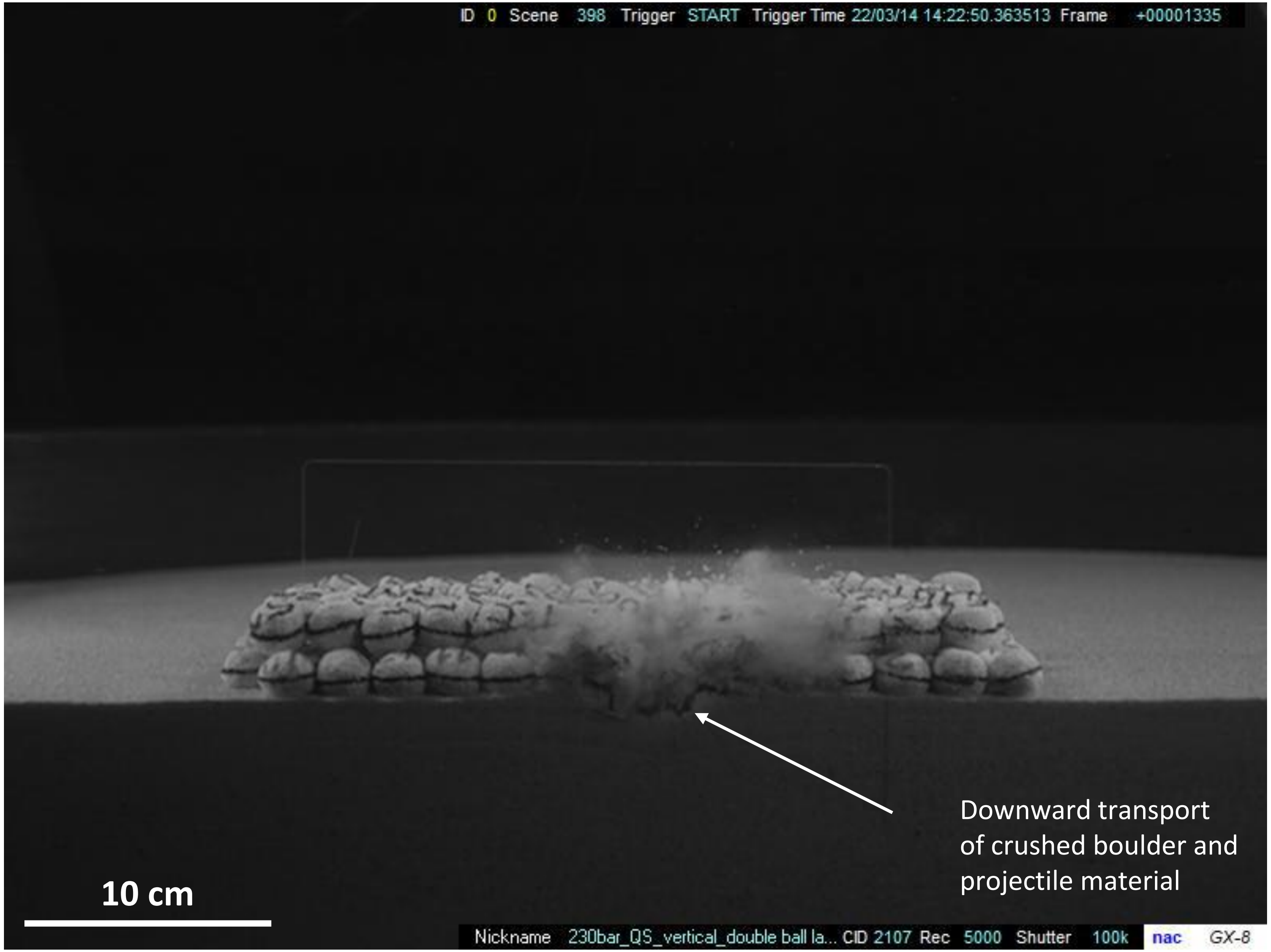
ID 0 Scene 398 Trigger START Trigger Time 22/03/14 14:22:50.363513 Frame +00001335
Downward transport of crushed boulder and projectile material
10 cm
Nickname 230bar_QS_vertical_double ball la... CID 2107 Rec 5000 Shutter 100k nac GX-8

“Exp 6”.

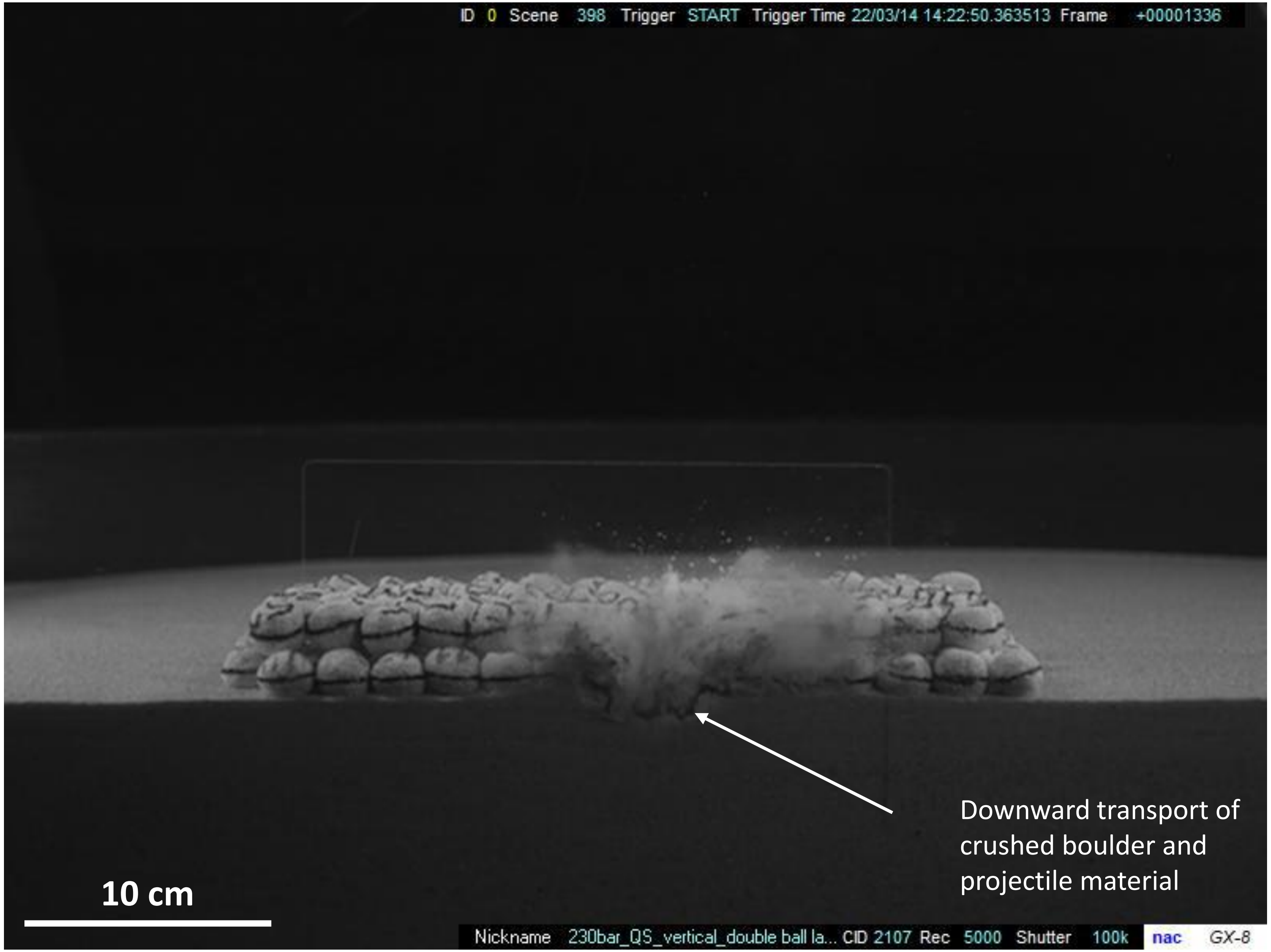
ID 0 Scene 398 Trigger START Trigger Time 22/03/14 14:22:50.363513 Frame +00001336
Downward transport of crushed boulder and projectile material
10 cm
Nickname 230bar_QS_vertical_double ball la... CID 2107 Rec 5000 Shutter 100k nac GX-8

“Exp 6”.

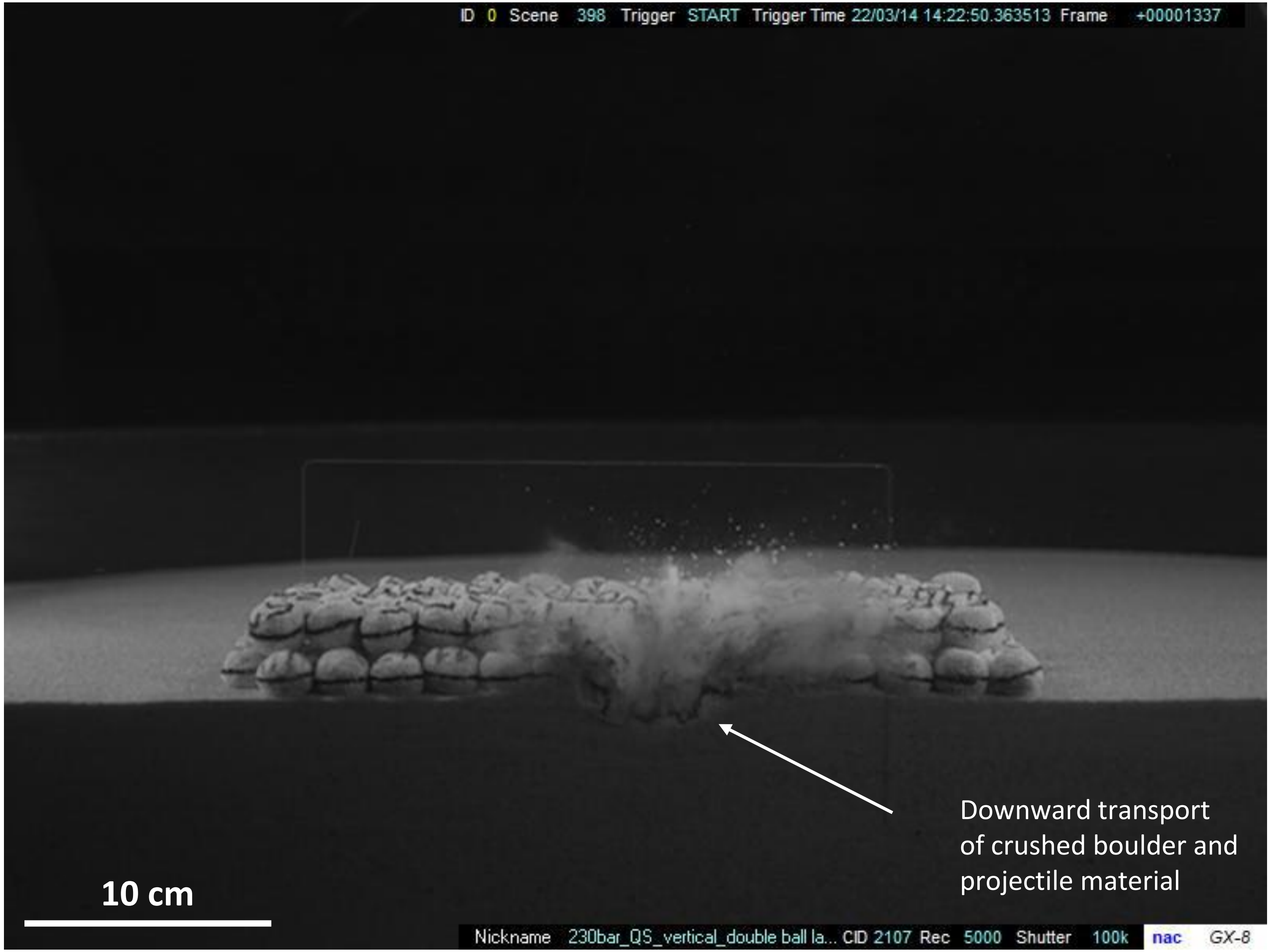
ID 0 Scene 398 Trigger START Trigger Time 22/03/14 14:22:50.363513 Frame +00001337
Downward transport of crushed boulder and projectile material
10 cm
Nickname 230bar_QS_vertical_double ball la... CID 2107 Rec 5000 Shutter 100k nac GX-8

“Exp 6”.

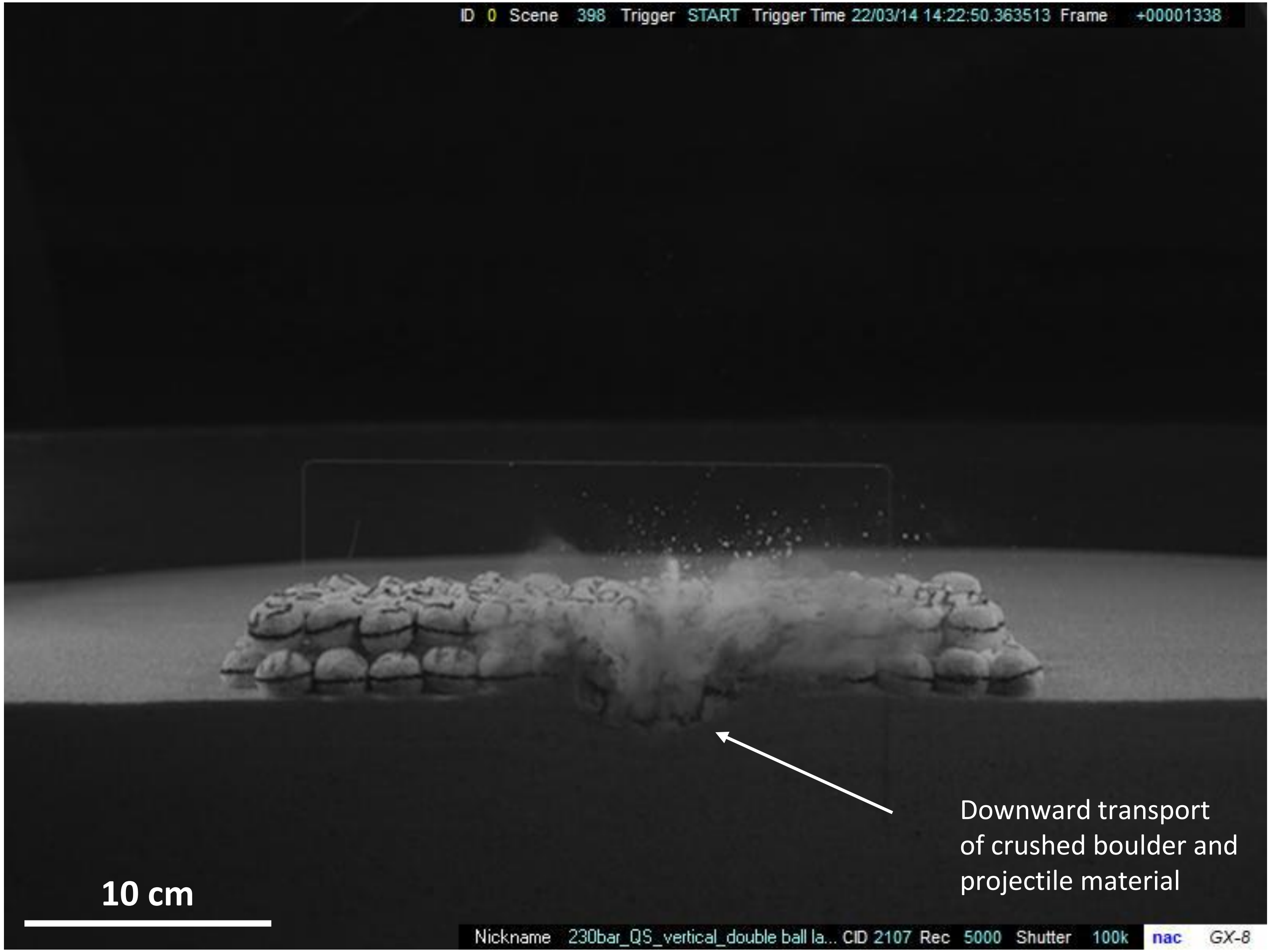
ID 0 Scene 398 Trigger START Trigger Time 22/03/14 14:22:50.363513 Frame +00001338
Downward transport
of crushed boulder and
projectile material
10 cm
Nickname 230bar_QS_vertical_double ball la... CID 2107 Rec 5000 Shutter 100k nac GX-8

“Exp 6”.

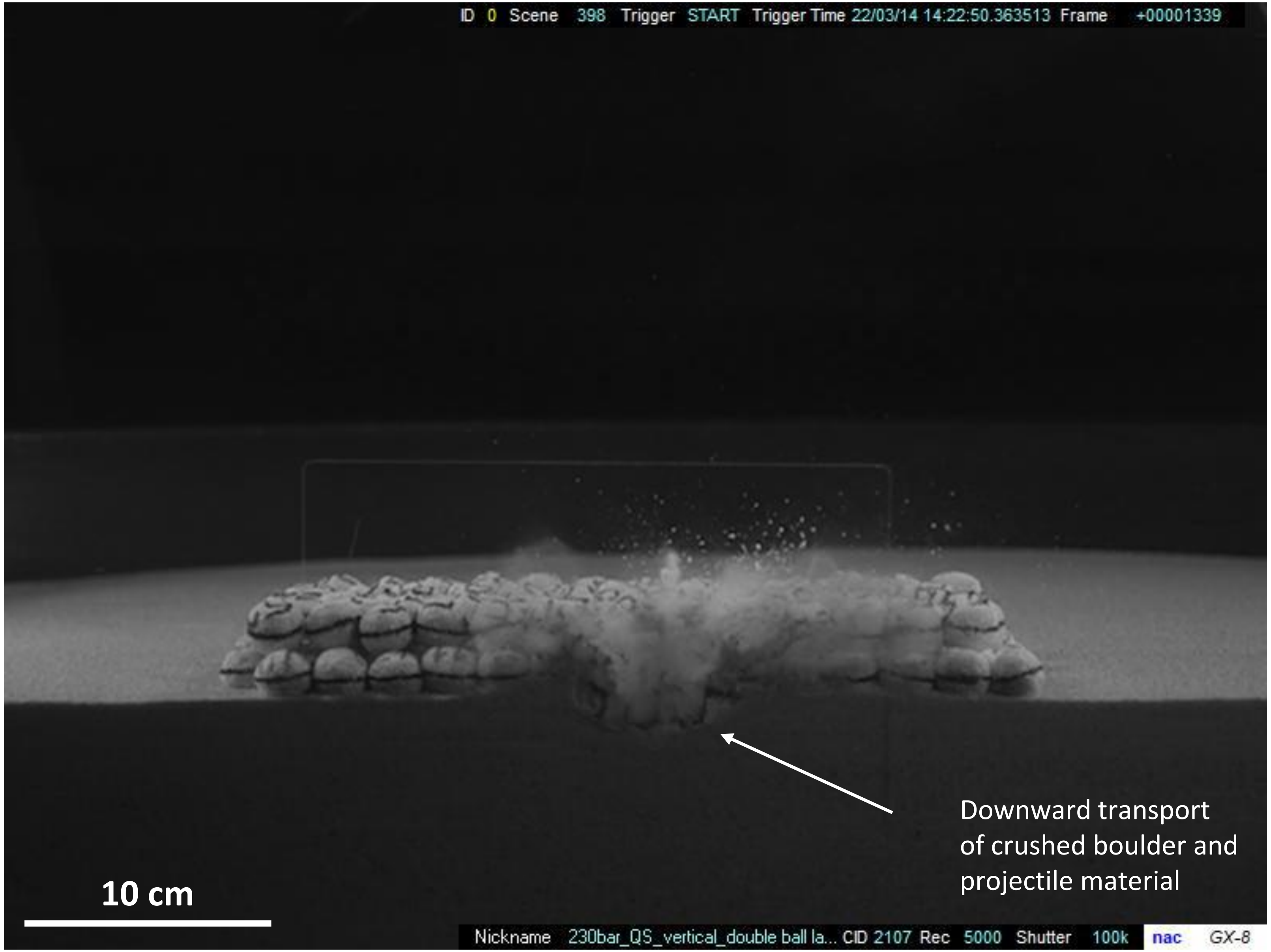
ID 0 Scene 398 Trigger START Trigger Time 22/03/14 14:22:50.363513 Frame +00001339
Downward transport of crushed boulder and projectile material
10 cm
Nickname 230bar_QS_vertical_double ball la... CID 2107 Rec 5000 Shutter 100k nac GX-8

“Exp 6”.

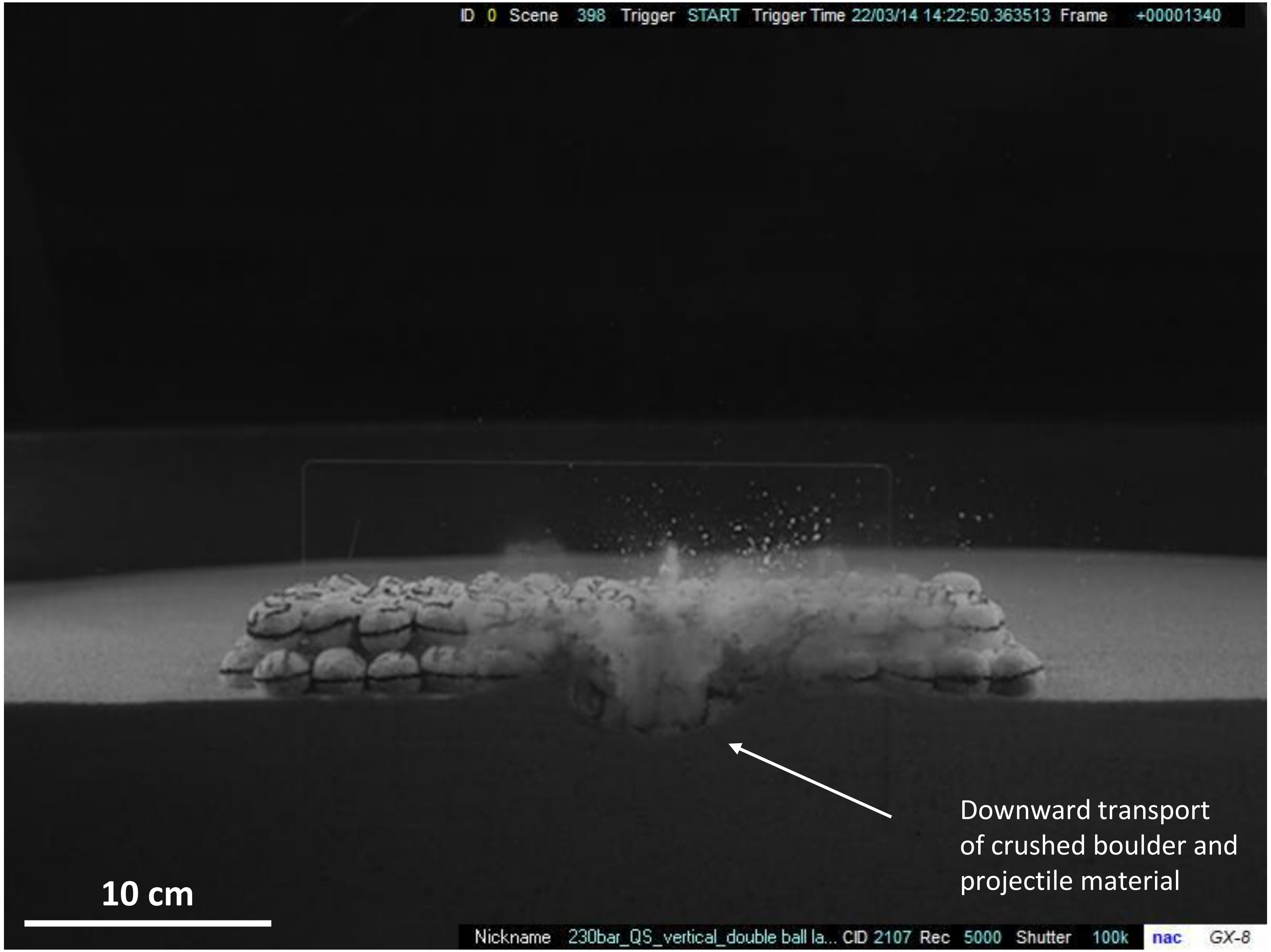
ID 0 Scene 398 Trigger START Trigger Time 22/03/14 14:22:50.363513 Frame +00001340
Downward transport of crushed boulder and projectile material
10 cm
Nickname 230bar_QS_vertical_double ball la... CID 2107 Rec 5000 Shutter 100k nac GX-8

“Exp 6”.

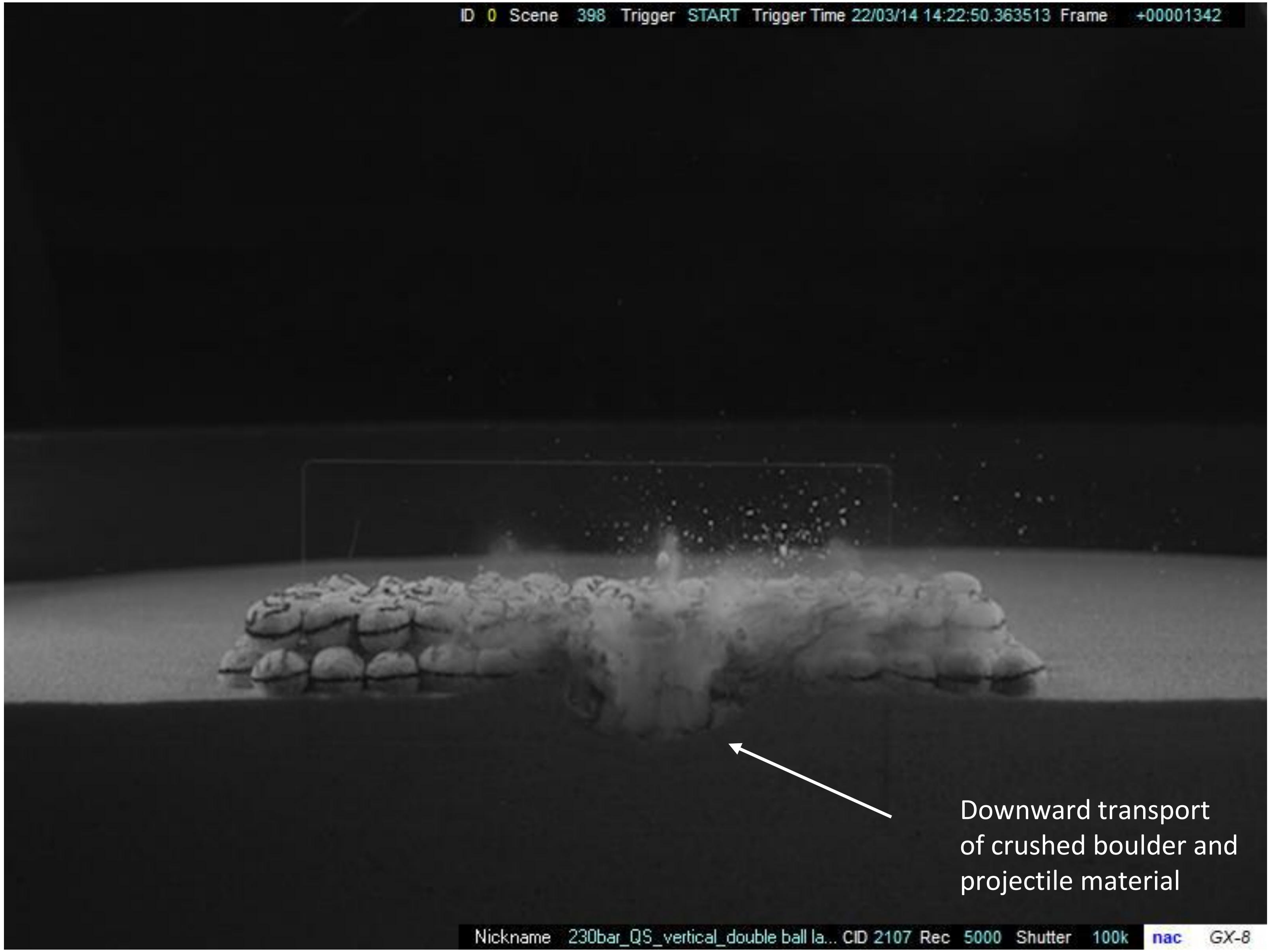
ID 0 Scene 398 Trigger START Trigger Time 22/03/14 14:22:50.363513 Frame +00001342
Downward transport of crushed boulder and projectile material
Nickname 230bar_QS_vertical_double ball la... CID 2107 Rec 5000 Shutter 100k nac GX-8

“Exp 6”.

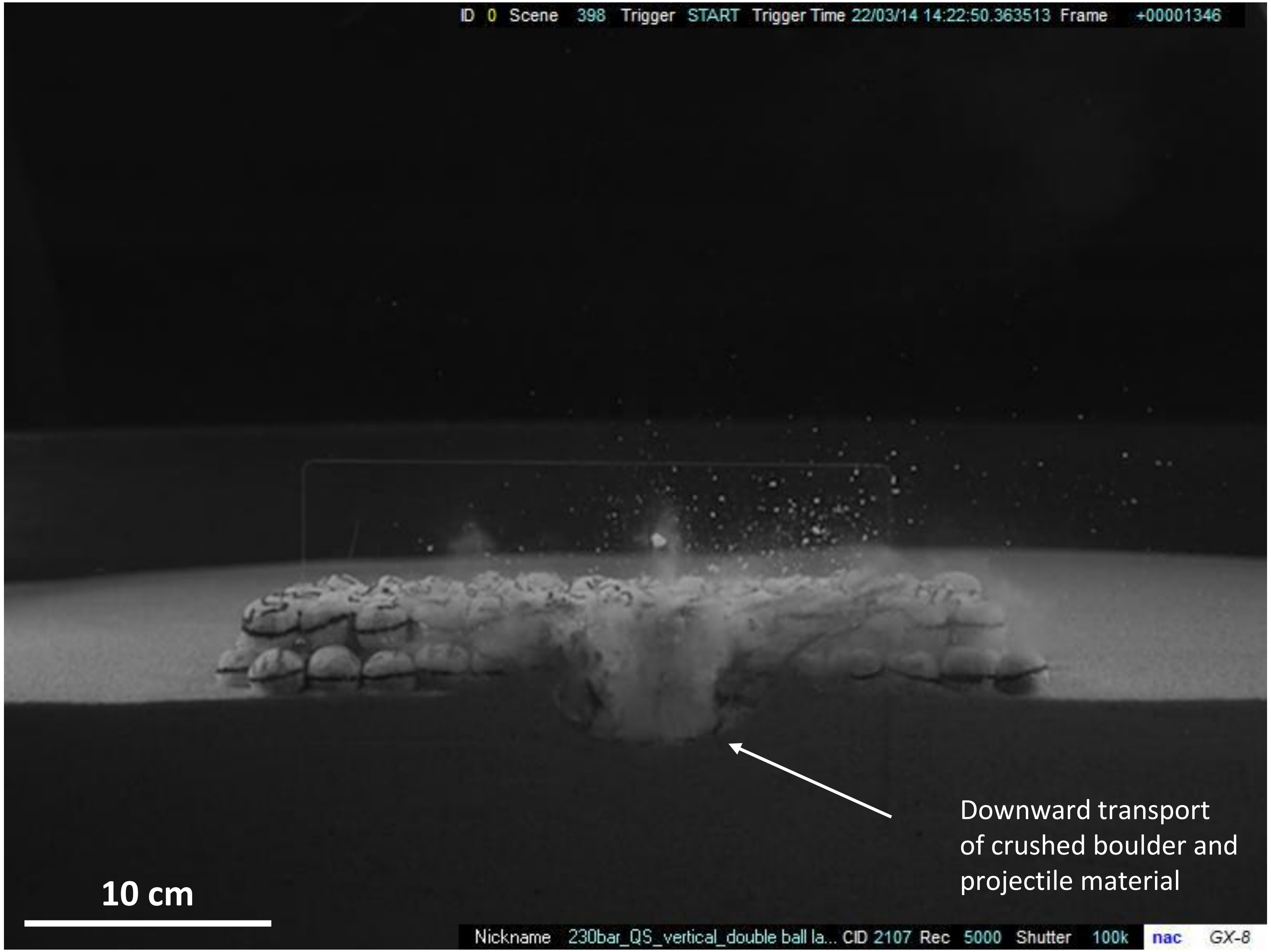
ID 0 Scene 398 Trigger START Trigger Time 22/03/14 14:22:50.363513 Frame +00001346
Downward transport of crushed boulder and projectile material
10 cm
Nickname 230bar_QS_vertical_double ball la... CID 2107 Rec 5000 Shutter 100k nac GX-8

“Exp 6”.

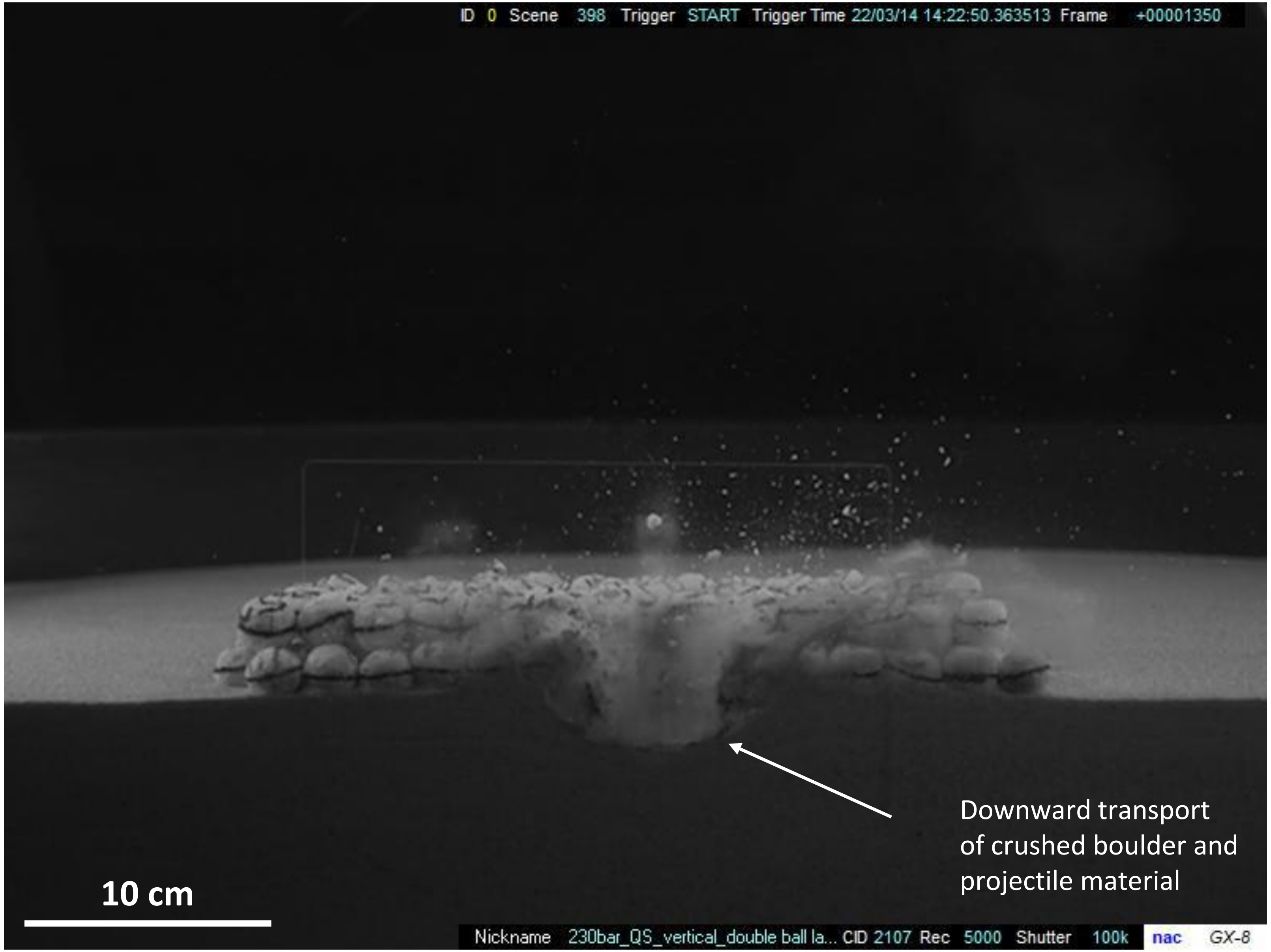
ID 0 Scene 398 Trigger START Trigger Time 22/03/14 14:22:50.363513 Frame +00001350
Downward transport
of crushed boulder and
projectile material
10 cm
Nickname 230bar_QS_vertical_double ball la... CID 2107 Rec 5000 Shutter 100k nac GX-8

"Exp 6".

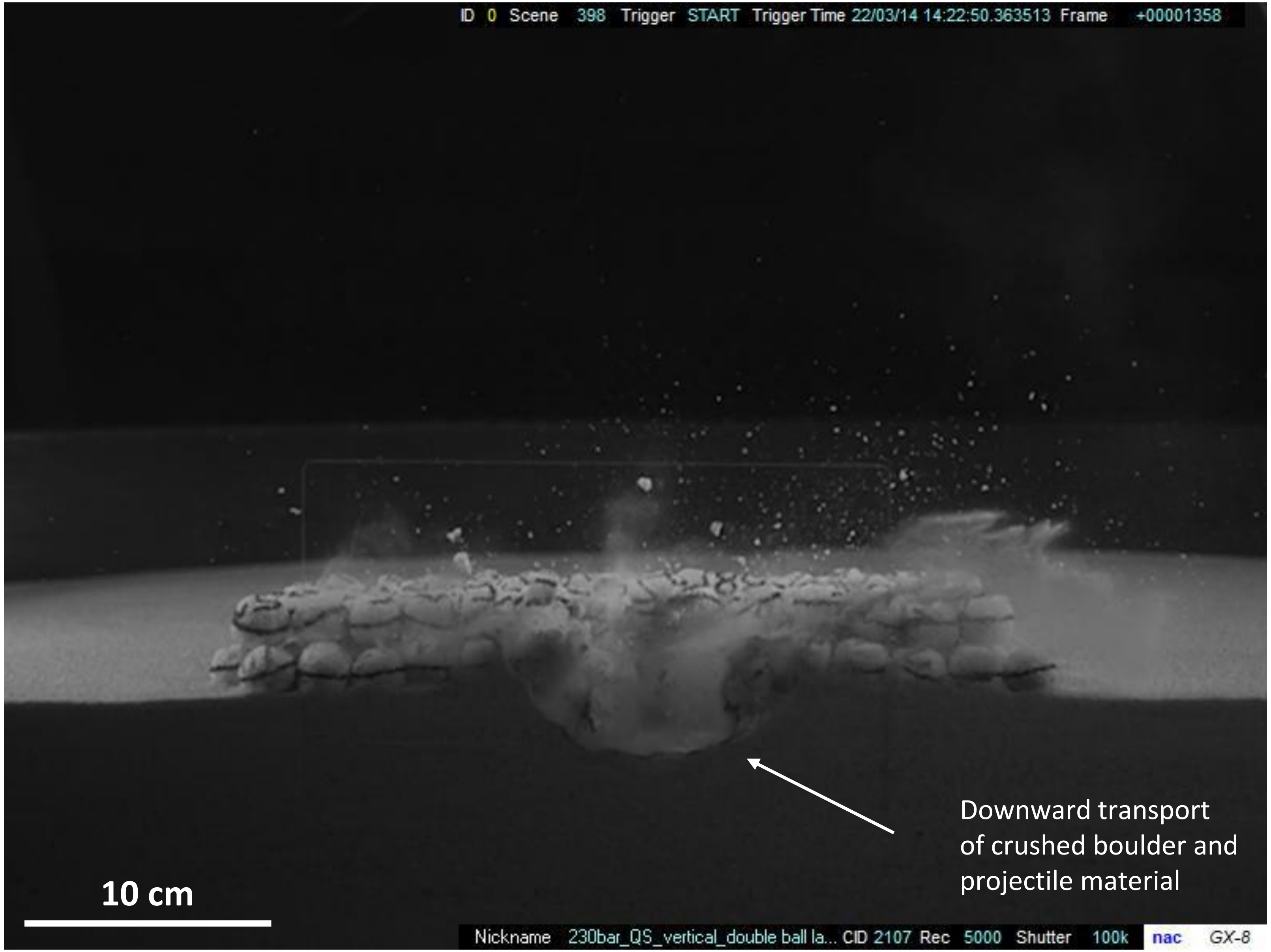
ID 0 Scene 398 Trigger START Trigger Time 22/03/14 14:22:50.363513 Frame +00001358
Downward transport
of crushed boulder and
projectile material
10 cm
Nickname 230bar_QS_vertical_double ball la... CID 2107 Rec 5000 Shutter 100k
nac GX-8

“Exp 6”.

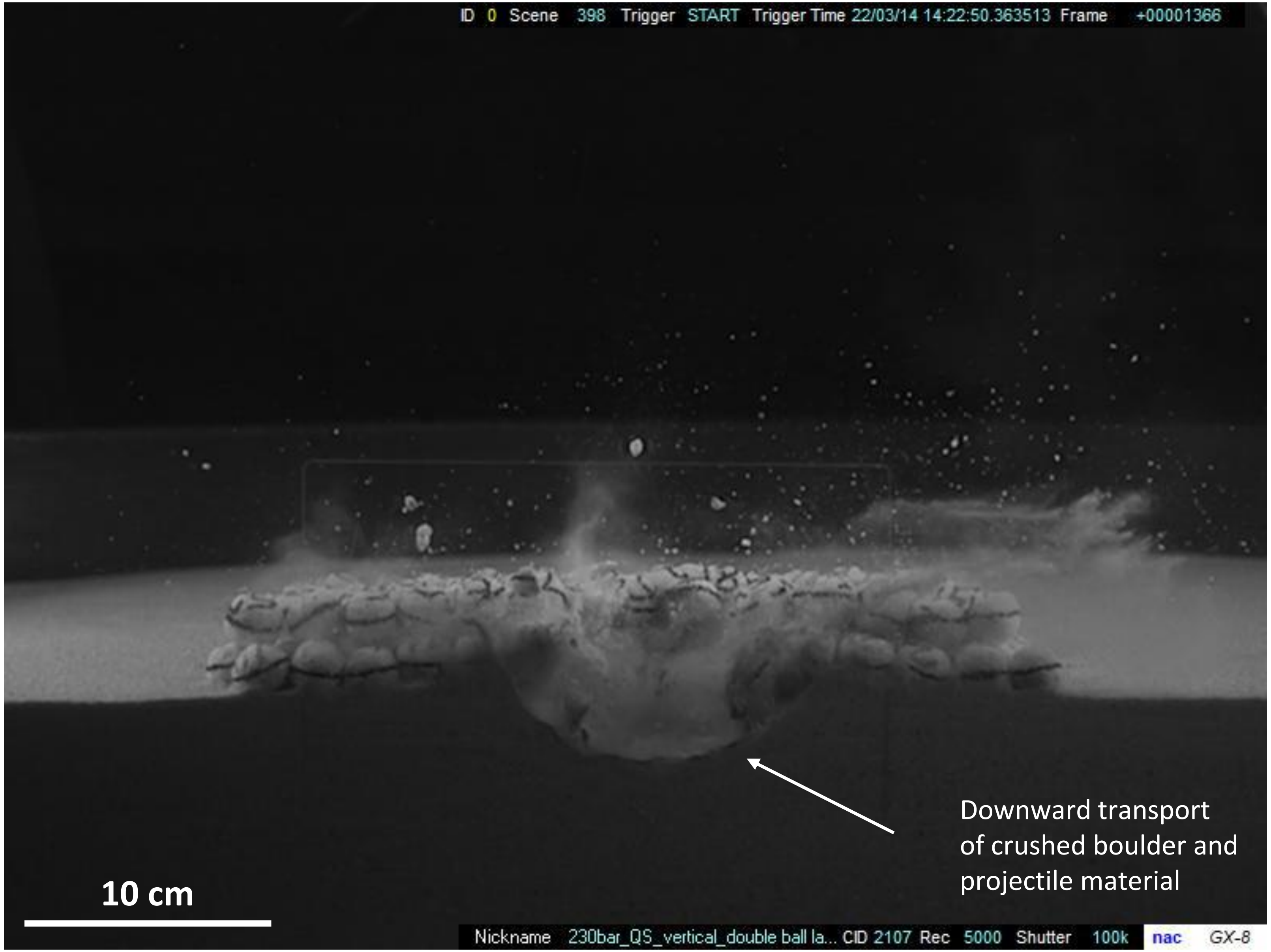
ID 0 Scene 398 Trigger START Trigger Time 22/03/14 14:22:50.363513 Frame +00001366
Downward transport
of crushed boulder and
projectile material
10 cm
Nickname 230bar_QS_vertical_double ball la... CID 2107 Rec 5000 Shutter 100k nac GX-8

“Exp 6”.

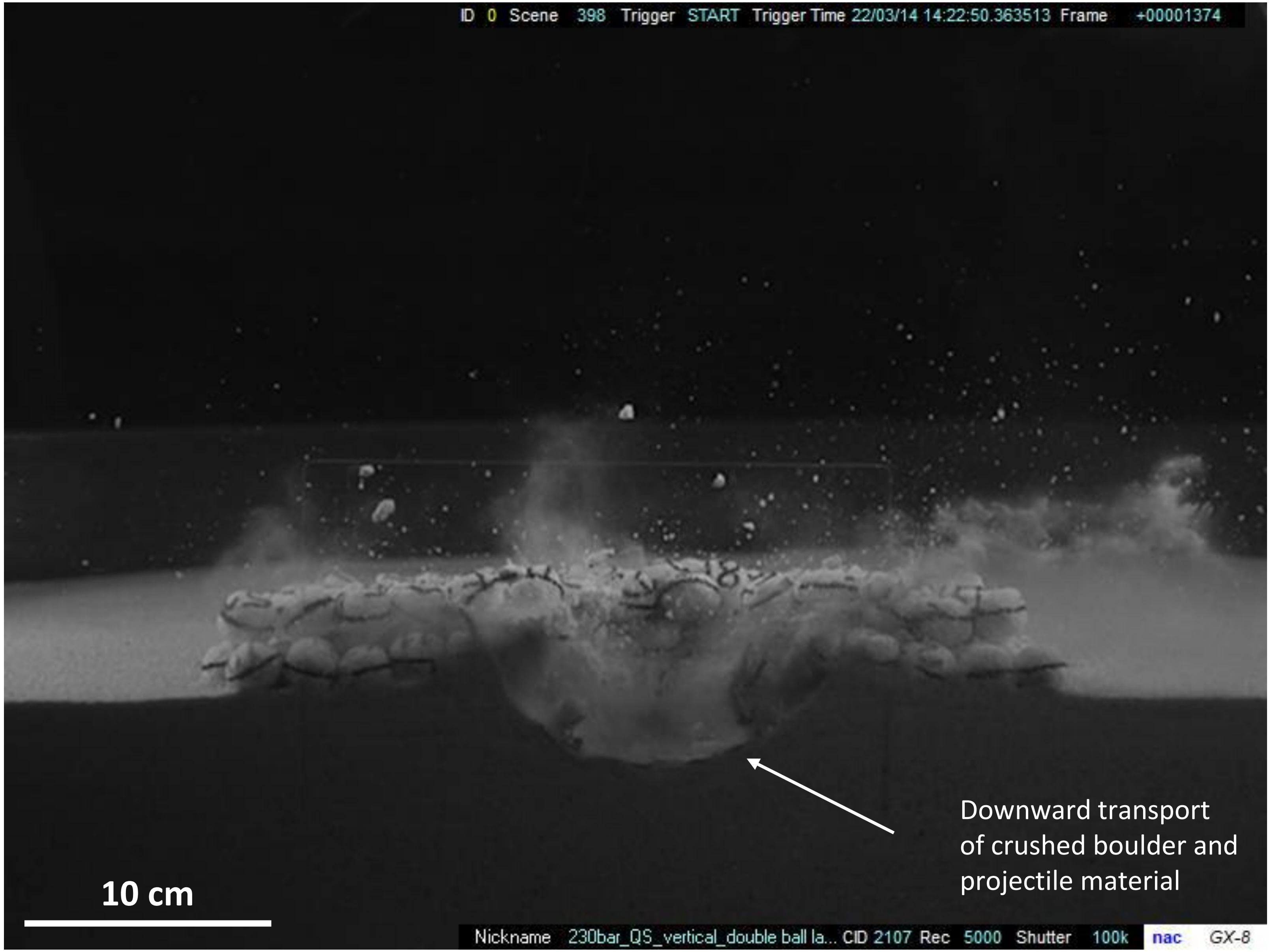
ID 0 Scene 398 Trigger START Trigger Time 22/03/14 14:22:50.363513 Frame +00001374
Downward transport of crushed boulder and projectile material
10 cm
Nickname 230bar_QS_vertical_double ball la... CID 2107 Rec 5000 Shutter 100k nac GX-8

“Exp 6”.

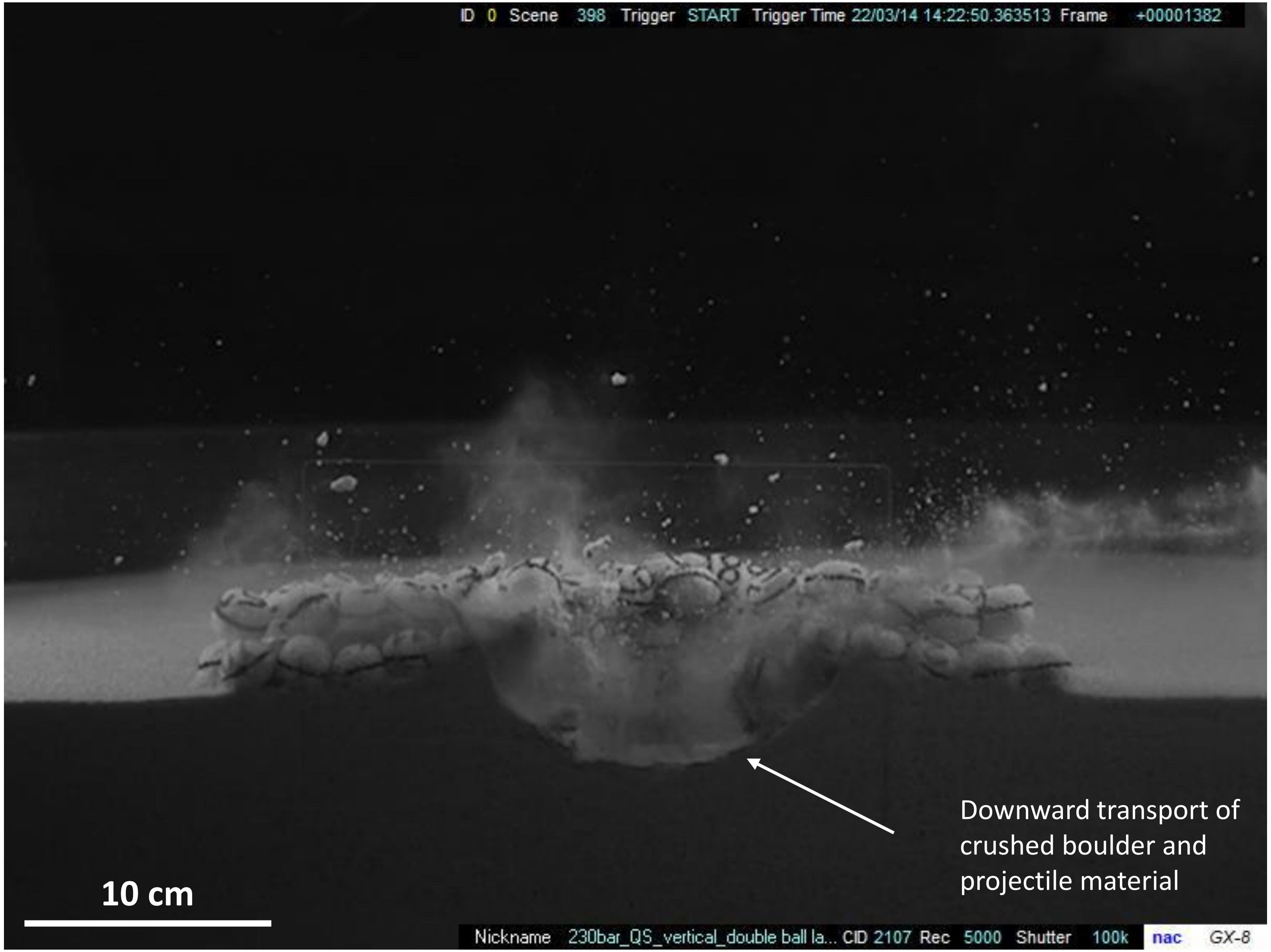
ID 0 Scene 398 Trigger START Trigger Time 22/03/14 14:22:50.363513 Frame +00001382
Downward transport of crushed boulder and projectile material
10 cm
Nickname 230bar_QS_vertical_double ball la... CID 2107 Rec 5000 Shutter 100k nac GX-8

“Exp 6”.

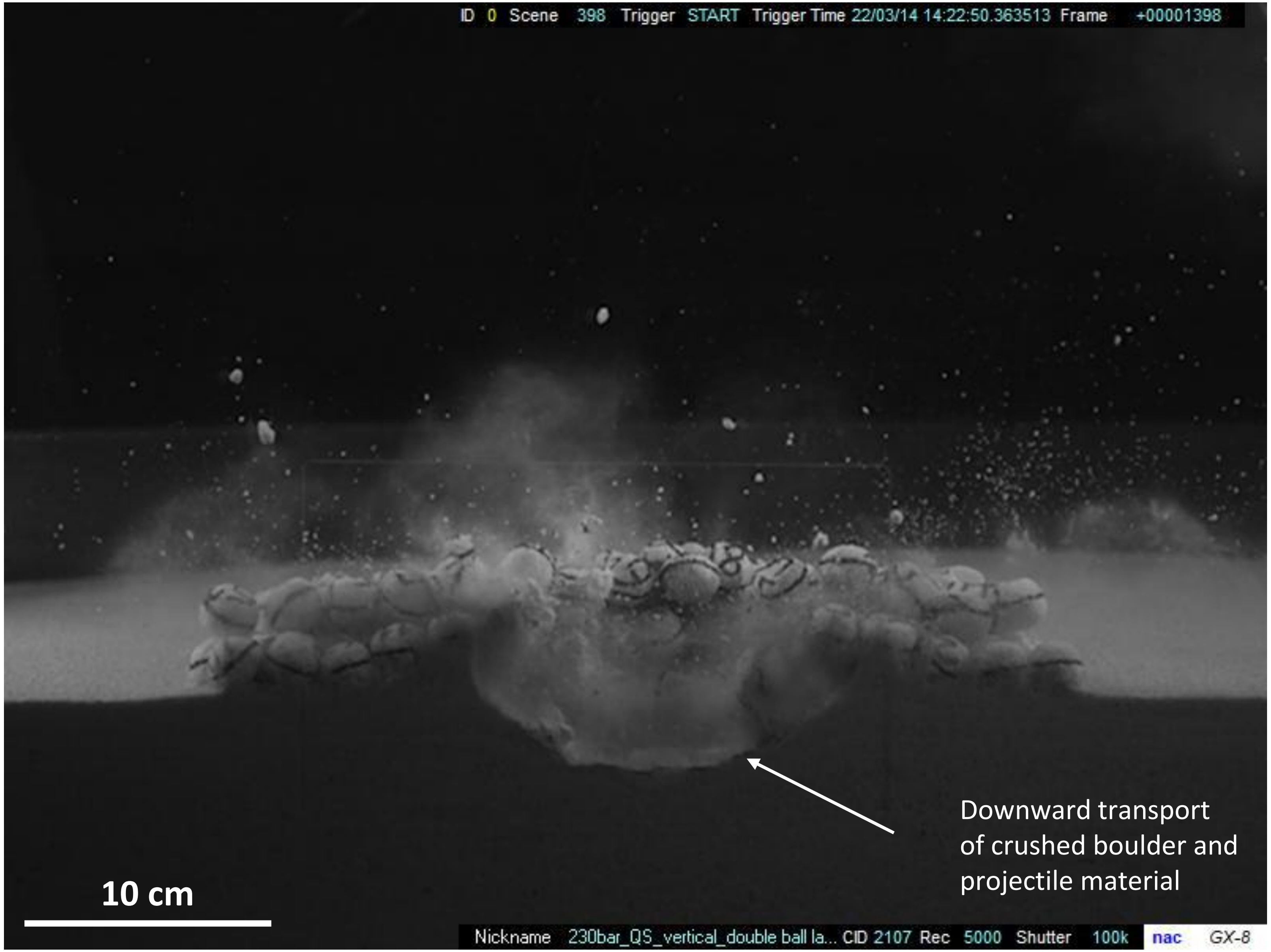
ID 0 Scene 398 Trigger START Trigger Time 22/03/14 14:22:50.363513 Frame +00001398
Downward transport
of crushed boulder and
projectile material
10 cm
Nickname 230bar_QS_vertical_double ball la... CID 2107 Rec 5000 Shutter 100k
nac GX-8

"Exp 6".

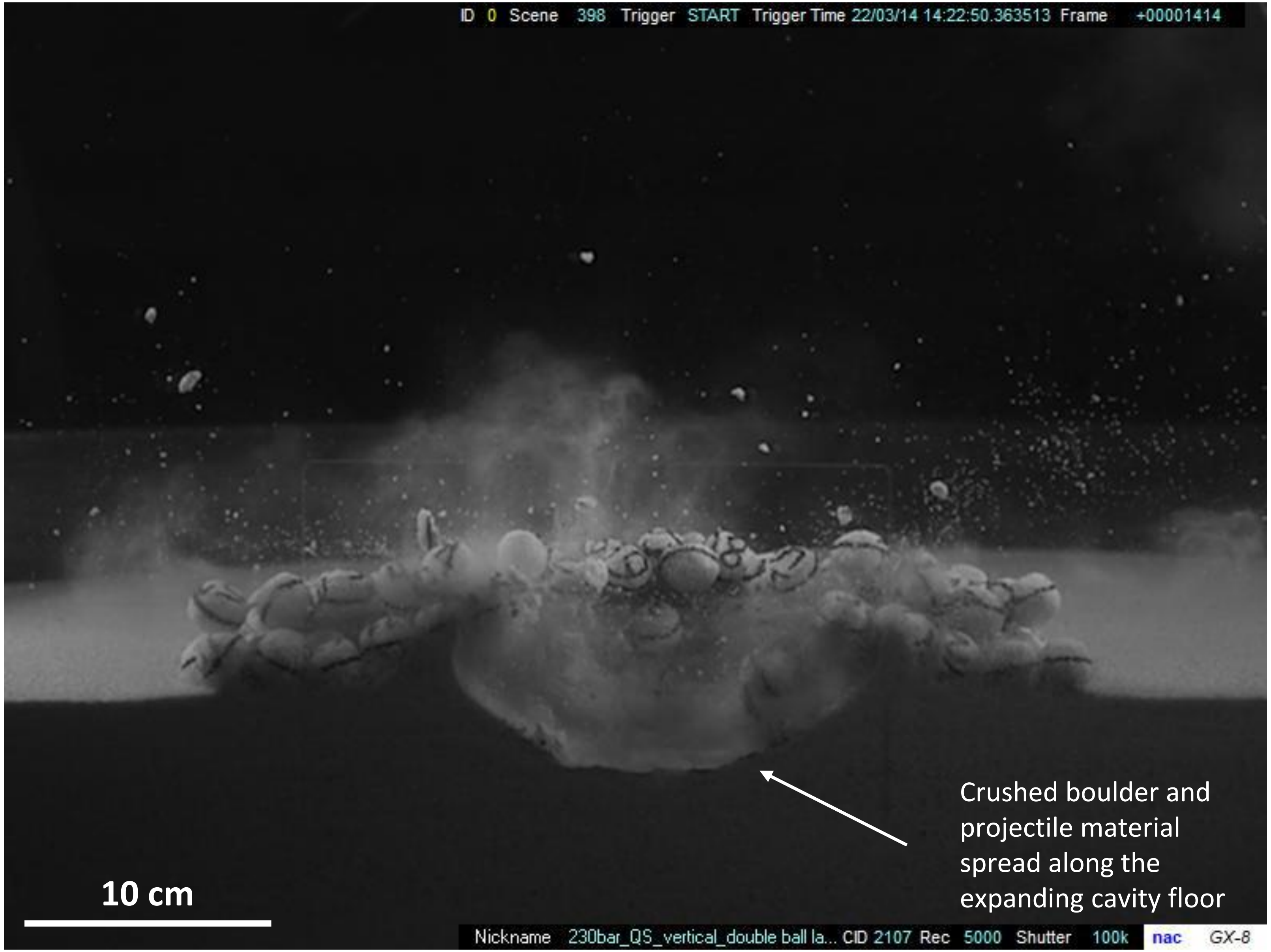
ID 0 Scene 398 Trigger START Trigger Time 22/03/14 14:22:50.363513 Frame +00001414
Crushed boulder and projectile material spread along the expanding cavity floor
10 cm
Nickname 230bar_QS_vertical_double ball la... CID 2107 Rec 5000 Shutter 100k nac GX-8

“Exp 6”.

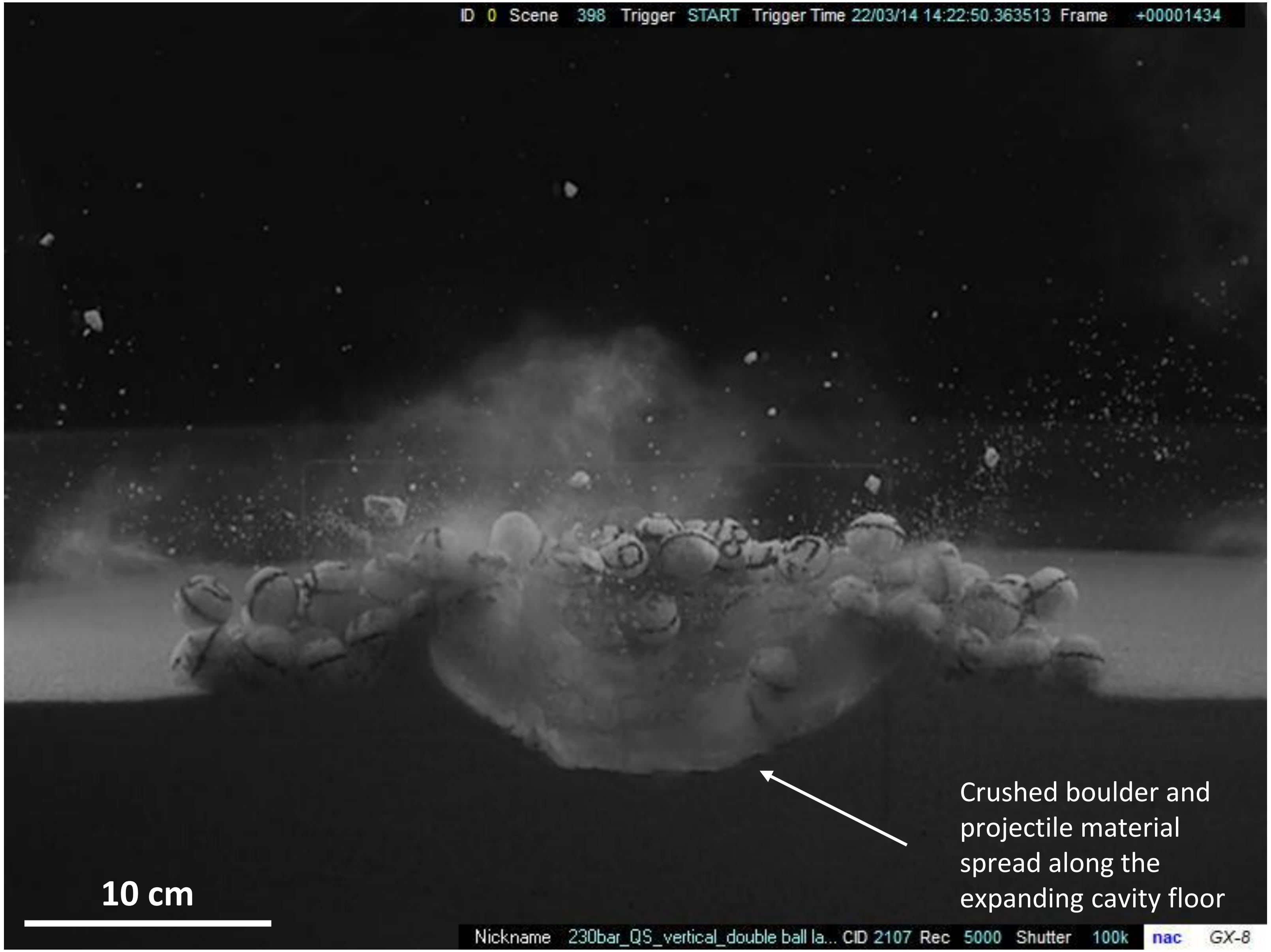
ID 0 Scene 398 Trigger START Trigger Time 22/03/14 14:22:50.363513 Frame +00001434
Crushed boulder and projectile material spread along the expanding cavity floor
10 cm
Nickname 230bar_QS_vertical_double ball la... CID 2107 Rec 5000 Shutter 100k nac GX-8

“Exp 6”.

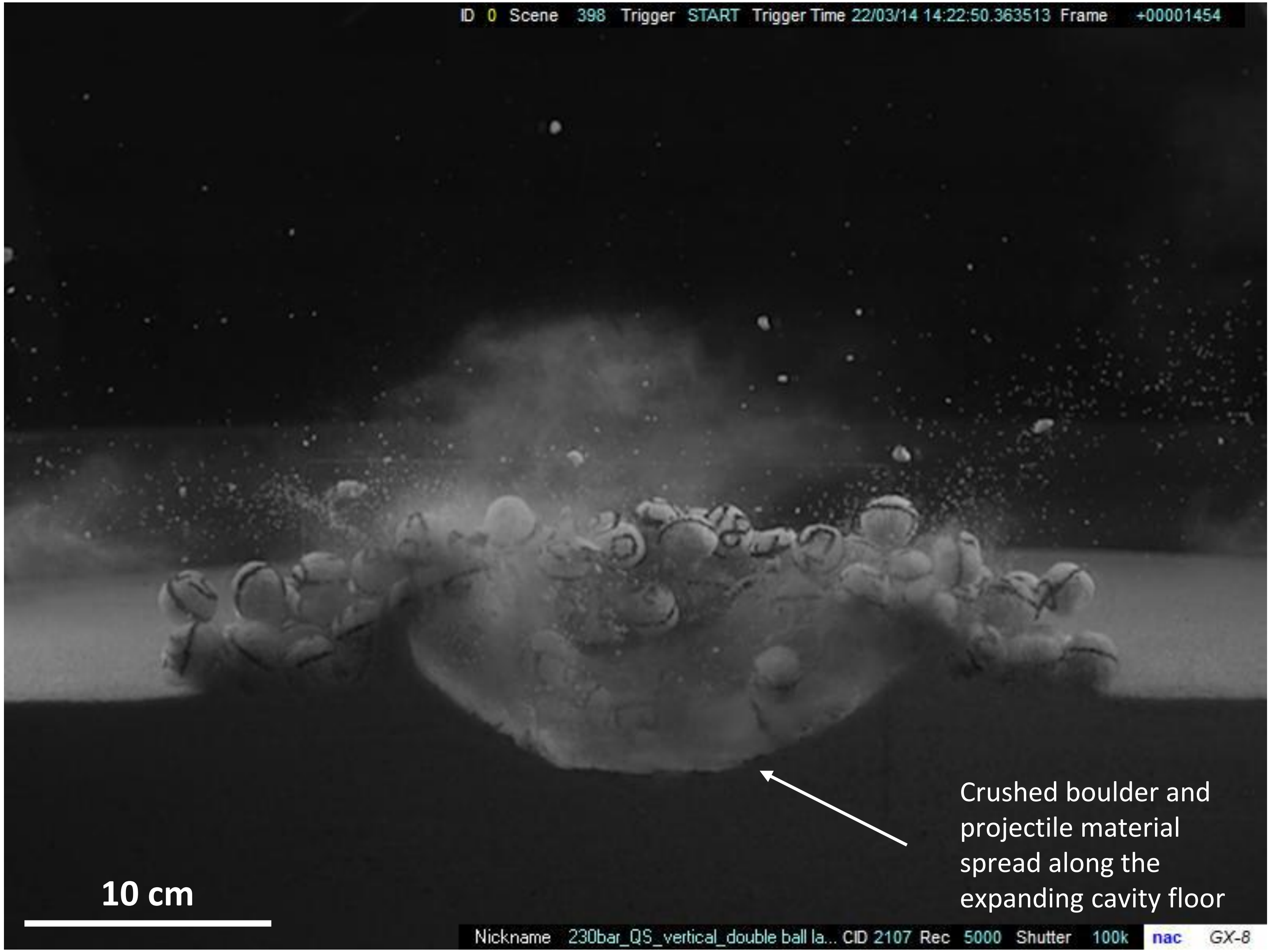
ID 0 Scene 398 Trigger START Trigger Time 22/03/14 14:22:50.363513 Frame +00001454
Crushed boulder and projectile material spread along the expanding cavity floor
10 cm
Nickname 230bar_QS_vertical_double ball la... CID 2107 Rec 5000 Shutter 100k nac GX-8

“Exp 6”.

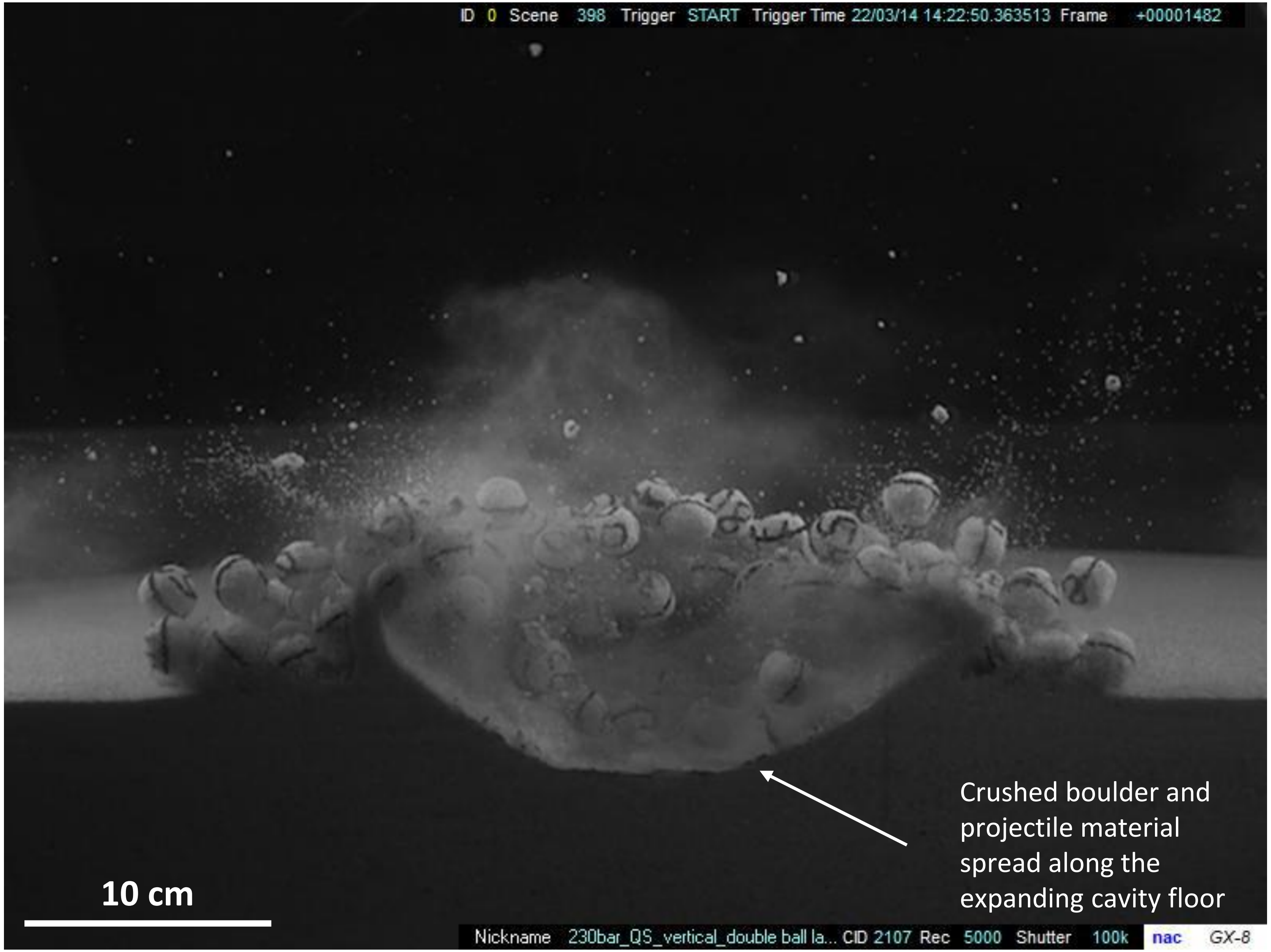
ID 0 Scene 398 Trigger START Trigger Time 22/03/14 14:22:50.363513 Frame +00001482
Crushed boulder and projectile material spread along the expanding cavity floor
10 cm
Nickname 230bar_QS_vertical_double ball la... CID 2107 Rec 5000 Shutter 100k nac GX-8

“Exp 6”.

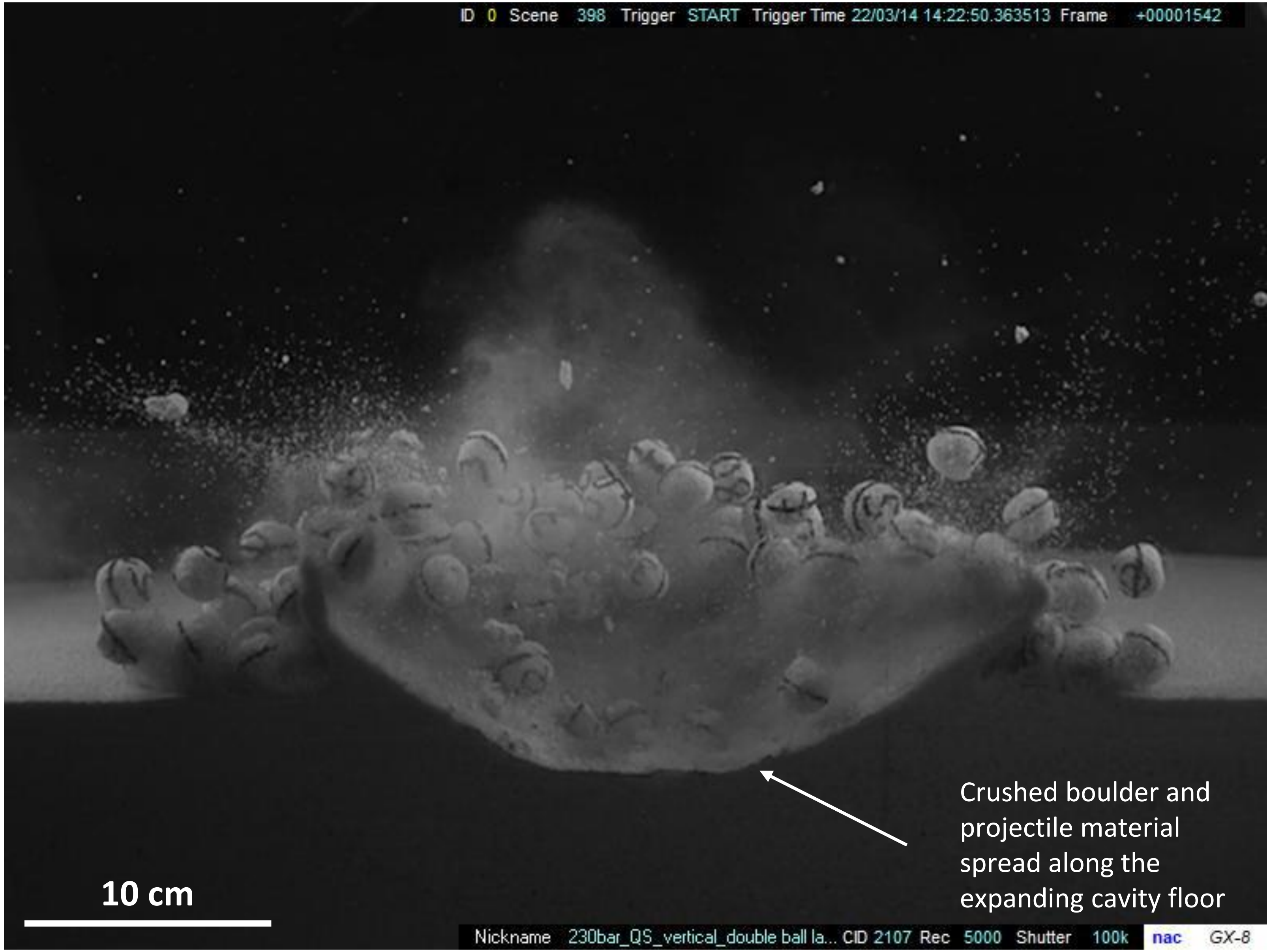
ID 0 Scene 398 Trigger START Trigger Time 22/03/14 14:22:50.363513 Frame +00001542
Crushed boulder and projectile material spread along the expanding cavity floor
10 cm
Nickname 230bar_QS_vertical_double ball la... CID 2107 Rec 5000 Shutter 100k
nac
GX-8

“Exp 6”.

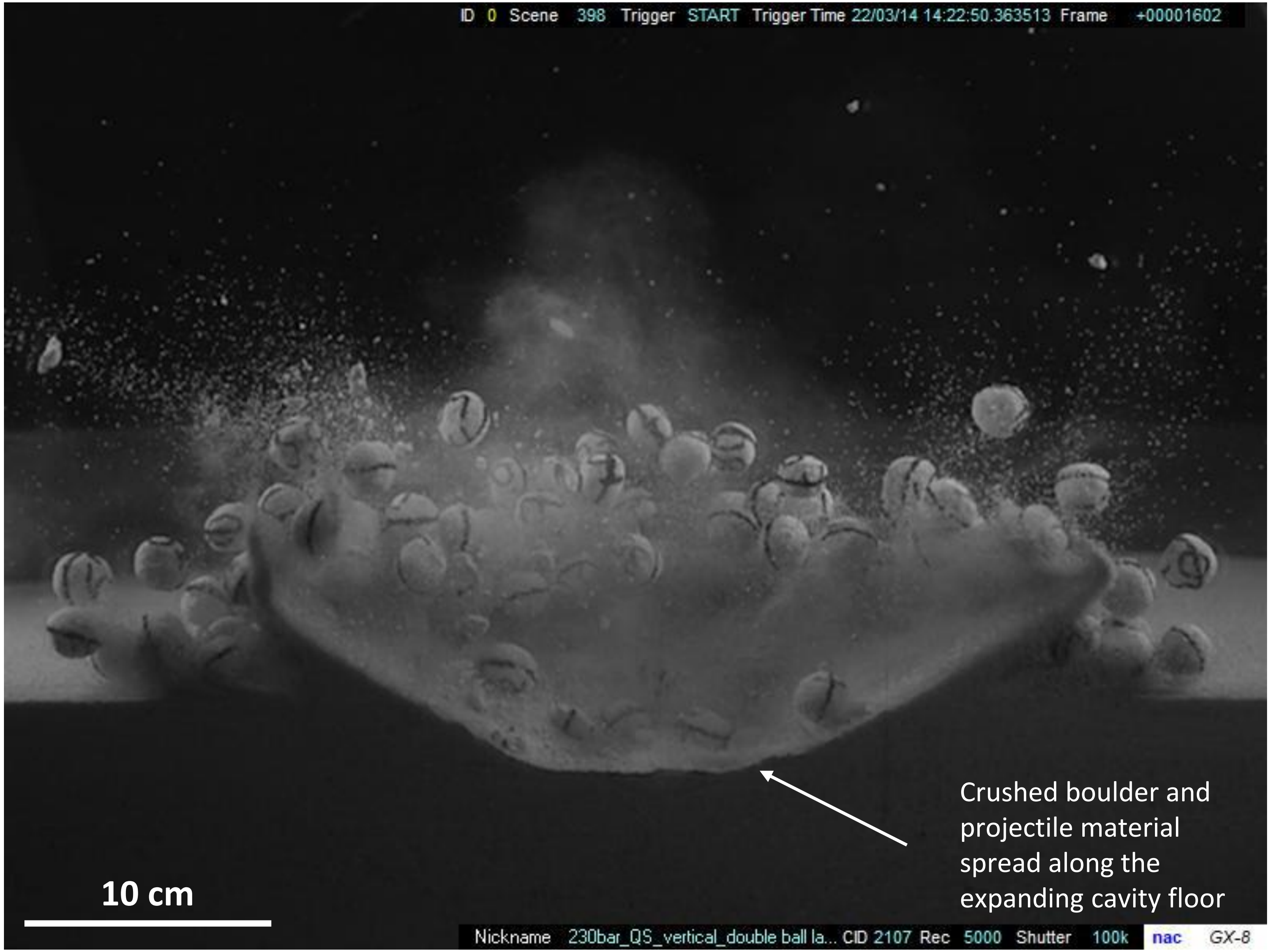
ID 0 Scene 398 Trigger START Trigger Time 22/03/14 14:22:50.363513 Frame +00001602
Crushed boulder and projectile material spread along the expanding cavity floor
10 cm
Nickname 230bar_QS_vertical_double ball la... CID 2107 Rec 5000 Shutter 100k nac GX-8

"Exp 6".

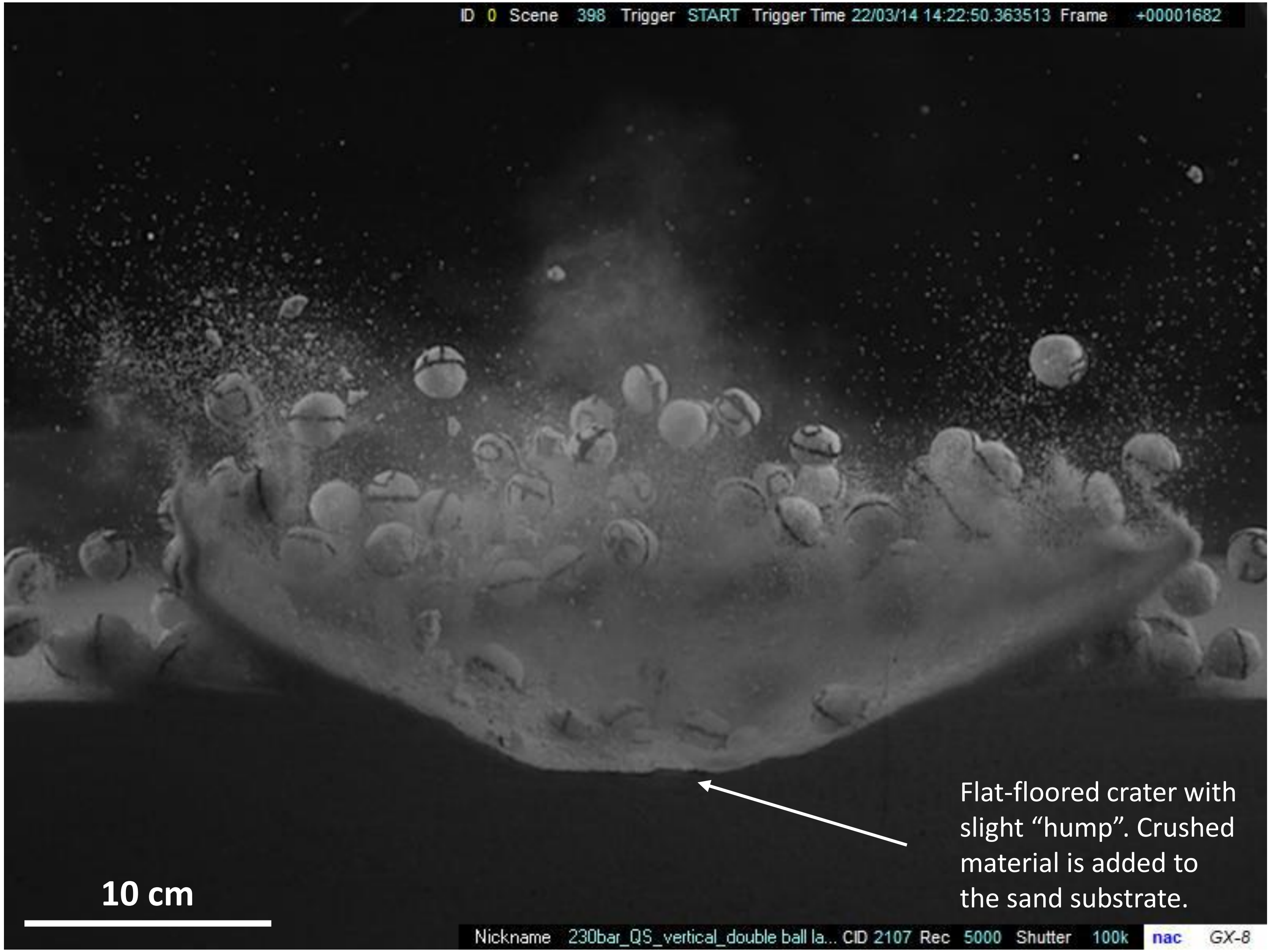
ID 0 Scene 398 Trigger START Trigger Time 22/03/14 14:22:50.363513 Frame +00001682
10 cm
Flat-floored crater with slight "hump". Crushed material is added to the sand substrate.
Nickname 230bar_QS_vertical_double ball la... CID 2107 Rec 5000 Shutter 100k nac GX-8

“Exp 6”.

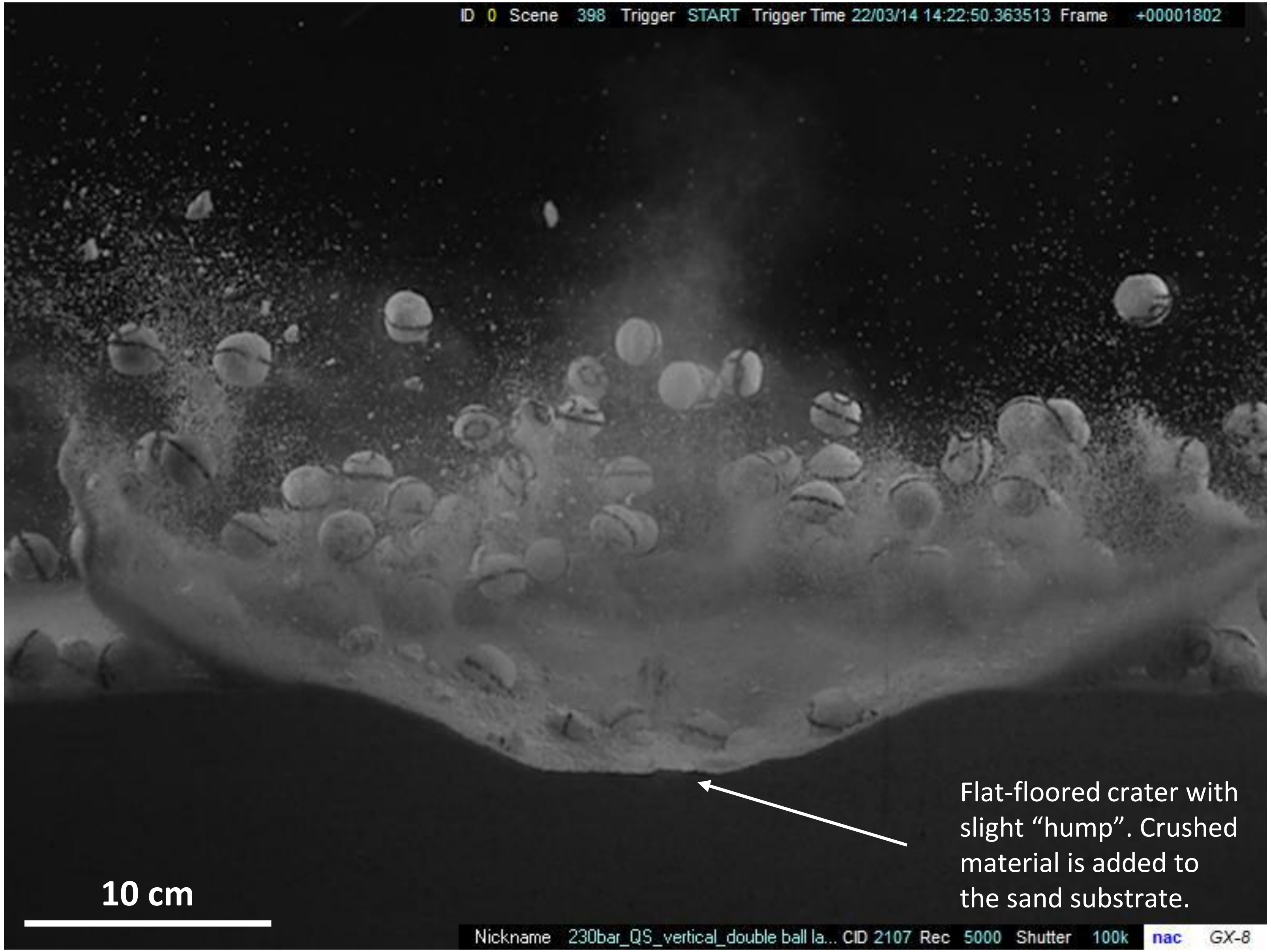
ID 0 Scene 398 Trigger START Trigger Time 22/03/14 14:22:50.363513 Frame +00001802
Flat-floored crater with slight “hump”. Crushed material is added to the sand substrate.
10 cm
Nickname 230bar_QS_vertical_double ball la... CID 2107 Rec 5000 Shutter 100k nac GX-8

"Exp 6".

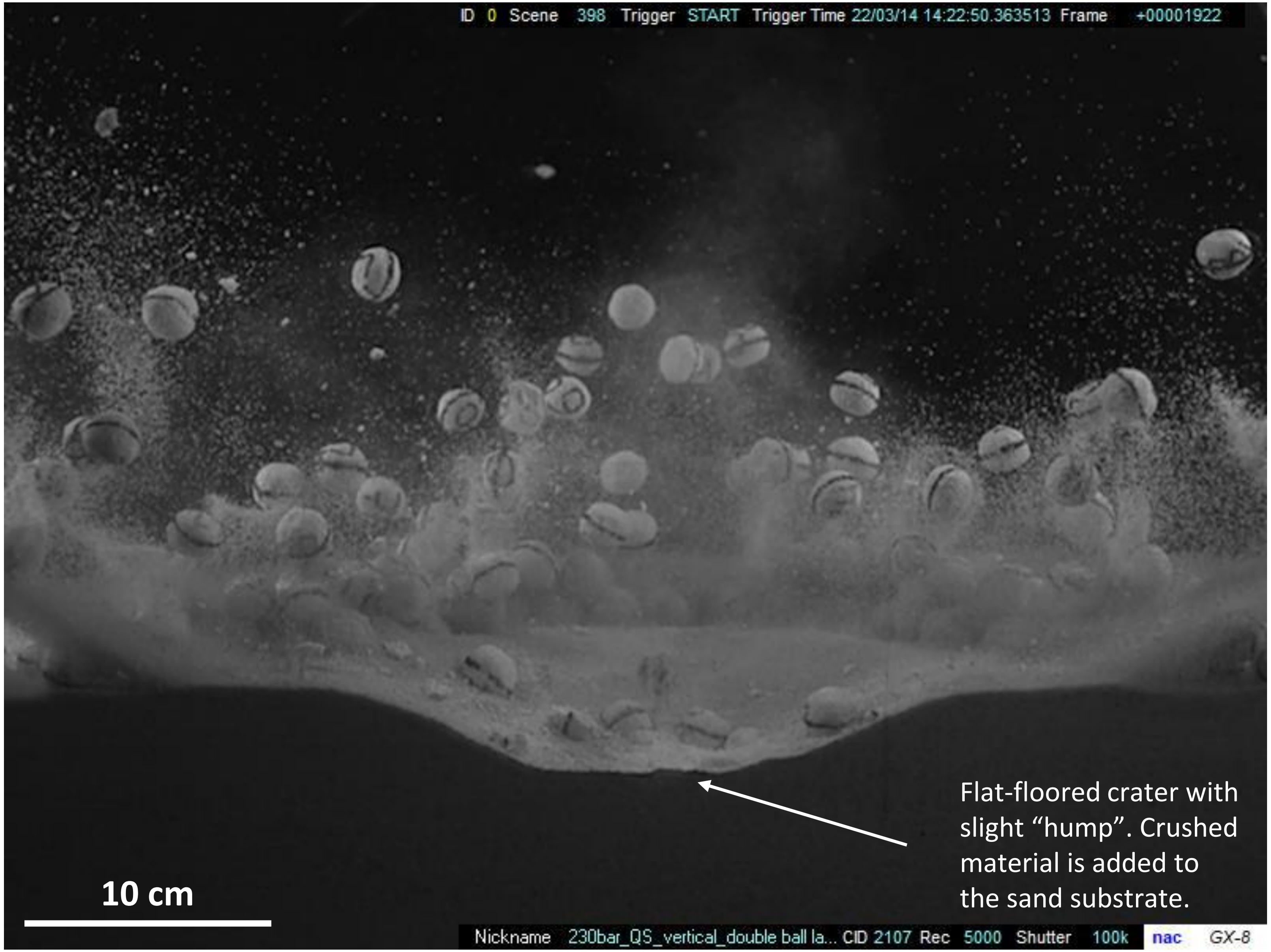
ID 0 Scene 398 Trigger START Trigger Time 22/03/14 14:22:50.363513 Frame +00001922
Flat-floored crater with slight "hump". Crushed material is added to the sand substrate.
10 cm
Nickname 230bar_QS_vertical_double ball la... CID 2107 Rec 5000 Shutter 100k nac GX-8

“Exp 6”.

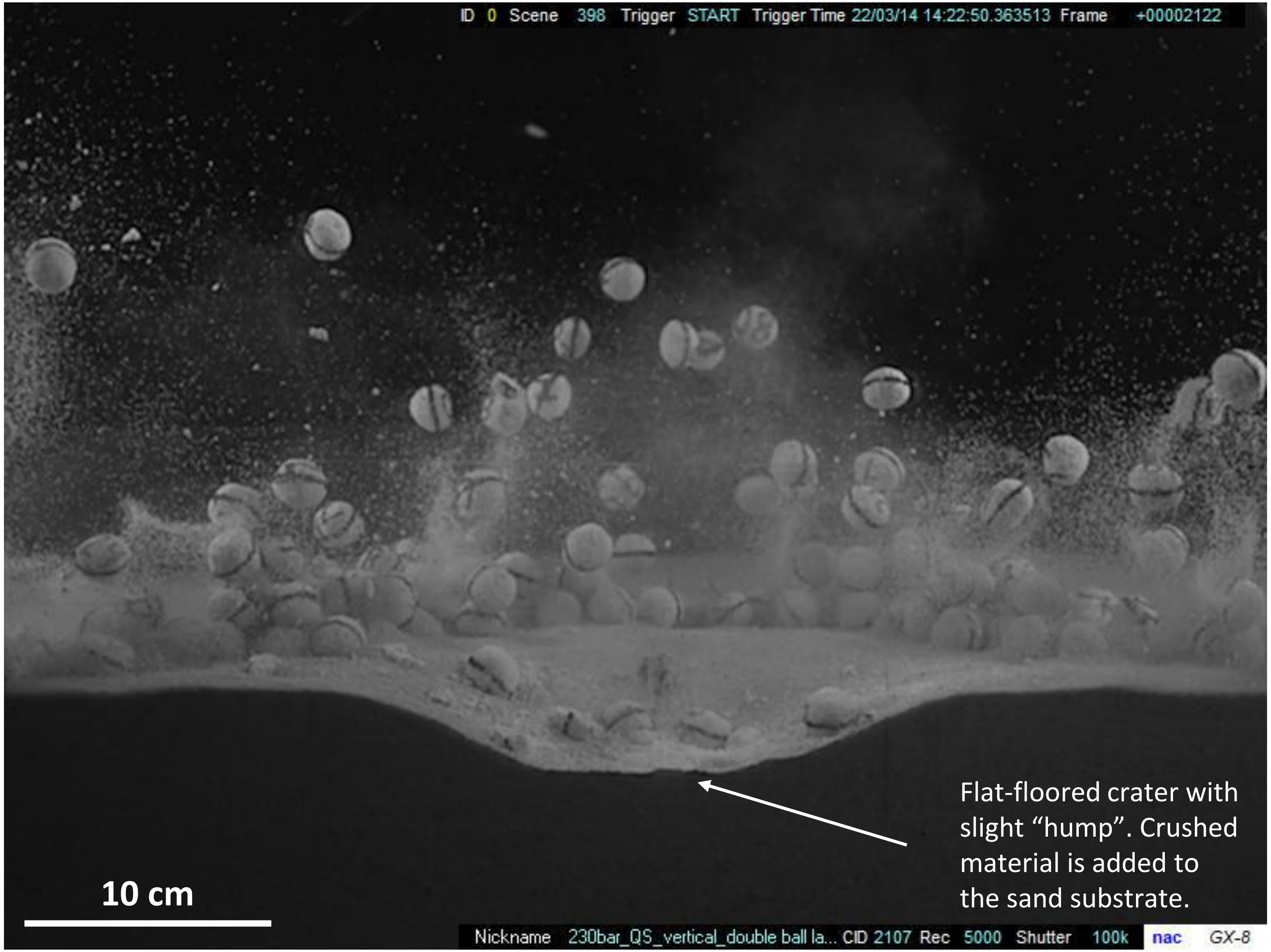
ID 0 Scene 398 Trigger START Trigger Time 22/03/14 14:22:50.363513 Frame +00002122
Flat-floored crater with slight “hump”. Crushed material is added to the sand substrate.
10 cm
Nickname 230bar_QS_vertical_double ball la... CID 2107 Rec 5000 Shutter 100k nac GX-8

“Exp 6”.

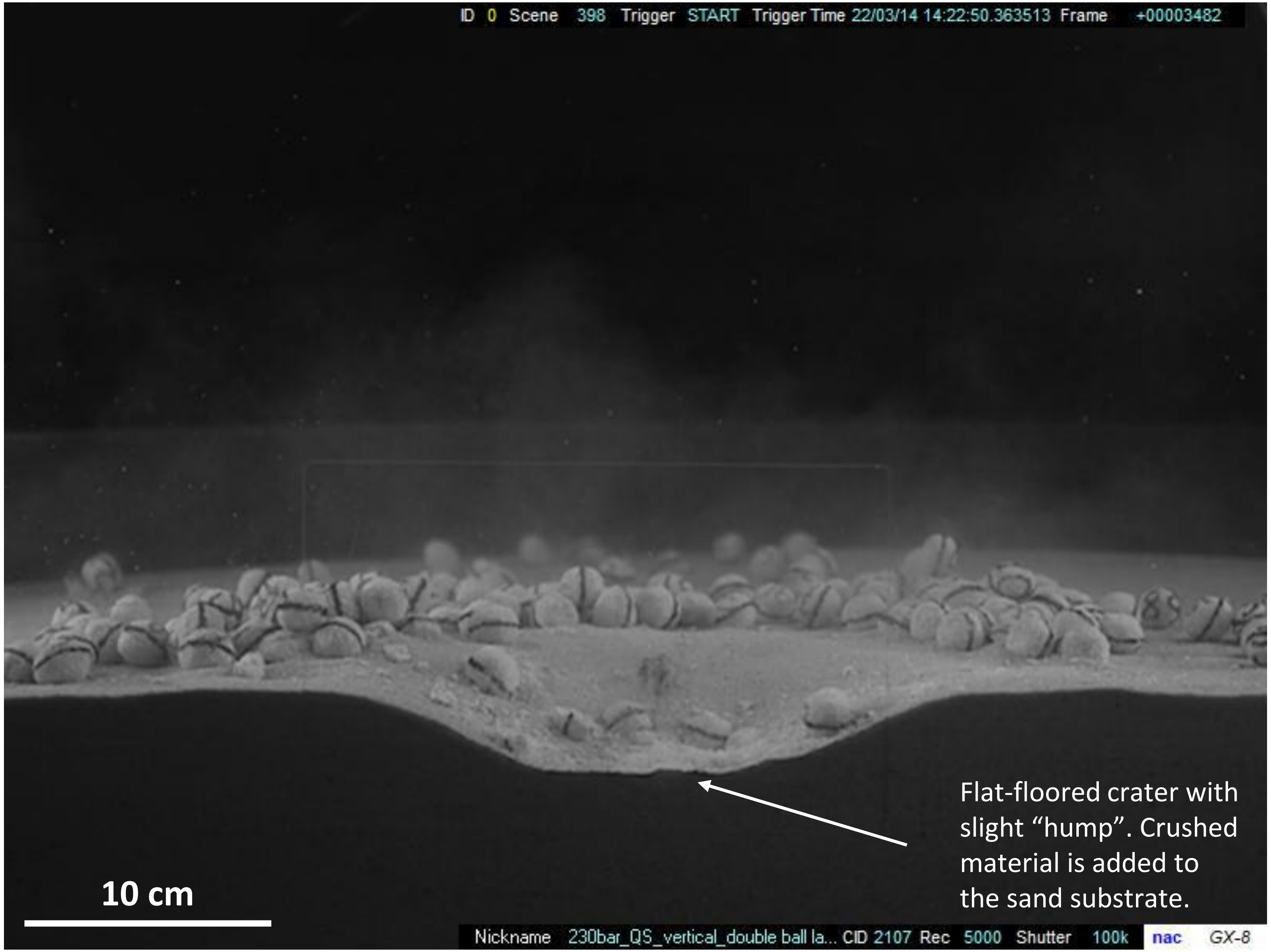
ID 0 Scene 398 Trigger START Trigger Time 22/03/14 14:22:50.363513 Frame +00003482
Flat-floored crater with slight “hump”. Crushed material is added to the sand substrate.
10 cm
Nickname 230bar_QS_vertical_double ball la... CID 2107 Rec 5000 Shutter 100k nac GX-8

Parameter study:
Test of influence by compaction of homogeneous sand target (i.e. "slow" vs "fast" pour)

Experiment set-up

Projectile:
- Delrin (disrupts upon impact)
- 20 mm diameter
- 5.7g
- 1.36g/cm^3
- Velocity:~408 m/s [measured in camera frames]

Target:

"Exp3": Beach sand (slow pour)
- Sand density:~1.8 g/cm^3
- Sand porosity:~32%

"Exp4": Beach sand (fast pour)
- Sand density:~1.6 g/cm^3
- Sand porosity:~40%

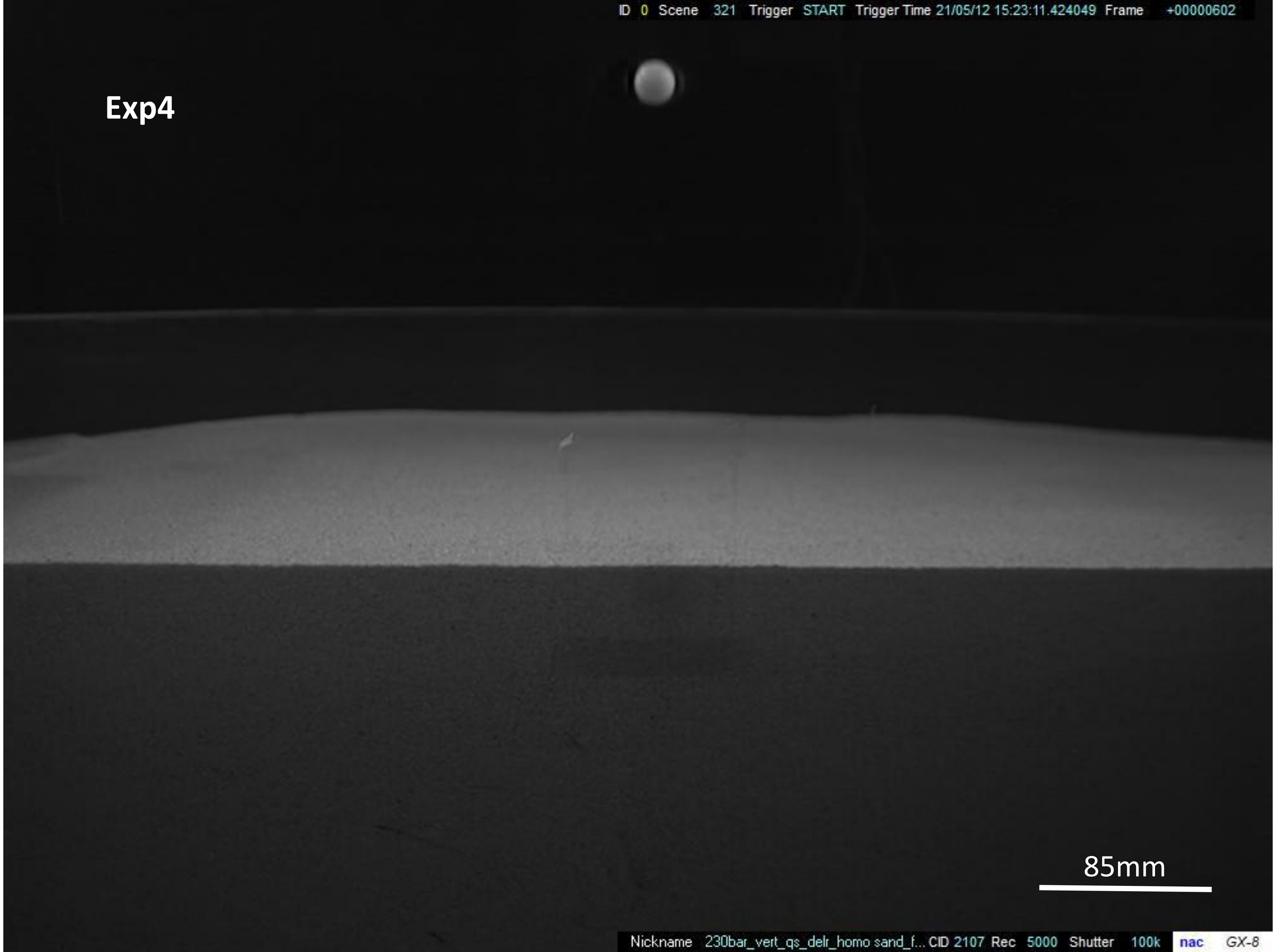
ID 0 Scene 321 Trigger START Trigger Time 21/05/12 15:23:11.424049 Frame +00000602
Exp4
85mm
Nickname 230bar_vert_qs_delr_homo sand_f... CID 2107 Rec 5000 Shutter 100k
nac GX-8

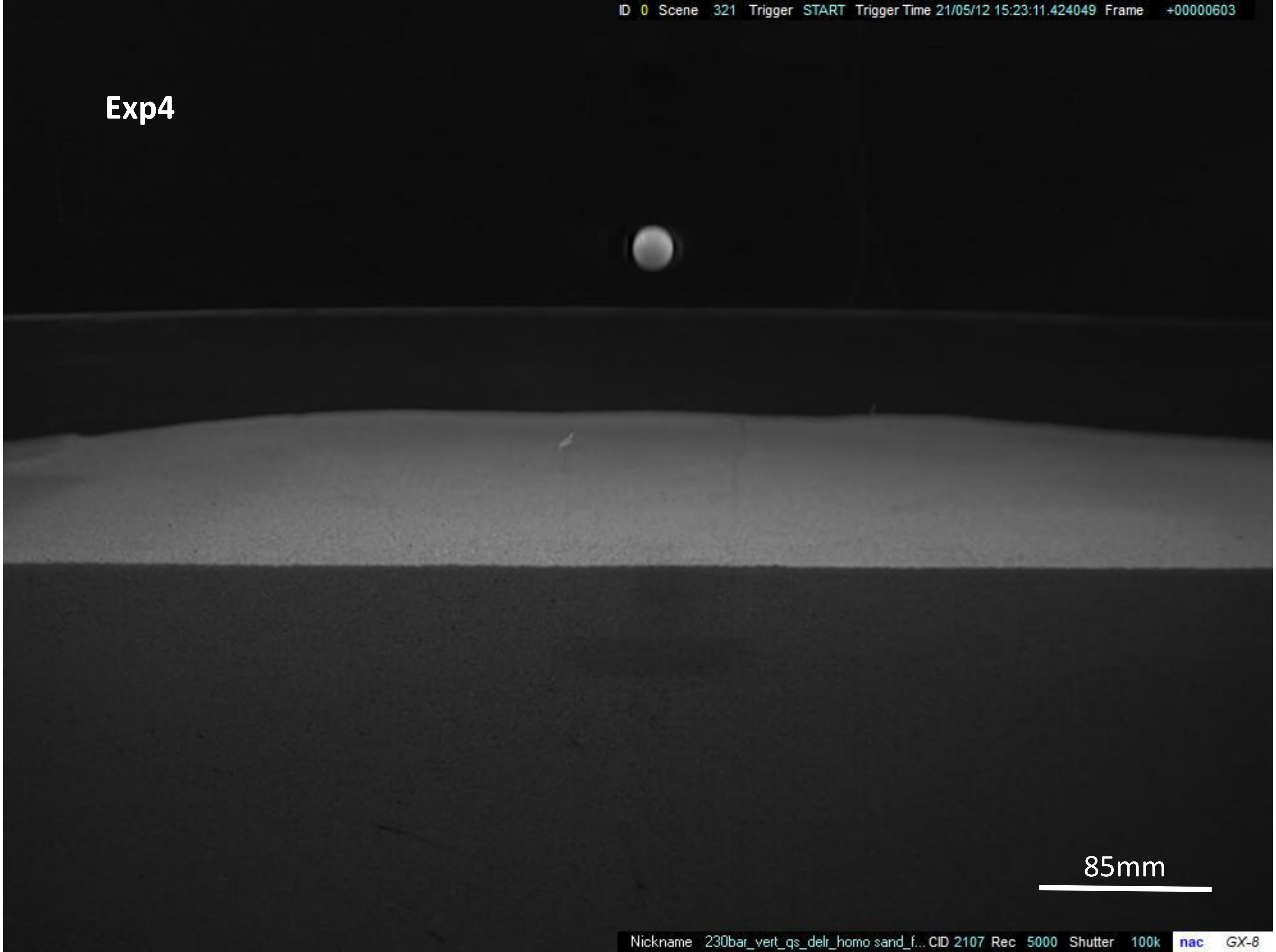
ID 0 Scene 321 Trigger START Trigger Time 21/05/12 15:23:11.424049 Frame +00000603
Exp4
85mm
Nickname 230bar_vert_qs_delr_homo sand_f... CID 2107 Rec 5000 Shutter 100k nac GX-8

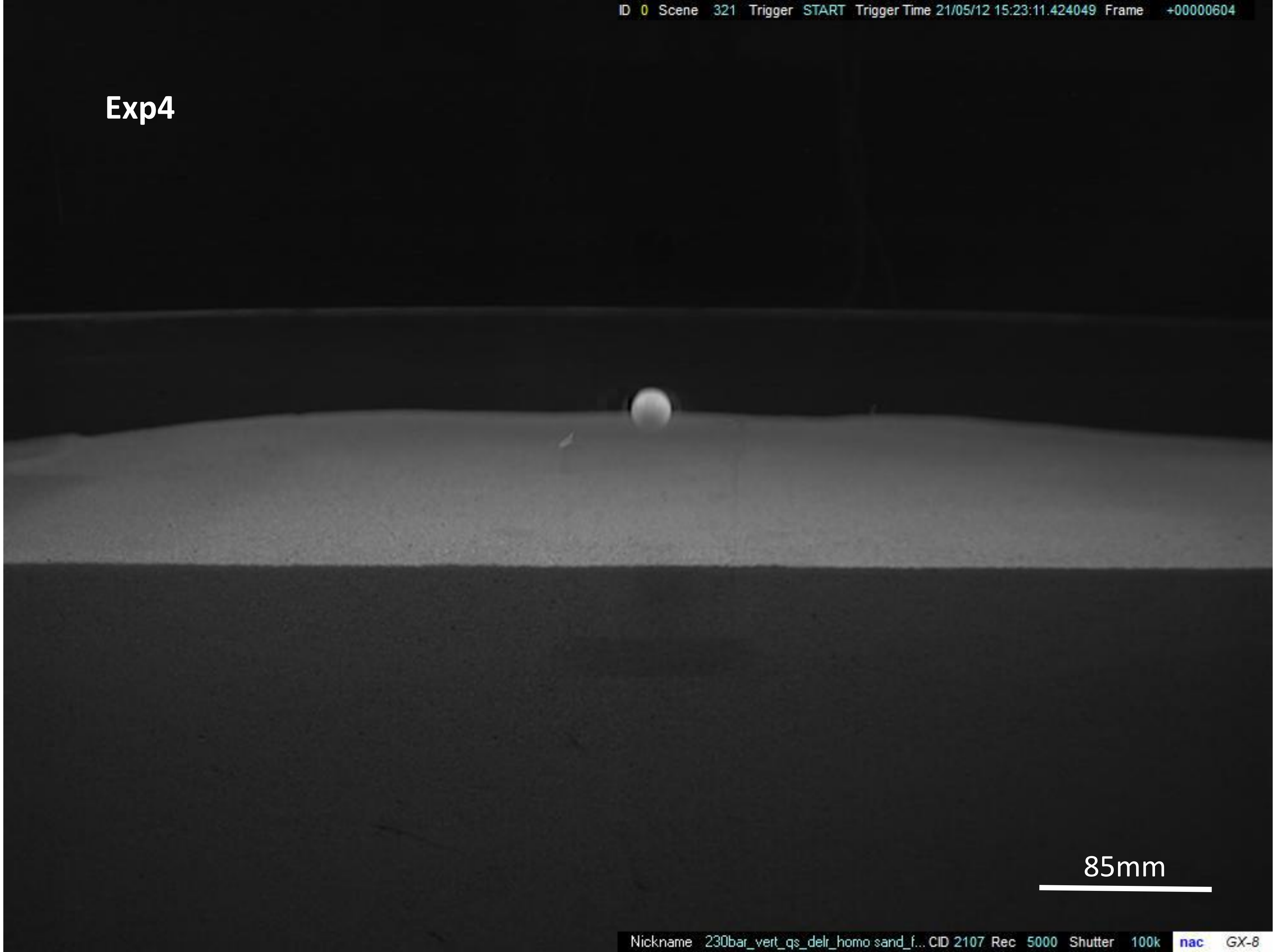
ID 0 Scene 321 Trigger START Trigger Time 21/05/12 15:23:11.424049 Frame +00000604
Exp4
85mm
Nickname 230bar_vert_qs_delr_homo sand_f... CID 2107 Rec 5000 Shutter 100k nac GX-8

T: 0sec

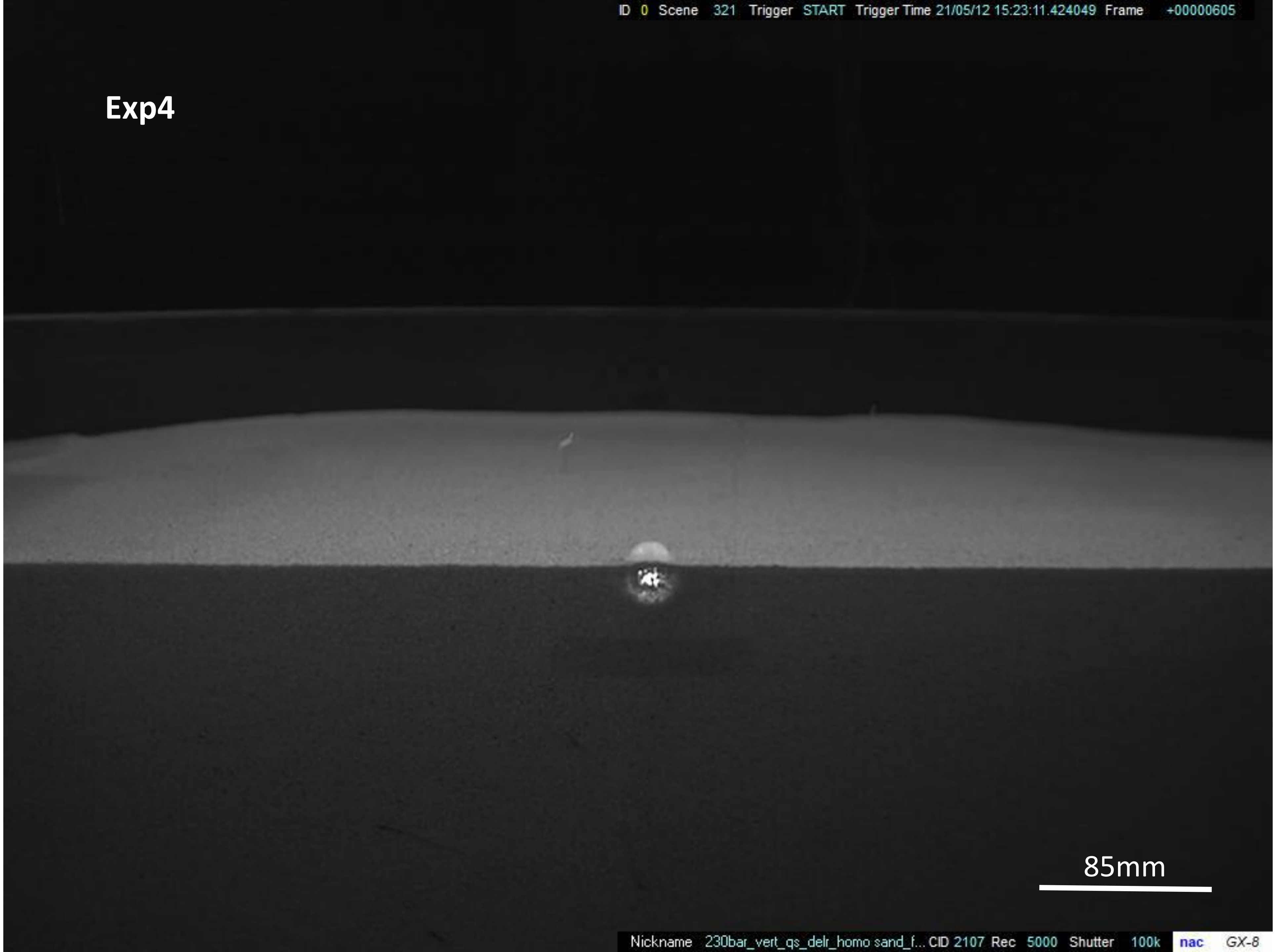

ID 0 Scene 321 Trigger START Trigger Time 21/05/12 15:23:11.424049 Frame +00000605
Exp4
85mm
Nickname 230bar_vert_qs_delr_homo sand_f... CID 2107 Rec 5000 Shutter 100k
nac
GX-8

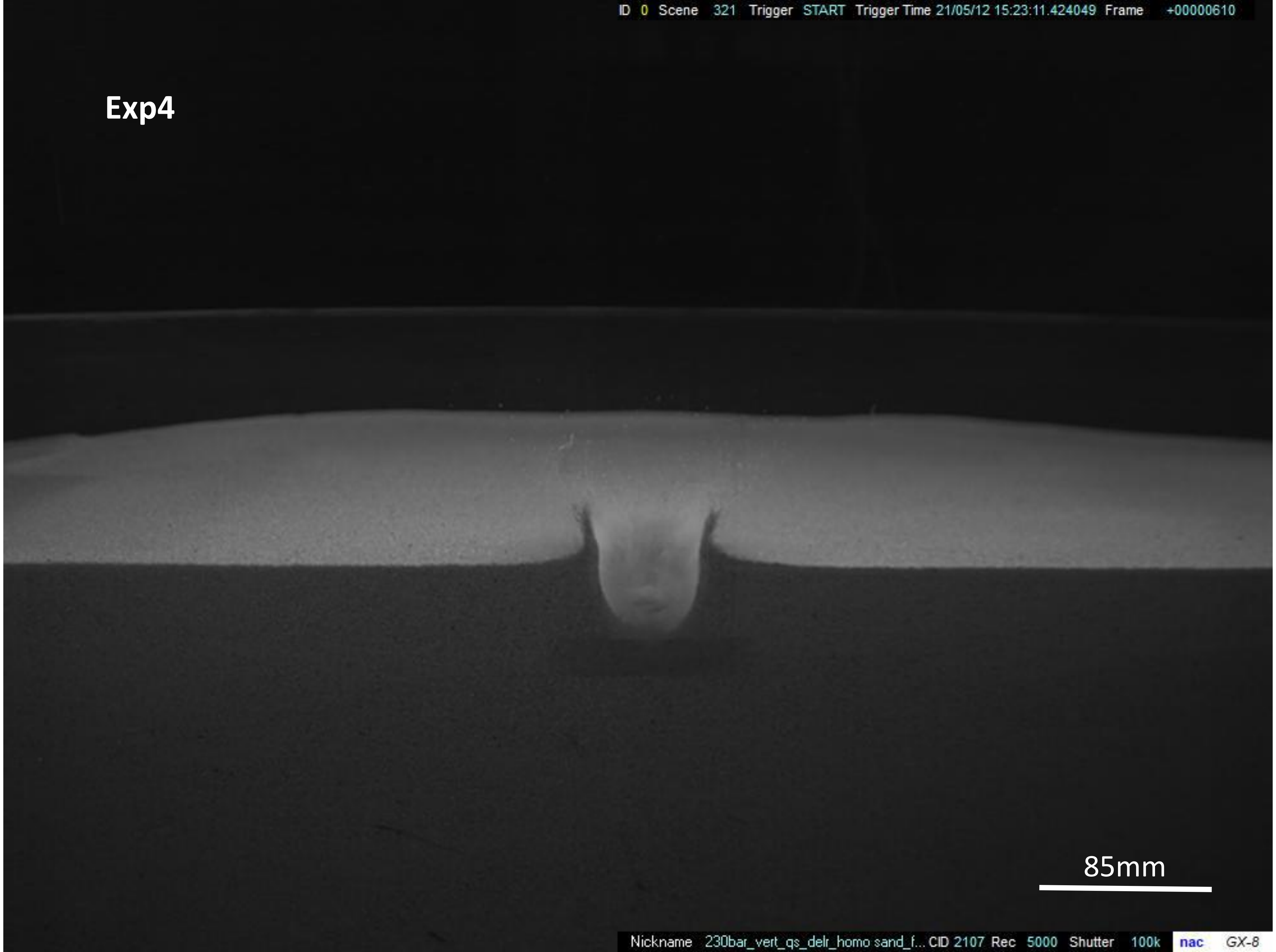
ID 0 Scene 321 Trigger START Trigger Time 21/05/12 15:23:11.424049 Frame +00000610
Exp4
85mm
Nickname 230bar_vert_qs_delr_homo sand_f... CID 2107 Rec 5000 Shutter 100k nac GX-8

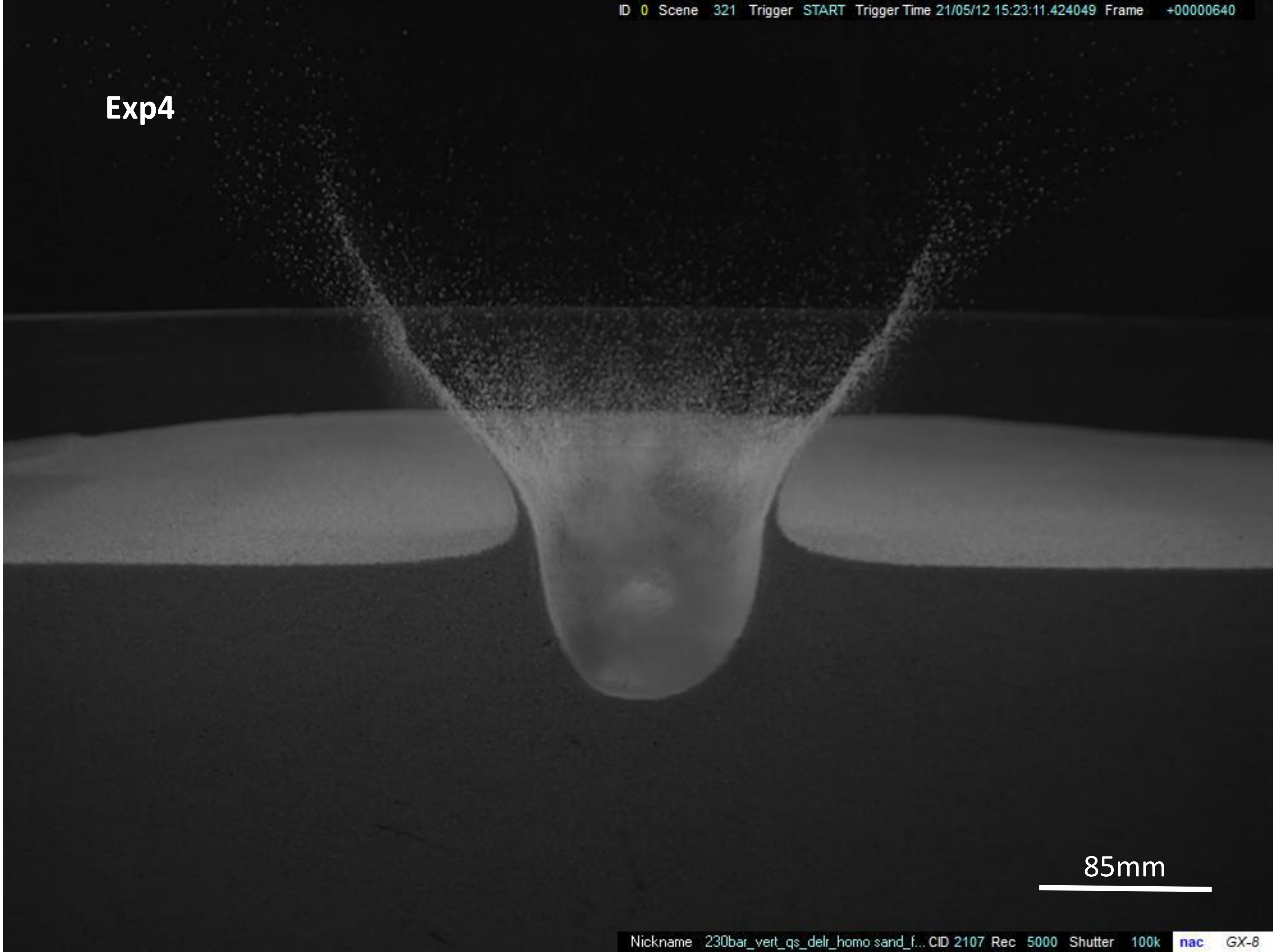
ID 0 Scene 321 Trigger START Trigger Time 21/05/12 15:23:11.424049 Frame +00000640
Exp4
85mm
Nickname 230bar_vert_qs_delr_homo sand_f... CID 2107 Rec 5000 Shutter 100k nac GX-8

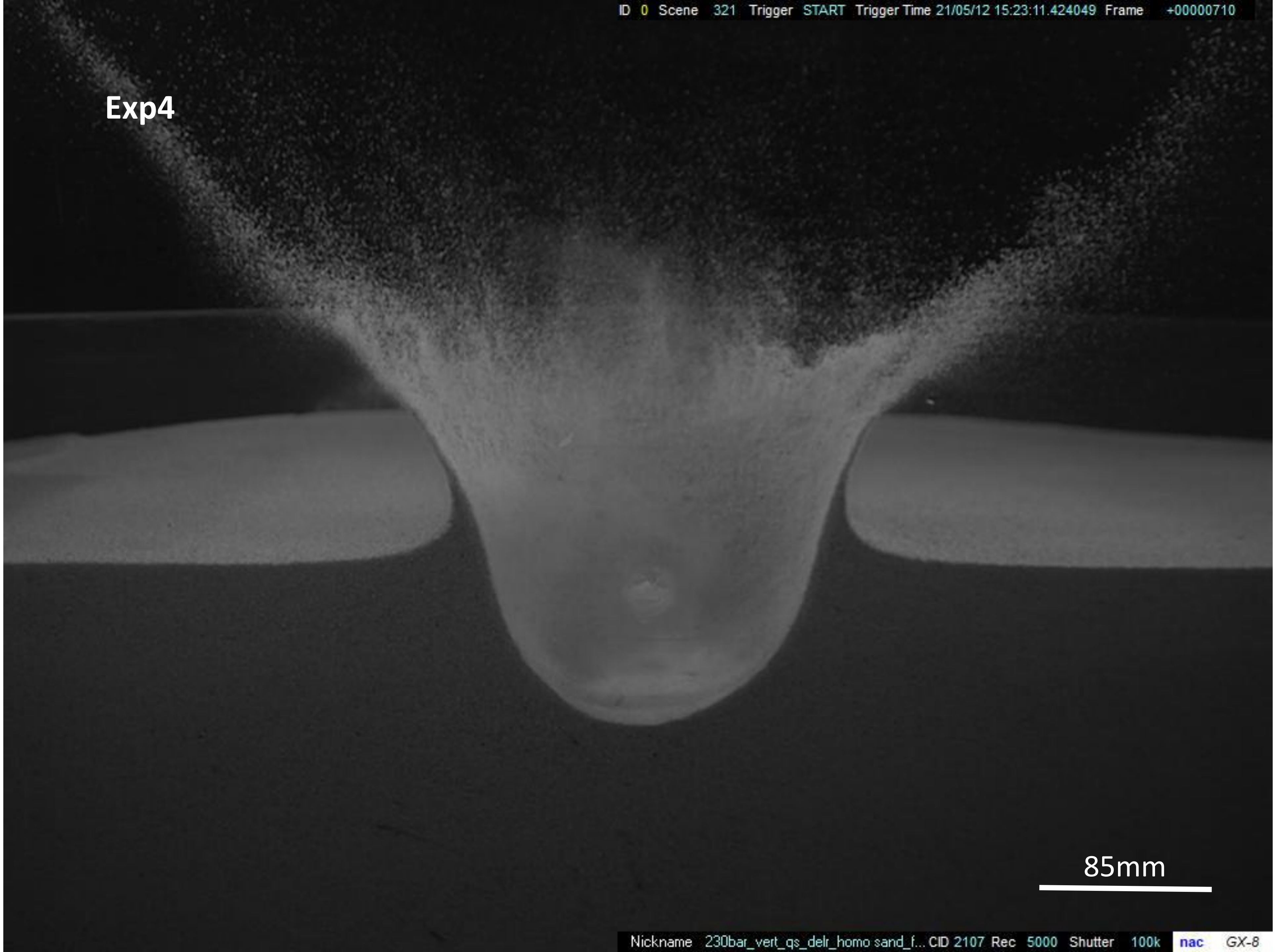
ID 0 Scene 321 Trigger START Trigger Time 21/05/12 15:23:11.424049 Frame +00000710
Exp4
85mm
Nickname 230bar_vert_qs_delr_homo sand_f... CID 2107 Rec 5000 Shutter 100k nac GX-8

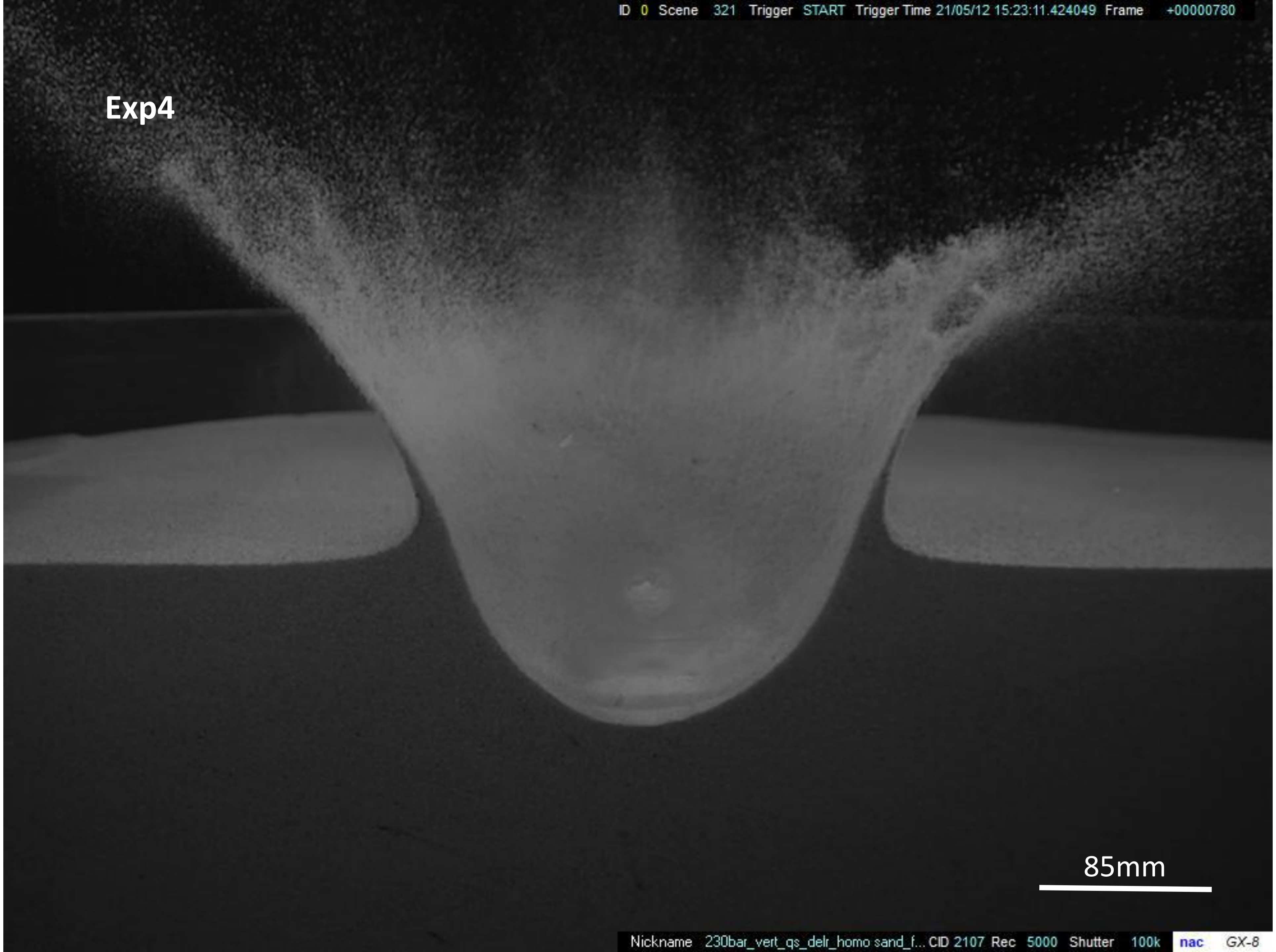
ID 0 Scene 321 Trigger START Trigger Time 21/05/12 15:23:11.424049 Frame +00000780
Exp4
85mm
Nickname 230bar_vert_qs_delr_homo sand_f... CID 2107 Rec 5000 Shutter 100k nac GX-8

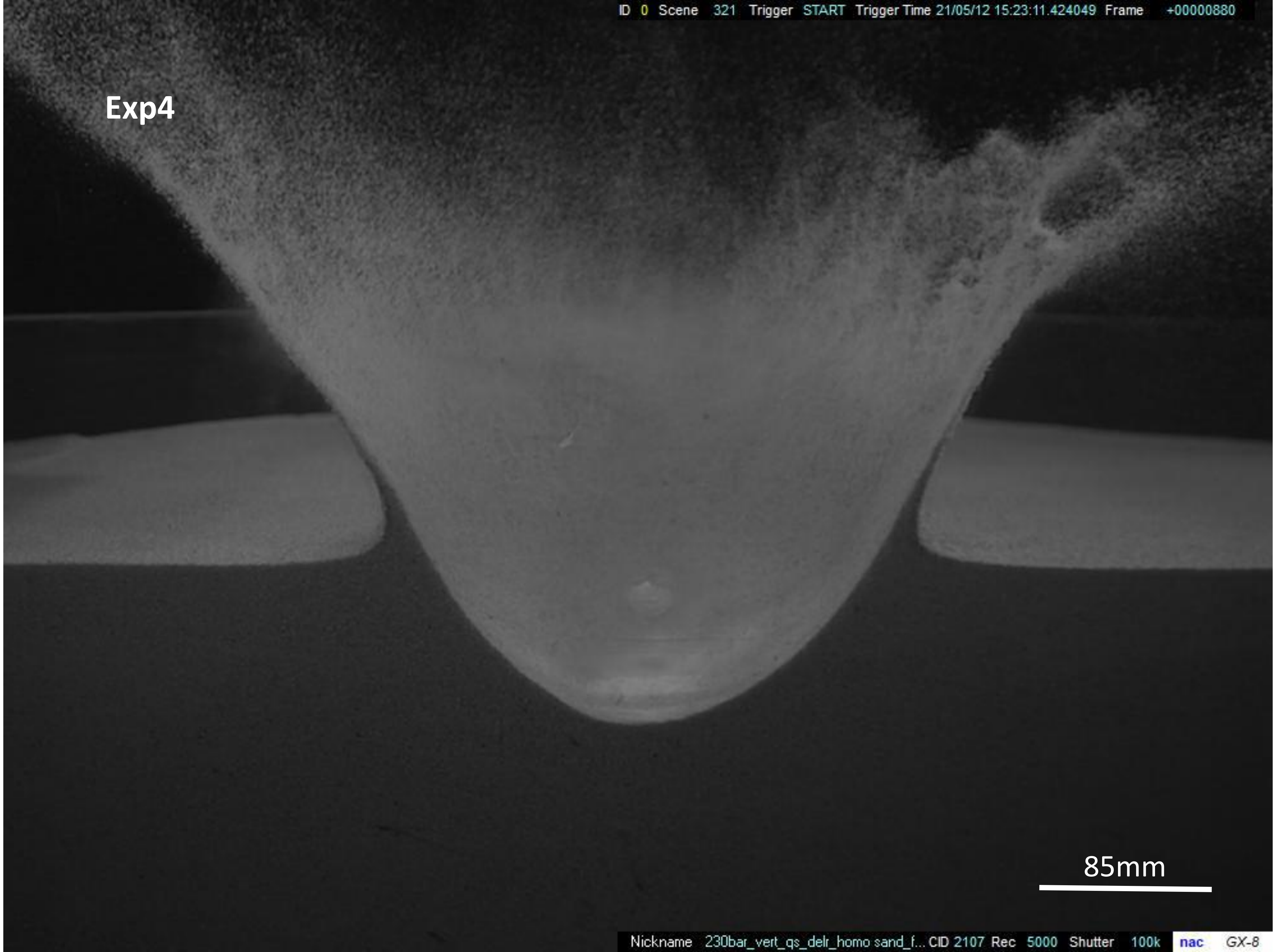
ID 0 Scene 321 Trigger START Trigger Time 21/05/12 15:23:11.424049 Frame +00000880
Exp4
85mm
Nickname 230bar_vert_qs_delr_homo sand_f... CID 2107 Rec 5000 Shutter 100k nac GX-8

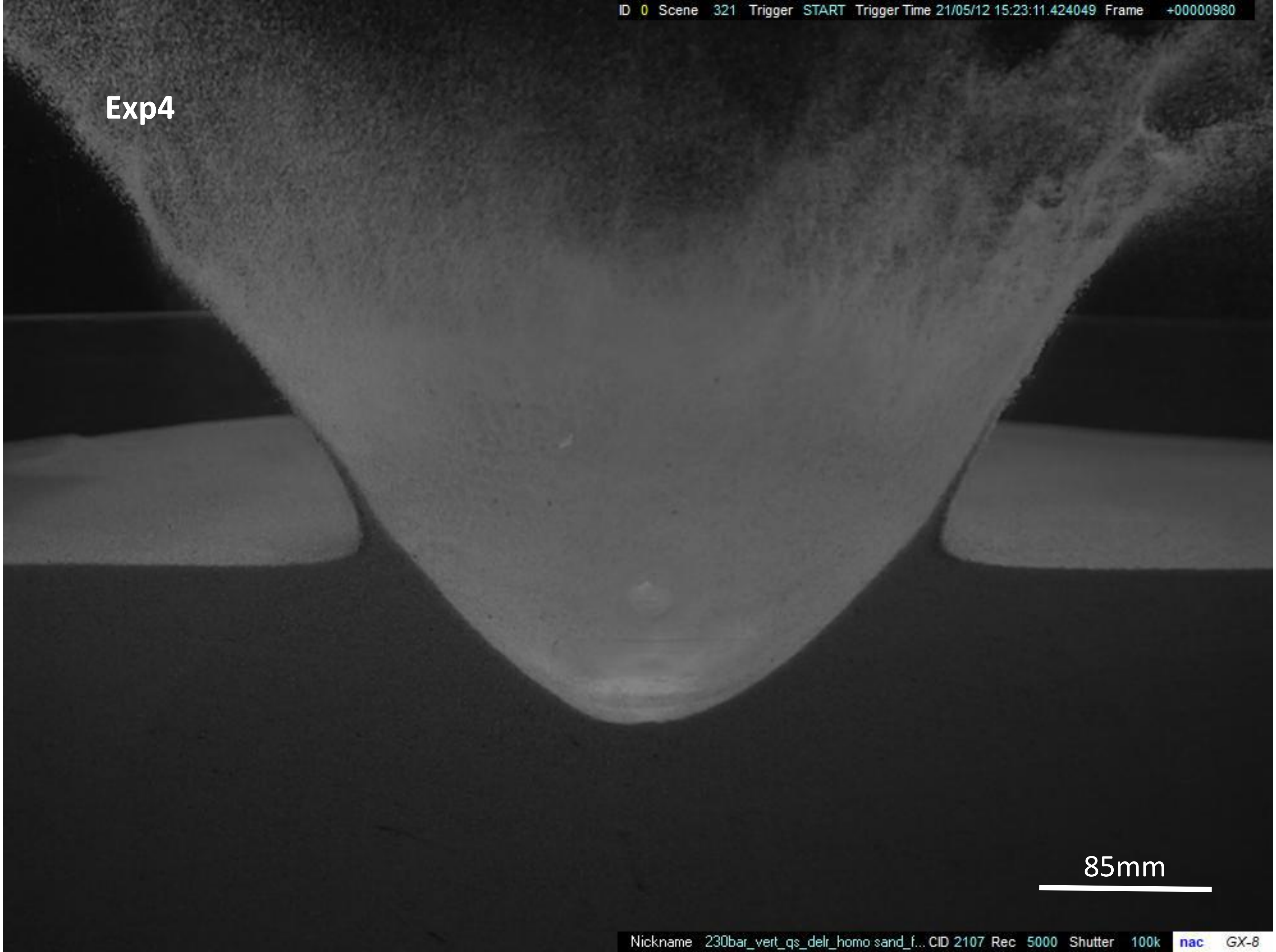
ID 0 Scene 321 Trigger START Trigger Time 21/05/12 15:23:11.424049 Frame +00000980
Exp4
85mm
Nickname 230bar_vert_qs_delr_homo sand_f... CID 2107 Rec 5000 Shutter 100k
nac GX-8

T: 0.099sec

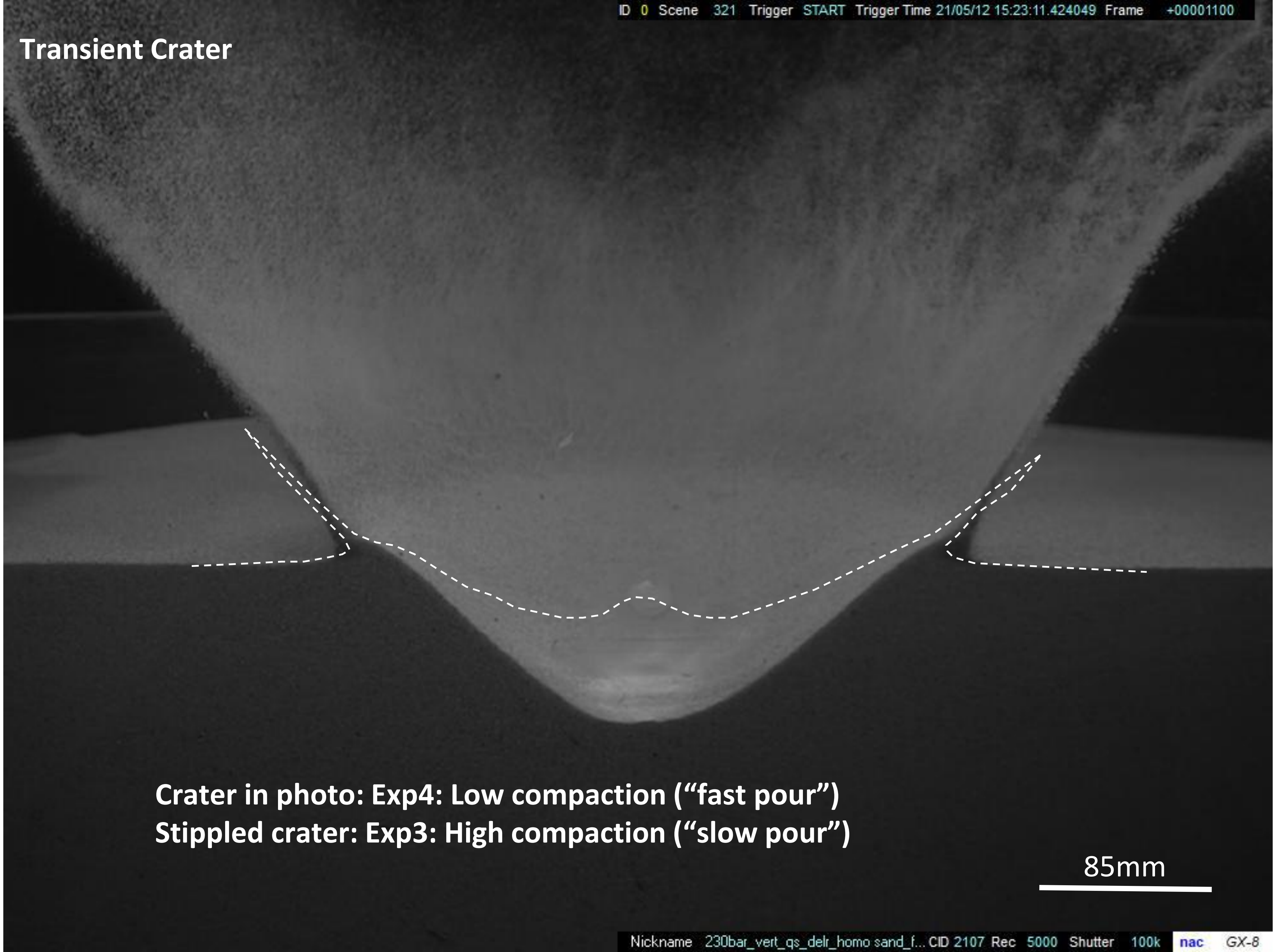
Transient Crater
ID 0 Scene 321 Trigger START Trigger Time 21/05/12 15:23:11.424049 Frame +00001100
Crater in photo: Exp4: Low compaction (“fast pour”)
Stippled crater: Exp3: High compaction (“slow pour”)
85mm
Nickname 230bar_vert_qs_delr_homo sand_f... CID 2107 Rec 5000 Shutter 100k nac GX-8

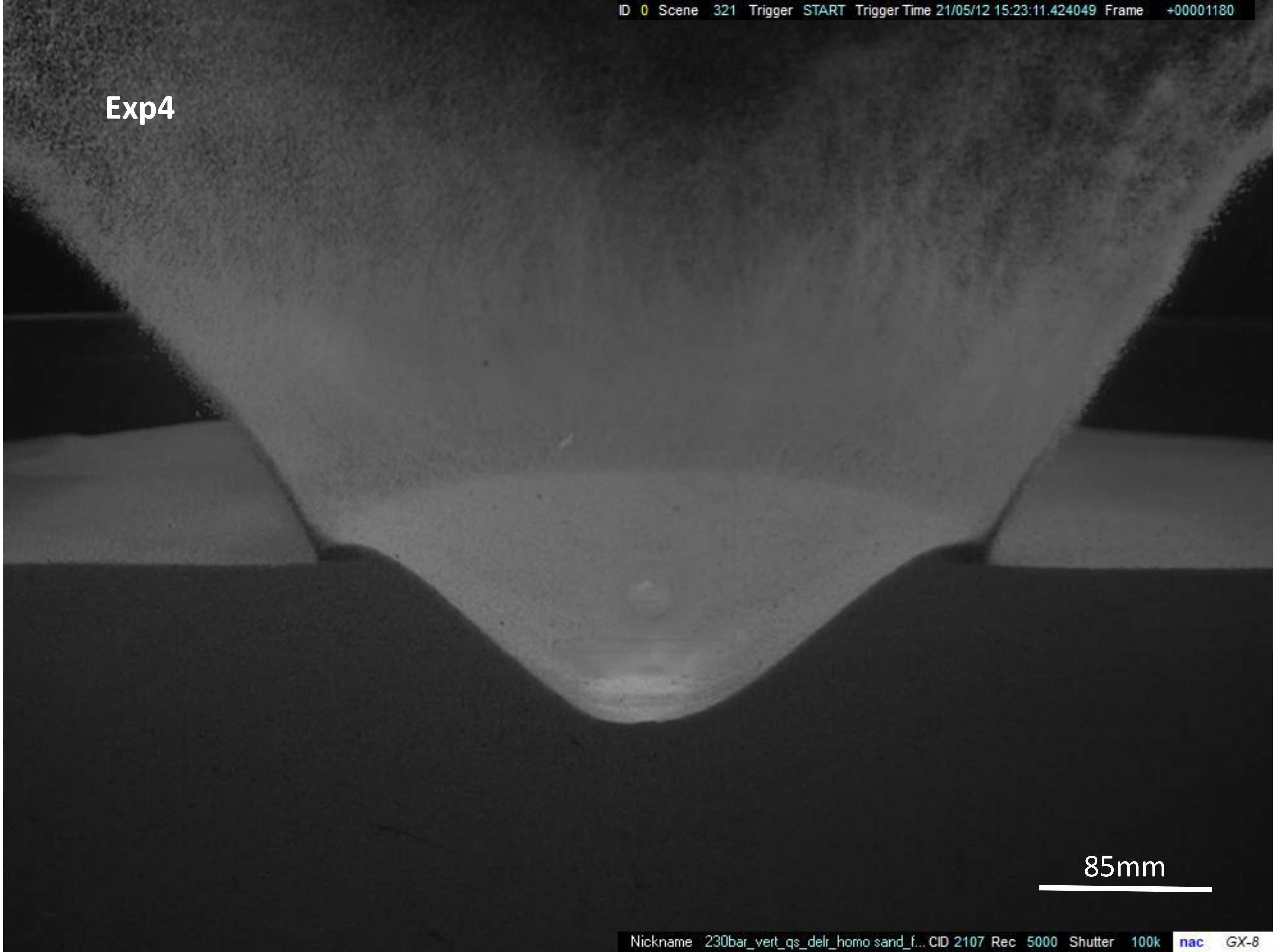
ID 0 Scene 321 Trigger START Trigger Time 21/05/12 15:23:11.424049 Frame +00001180
Exp4
85mm
Nickname 230bar_vert_qs_delr_homo sand_f... CID 2107 Rec 5000 Shutter 100k nac GX-8

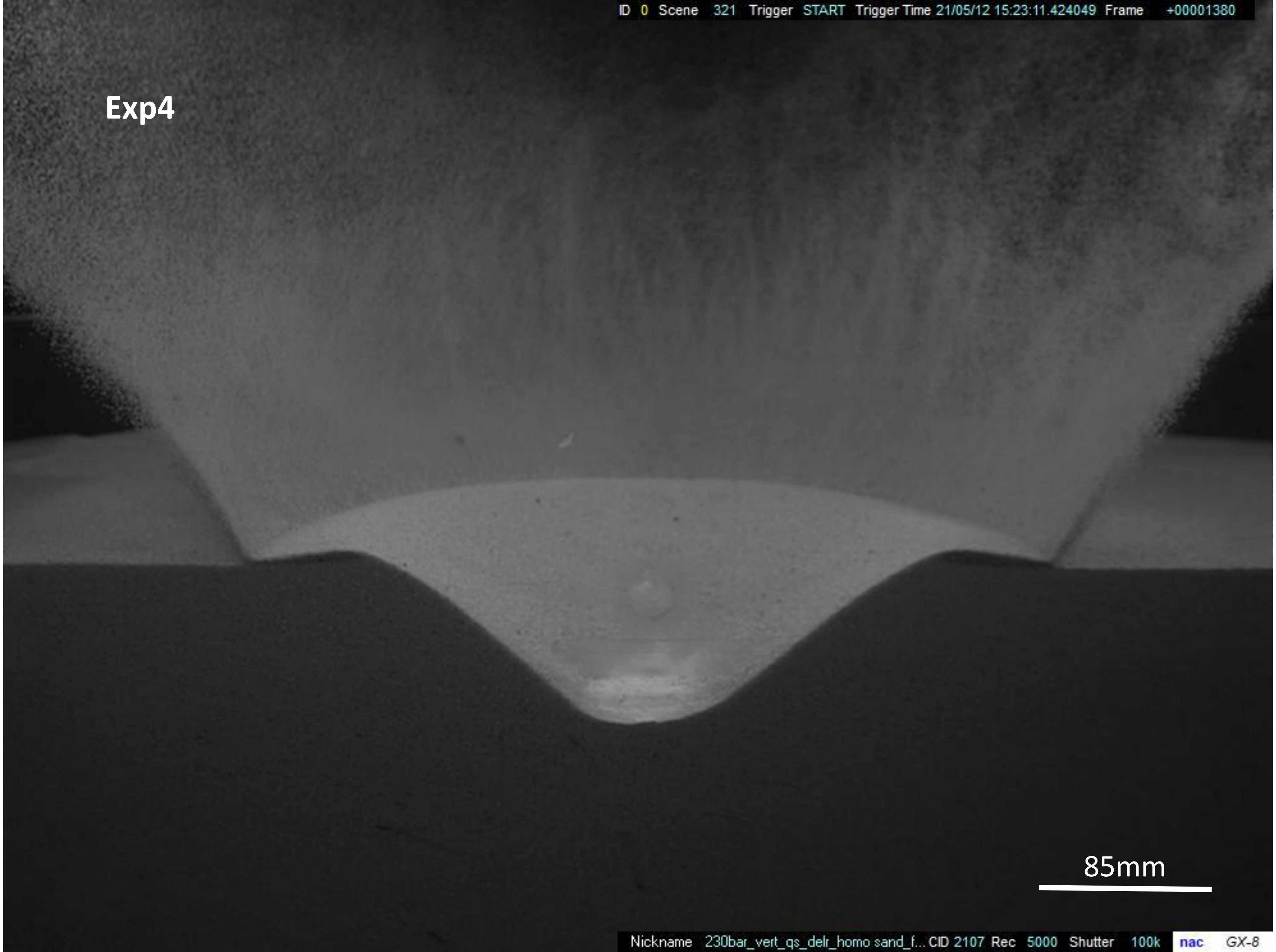
ID 0 Scene 321 Trigger START Trigger Time 21/05/12 15:23:11.424049 Frame +00001380
Exp4
85mm
Nickname 230bar_vert_qs_delr_homo sand_f... CID 2107 Rec 5000 Shutter 100k nac GX-8

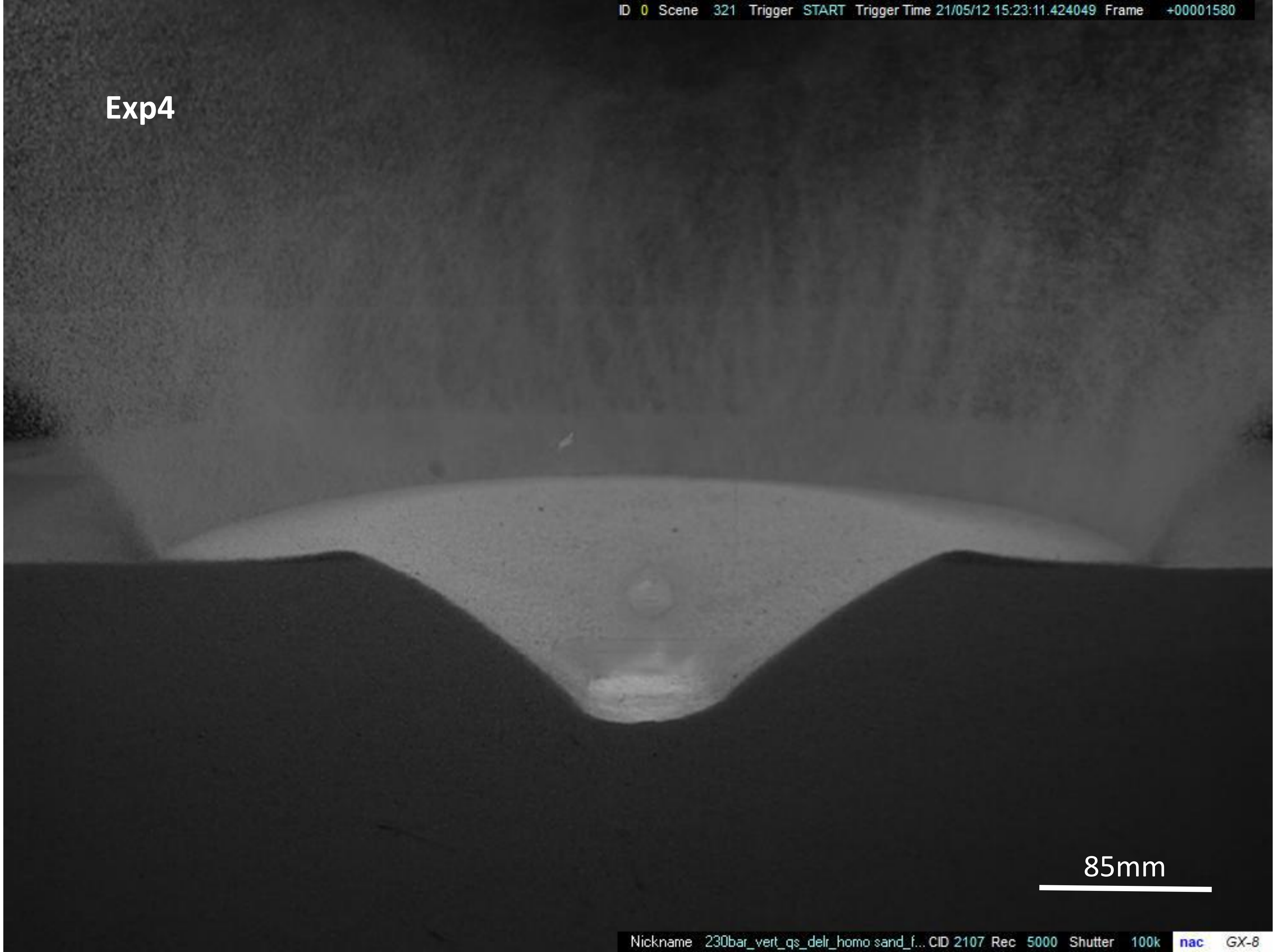
ID 0 Scene 321 Trigger START Trigger Time 21/05/12 15:23:11.424049 Frame +00001580
Exp4
85mm
Nickname 230bar_vert_qs_delr_homo sand_f... CID 2107 Rec 5000 Shutter 100k nac GX-8

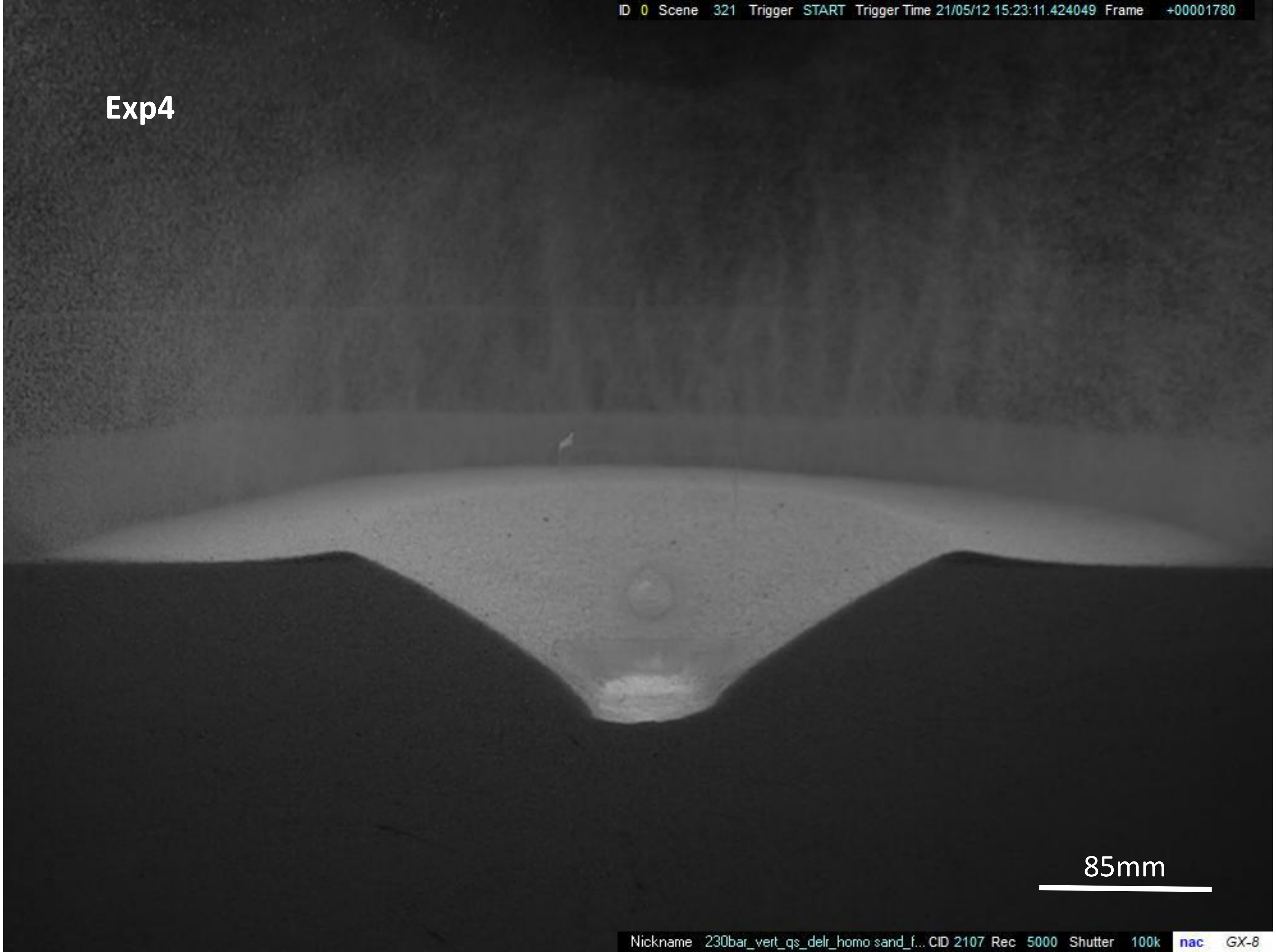
ID 0 Scene 321 Trigger START Trigger Time 21/05/12 15:23:11.424049 Frame +00001780
Exp4
85mm
Nickname 230bar_vert_qs_delr_homo sand_f... CID 2107 Rec 5000 Shutter 100k nac GX-8

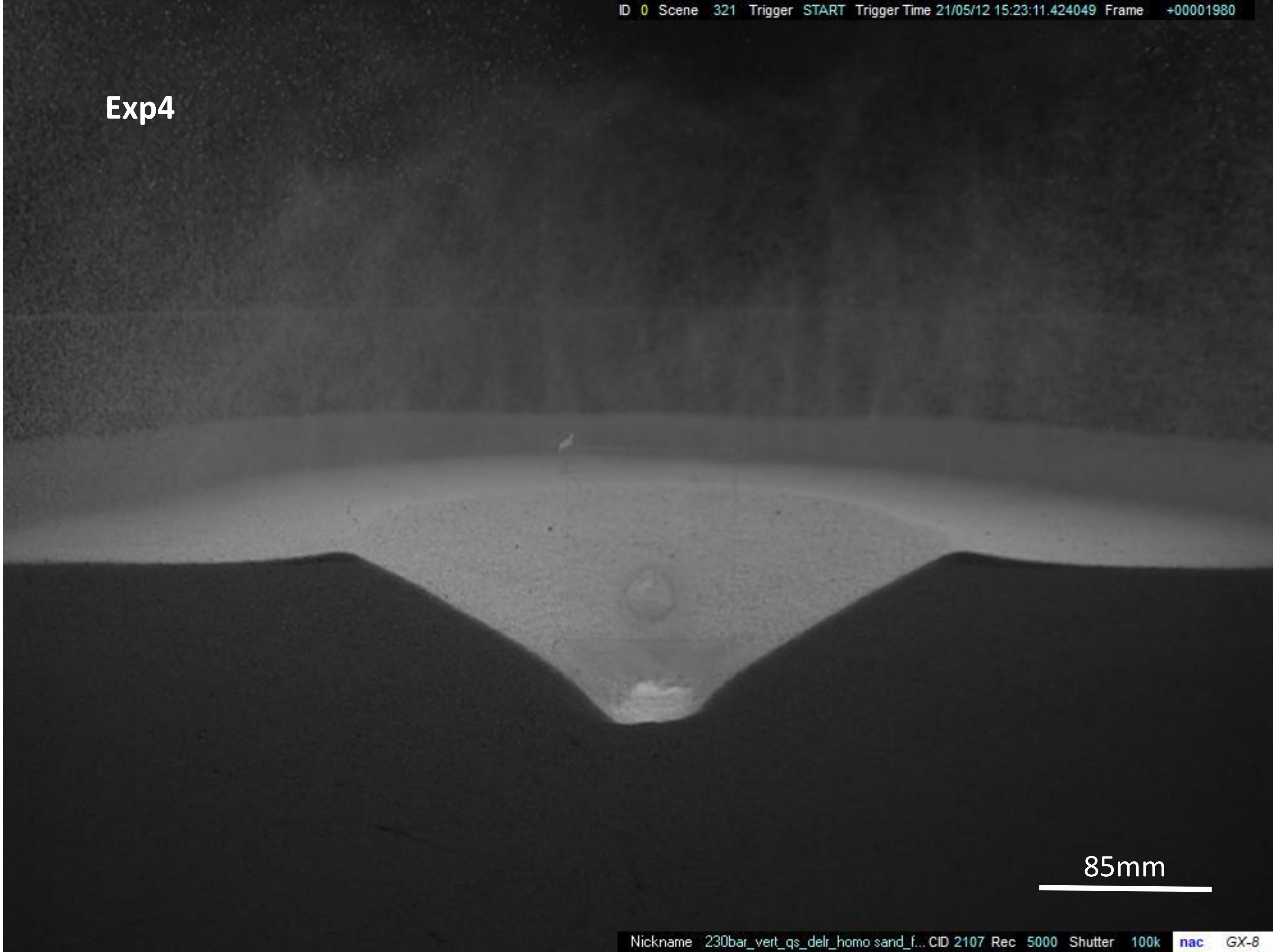
ID 0 Scene 321 Trigger START Trigger Time 21/05/12 15:23:11.424049 Frame +00001980
Exp4
85mm
Nickname 230bar_vert_qs_delr_homo sand_f... CID 2107 Rec 5000 Shutter 100k
nac
GX-8

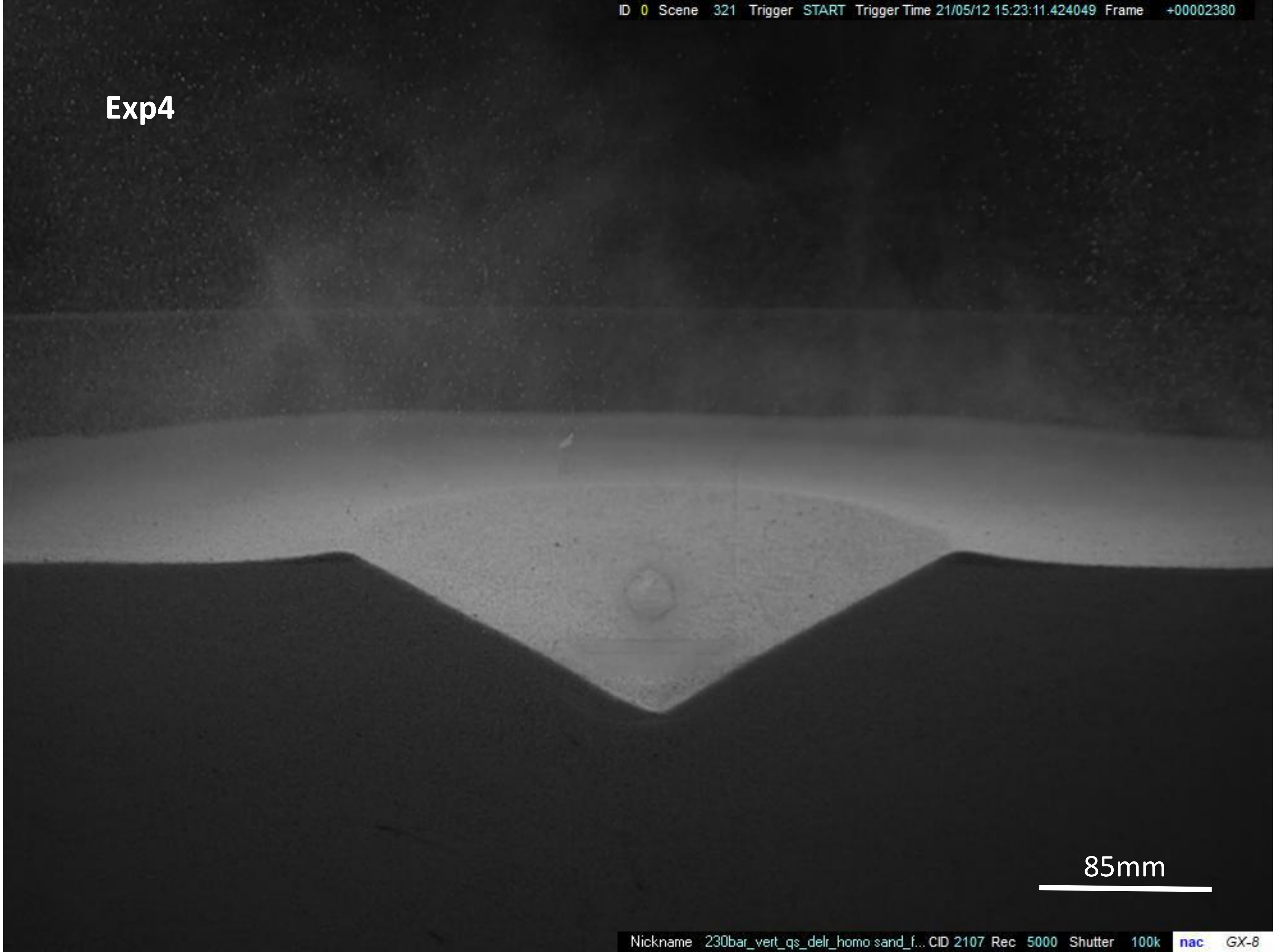
ID 0 Scene 321 Trigger START Trigger Time 21/05/12 15:23:11.424049 Frame +00002380
Exp4
85mm
Nickname 230bar_vert_qs_delr_homo sand_f... CID 2107 Rec 5000 Shutter 100k
nac GX-8

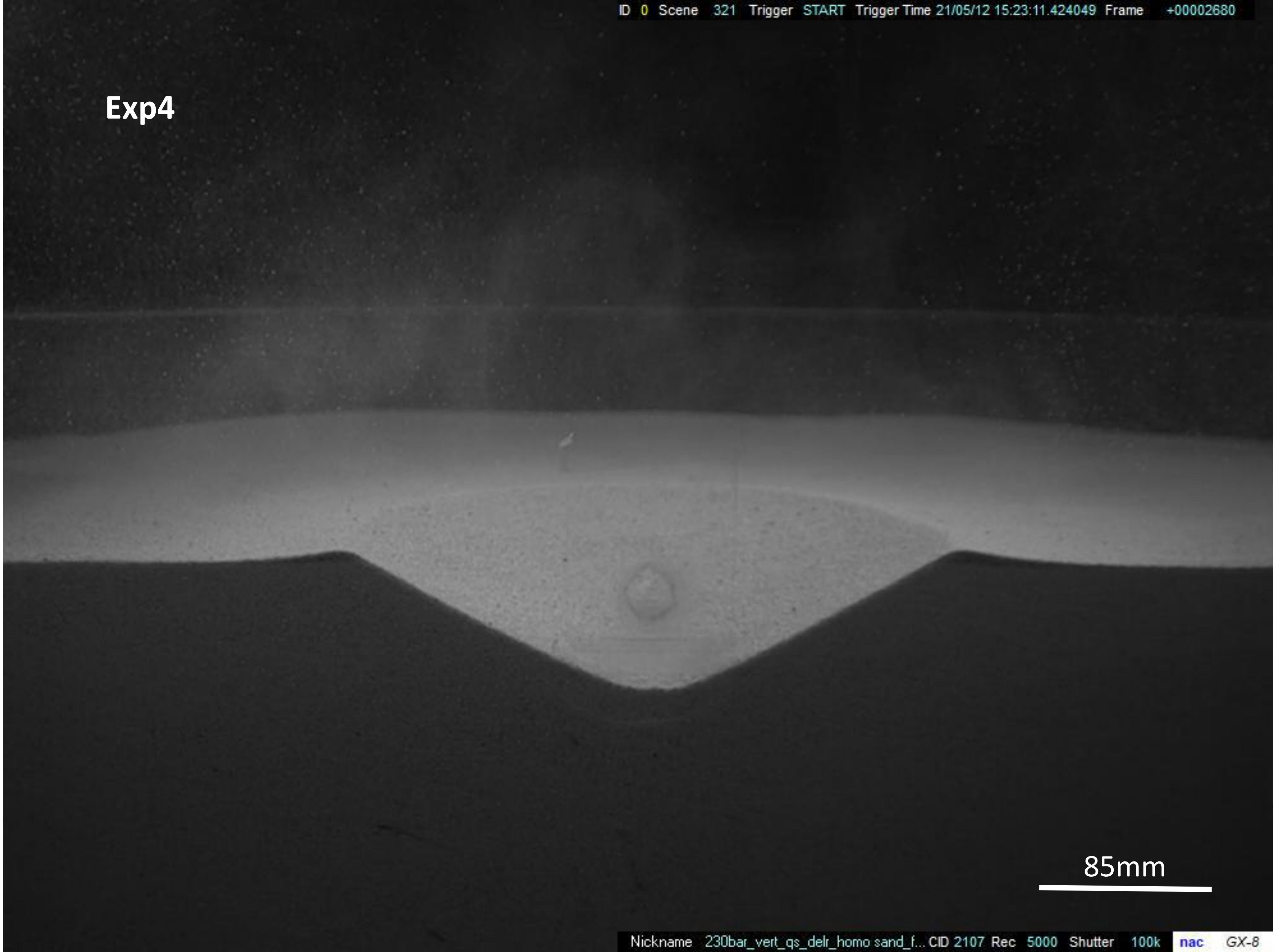
ID 0 Scene 321 Trigger START Trigger Time 21/05/12 15:23:11.424049 Frame +00002680
Exp4
85mm
Nickname 230bar_vert_qs_delr_homo sand_f... CID 2107 Rec 5000 Shutter 100k nac GX-8

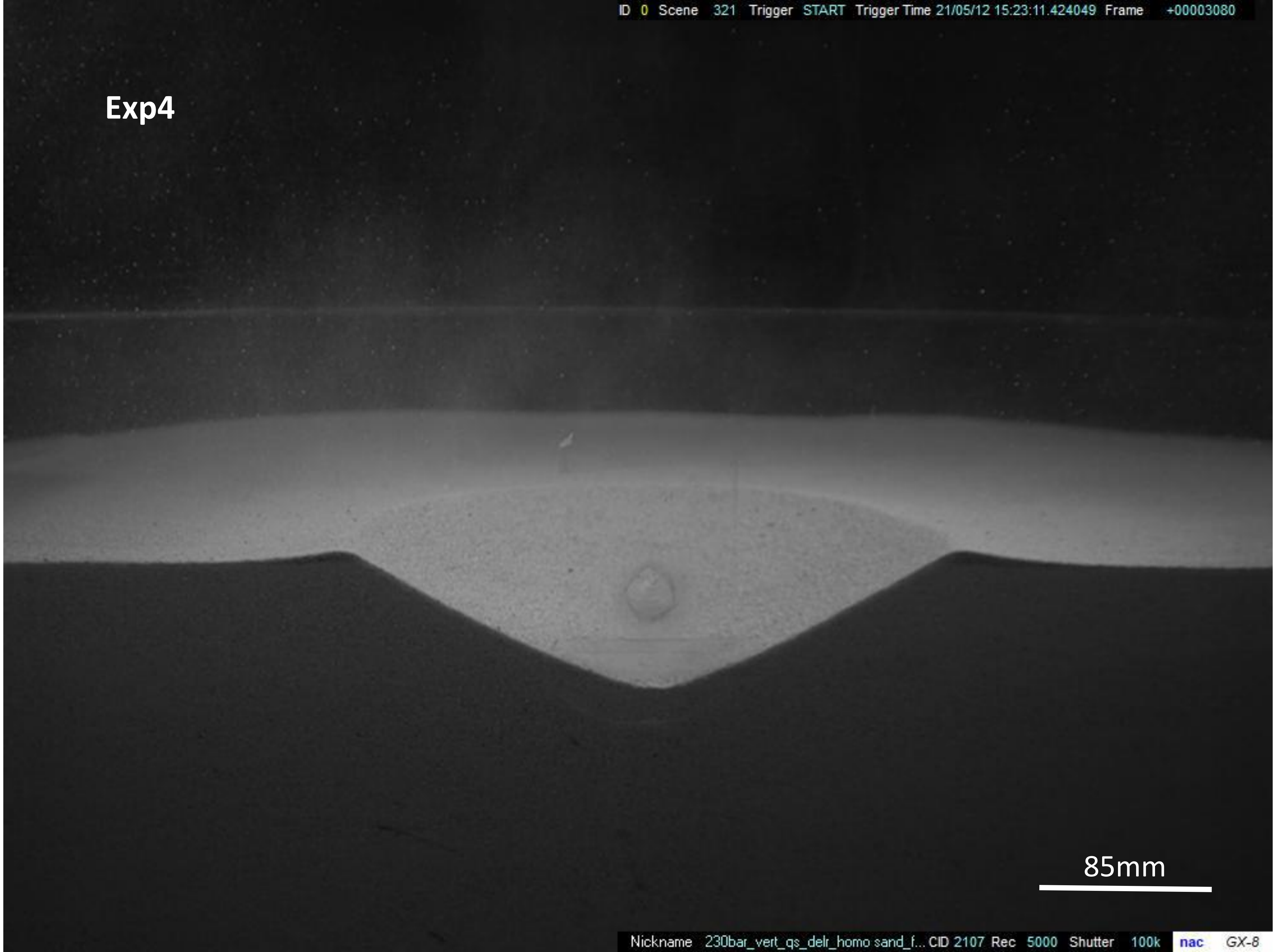
ID 0 Scene 321 Trigger START Trigger Time 21/05/12 15:23:11.424049 Frame +00003080
Exp4
85mm
Nickname 230bar_vert_qs_delr_homo sand_f... CID 2107 Rec 5000 Shutter 100k nac GX-8

(T: >0.699sec)

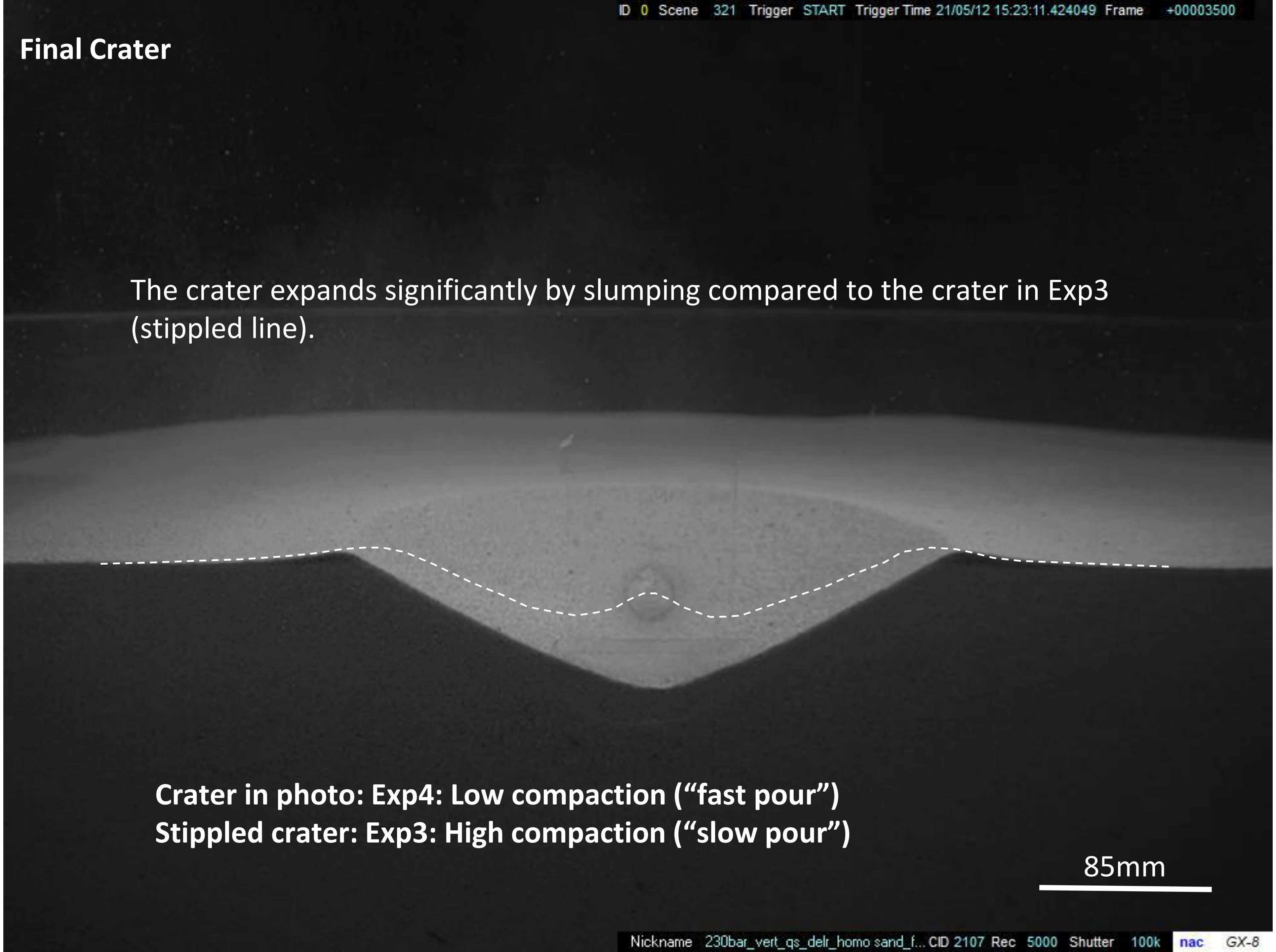
ID 0 Scene 321 Trigger START Trigger Time 21/05/12 15:23:11.424049 Frame +00003500
Final Crater
The crater expands significantly by slumping compared to the crater in Exp3 (stippled line).
Crater in photo: Exp4: Low compaction (“fast pour”)
Stippled crater: Exp3: High compaction (“slow pour”)
85mm
Nickname 230bar_vert_qs_delr_homo sand_f... CID 2107 Rec 5000 Shutter 100k nac GX-8

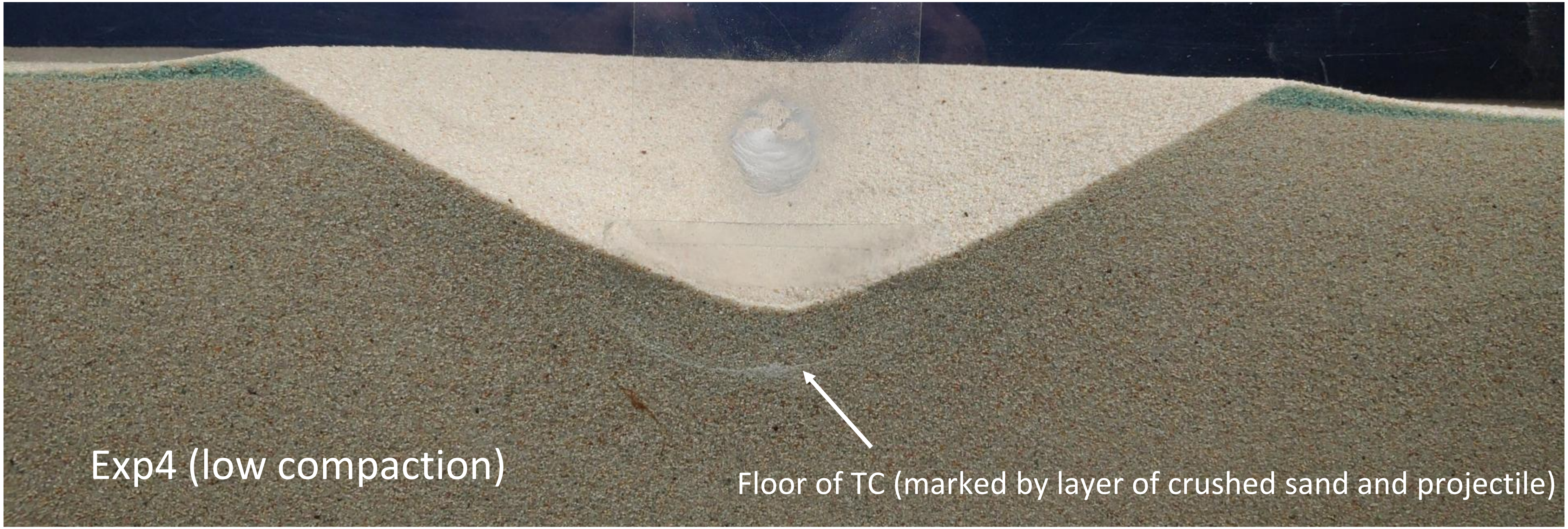
Exp4 (low compaction)
Floor of TC (marked by layer of crushed sand and projectile)

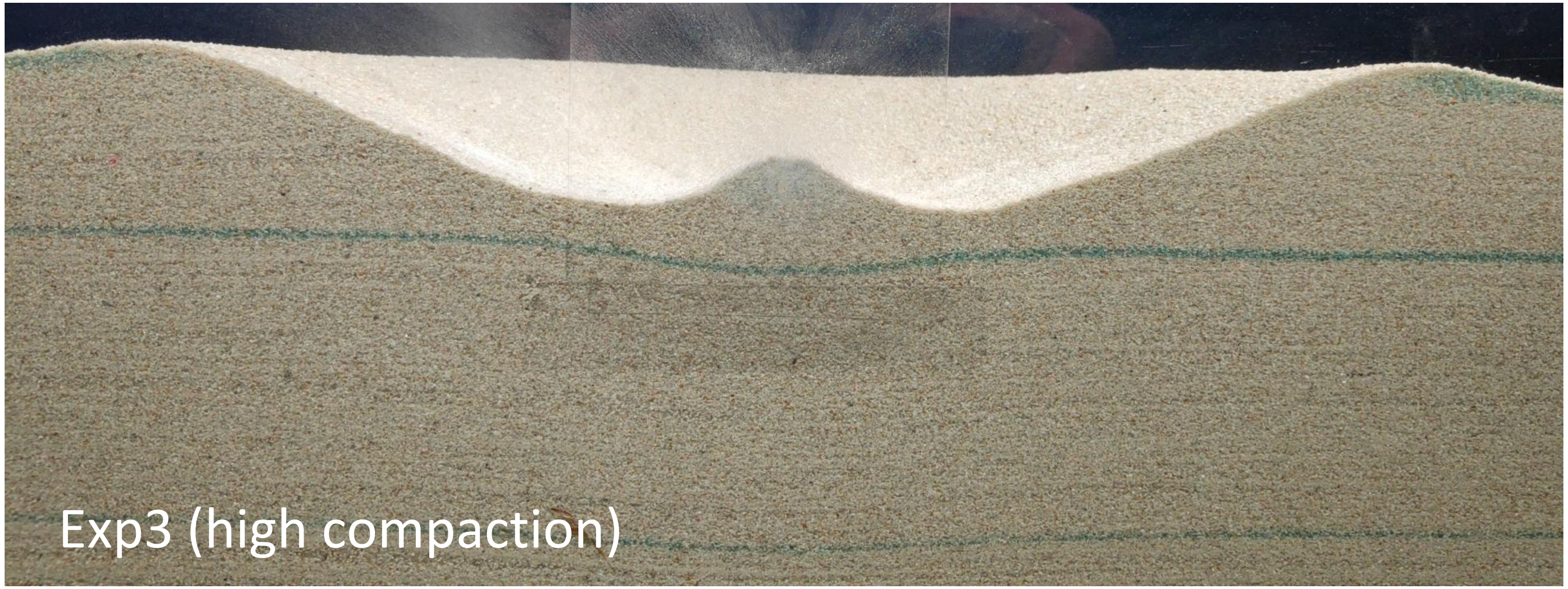
Exp3 (high compaction)

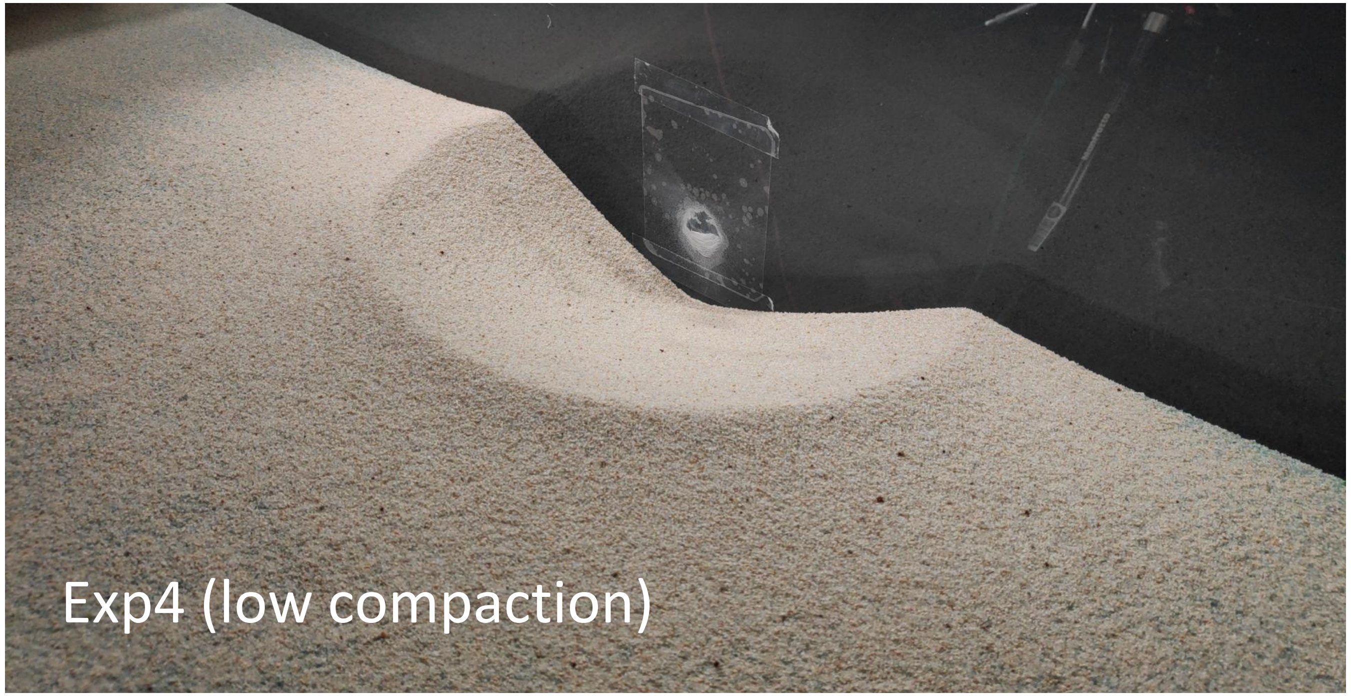

The small ‘hump’ at the crater center in Experiment 3, is not a central peak known from natural complex craters nor is it a ‘central mound’ due to target layering.

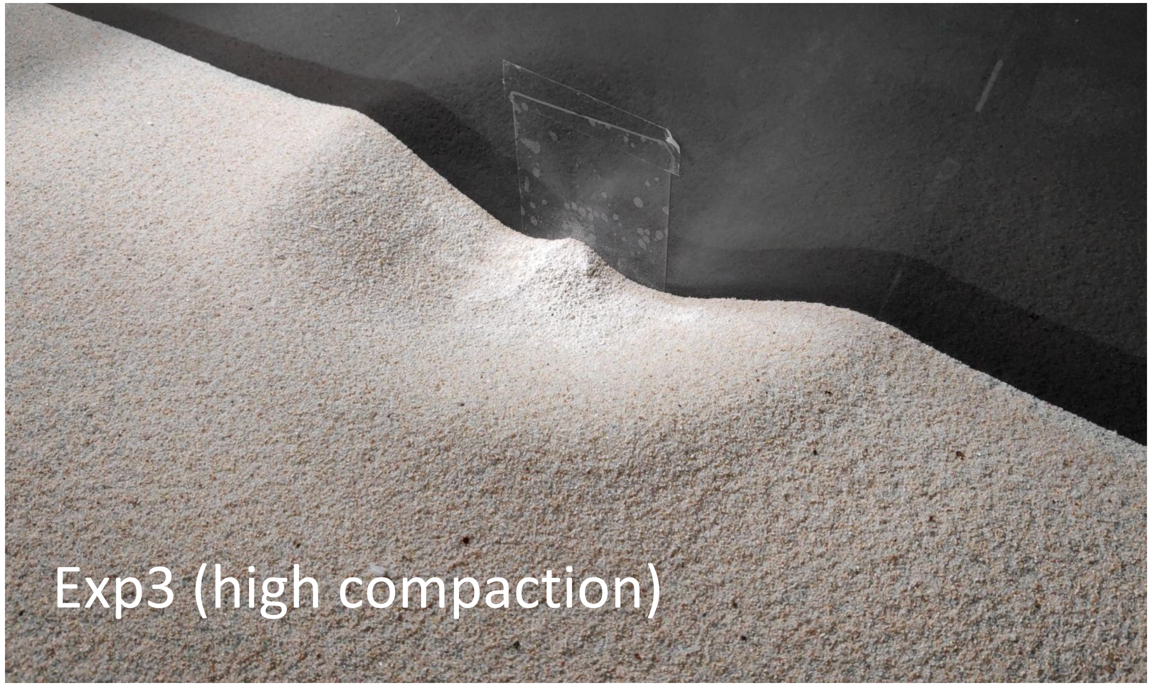

Supplement: Supplementary file 1 — Supplementary Information. [file 41598_2026_39893_MOESM1_ESM.pdf]
